# Standardising bee sampling: A systematic review of pan trapping and associated floral surveys

**DOI:** 10.1002/ece3.11157

**Published:** 2024-03-17

**Authors:** André Krahner, Anke C. Dietzsch, Tobias Jütte, Jens Pistorius, Jeroen Everaars

**Affiliations:** ^1^ Julius Kühn Institute (JKI) – Federal Research Centre for Cultivated Plants, Institute for Bee Protection Braunschweig Germany

**Keywords:** bee sampling, floral survey, monitoring, pan trap, sampling bias, sampling standardisation

## Abstract

The use of coloured pan traps (bee bowls, Moericke traps) for sampling bees (and other pollinators) has continuously increased over the last two decades. Although a number of methodological studies and conceptual frameworks offer guidance on standardised sampling, pan trap setups vary widely in characteristics even when optimised for capturing bees. Moreover, some uncertainty persists as to how local flower abundance and diversity influence sampling. We systematically reviewed peer‐reviewed studies that used pan traps for bee collection and that were listed in the Web of Science core collection. To gauge methodological variation, we identified a set of relevant methodological criteria and assessed the studies accordingly. For obtaining evidence that pan trap samples and floral environment around traps are correlated, we screened the relevant studies for such correlations. While some aspects of pan trapping (e.g., trap coloration and elevation) were similar in the majority of studies, other aspects varied considerably (e.g., trap volume/diameter and sampling duration). Few studies used floral abundance and/or diversity as an explanatory variable in their analyses of bee samples. Among these studies, we found a considerable variation in key aspects of floral survey methods, such as time and space between vegetation surveys and pan trap sampling, abundance measures (quantitative, semi‐quantitative and presence–absence), and processing of raw data prior to analysis. Often studies did not find any correlation between the floral environment and bee samples. Reported correlations varied markedly across studies, even within groups of studies applying a similar method or analysing a similar group of bees. Our synthesis helps to identify key issues of further standardisation of pan trap methodology and of associated floral surveys. In addition to the few aspects that have been standardised over the past decades, we suggest methodological direction for future research using pan traps as a better standardised method for the collection of wild bees. We encourage further studies to illuminate if and how varying floral resources around traps bias bee samples from pan traps. More generally, our synthesis shows that trapping methodologies should be reviewed regularly when their use increases to ensure standardisation.

## Graphical abstract

Although a number of methodological studies and conceptual frameworks offer guidance on standardised sampling, pan trap setups vary widely in characteristics. To gauge methodological variation, we systematically reviewed peer‐reviewed studies that used pan traps for bee collection and assessed the studies using a set of relevant methodological criteria. For obtaining evidence that pan trap samples and floral environment around traps are correlated, we screened the relevant studies for such correlations.

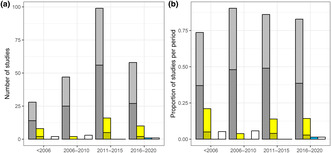

## 1 INTRODUCTION

Pan traps are an effective method for sampling bee communities (Westphal et al., 2008), and they play a key role in current bee monitoring programmes (Droege et al., 2016; Kammerer et al., 2020; Potts et al., 2021; but see Portman et al., 2020). One huge benefit of pan traps over hand netting is the avoidance of observer bias associated with hand netting (Krahner et al., 2021; Packer & Darla‐West, 2021) and the possibility to sample at multiple locations nearly simultaneously, that is, under the same weather conditions and during the same phenological state. Thus, pan traps inherently provide a high degree of methodological standardisation within a study and possibly even among studies, yielding comparable data from different locations. However, like other trap systems used for bee sampling, such as trap nests and Malaise traps, there are many ways to modify pan traps, based on the research objectives, study design and resource availability. Although several attempts have been made to standardise pan trap design in order to increase comparability among different studies (e.g., Carvell et al., 2016; Droege et al., 2016; LeBuhn et al., 2003), various modification options have not been reviewed systematically. Standardisation of survey methods remains a challenge for bee monitoring programmes in general (Woodard et al., 2020). In order to summarise the options available for pan trap modifications, and to identify routes towards methodological standardisation, we systematically reviewed the published literature. We identified parameters with potential for standardisation falling into two categories: the traps themselves and the assessment of floral context around traps. Standardising these parameters should ultimately result in a standardisation of sampling effort and hence comparability of data across studies in space and time.

### 1.1 Standardisation of pan traps

Pan traps differ from other methods mainly because they have an attracting colour, similar to flowers, and fool bees, which drown when they try to “land on the flower”. Pan trap colours differ in attractiveness, and several wild bee species show colour preferences (Grundel et al., 2011; Krahner et al., 2021; Leong & Thorp, 1999). In addition to the visible light spectrum emitted by traps, UV characteristics appear to influence sampling results (Droege, 2005; but see Prendergast et al., 2020), and ‘UV‐bright’ colours are often used to increase trap attractiveness (e.g., Westphal et al., 2008). We expect to find some consensus on what colours are best to use when reviewing the literature on pan traps, acknowledging that the bee community of interest may be the crucial factor in choosing colours.

Despite being a critical experimental variable, relatively little is known about the effects of trap size on sampling results (Gonzalez et al., 2020; Wilson et al., 2016). In theory, larger pan trap surfaces should attract more bees due to stronger stimuli. Until recently, researchers used various pan trap sizes (Gonzalez et al., 2020). With repeated attempts to standardise pan trap size (Droege et al., 2016; LeBuhn et al., 2003, 2016), and with Gonzalez et al. (2020) considering only studies published during a short time period in their literature review, it seems to be time for an updated and more comprehensive review of trap sizes.

The typical pan trap liquid consists of water with a surfactant. The pivotal function of the liquid is to retain the specimen in the trap. Additional functions may include preservation of sampled individuals and olfactory attractiveness (scent). To a certain extent, the choice of trapping liquid composition is determined by sampling duration and weather conditions, since longer, sun‐exposed sampling with higher evaporation potential may require other liquids than short‐term sampling events (Droege et al., 2016).

When choosing a trap design, the distance between the sub‐units (Droege et al., 2010) and the position of the pan trap (Wheelock & O'Neal, 2016) considerably contribute to its capture probability. Different guilds of bees may forage at different horizontal strata (Gumbert & Kunze, 1999) and may be caught according to their preferred height. Pan traps placed within the flower stratum sample more bee individuals and a different composition of bees compared to pan traps above or below (Geroff et al., 2014; Tuell & Isaacs, 2009; Wheelock & O'Neal, 2016). When using pan traps in tree canopies (Bąk‐Badowska, 2012; Campbell et al., 2018; Li et al., 2021; Urban‐Mead et al., 2021), trap position may be even more important for bee capture rates than trap colour.

### 1.2 Floral survey methodologies

Floral context (flower density and diversity) around pan traps is assumed to have an effect on pan trapping results for hymenopterans (Saunders & Luck, 2013), and the resulting bias of pan trap samples of bees has been debated recently (Portman et al., 2020; Prendergast & Hogendoorn, 2021). Accordingly, the potential impact of this floral context on bee sampling results is of particular interest for large‐scale and long‐term monitoring schemes (Carvell et al., 2016; Potts et al., 2021). Several studies indicate that pan trapping of bees is particularly effective when floral resources around pan traps are scarce (e.g., Wilson et al., 2008). Evidence to support this potential bias is mostly of observational nature rather than a result of experimental investigation. For the sake of data comparability, some authors suggest to limit pan trapping to habitats with similar floral resources (e.g., Roulston et al., 2007; Templ et al., 2019) or to environments without flowers (e.g., Cane et al., 2000). Alternatively, statistical analysis may account for variation in floral resources (e.g., Bergholz et al., 2022; Kovács‐Hostyánszki et al., 2013). If so, it is important to use standardised sampling methods to evaluate flower abundance and diversity. Vegetation ecologists sample plants in various ways (Chytrý & Otýpková, 2003) that are often time‐consuming. Bee researchers may have to allocate sampling time differently, focus on floral data relevant for bees, and modify methodologies for their needs. We aim to compare floral assessment methods and to evaluate the integration of floral resource measures (abundance and diversity) into bee abundance and diversity analyses. Ultimately, we present an unbiased assessment of correlations between flower and bee abundance and diversity.

This study provides a synthesis of (1) common practices of pan trap methodologies, (2) practices of floral survey methodologies used in pan trap studies, and (3) the relationship between bee samples from pan traps and the floral environment. The ultimate aim of this paper is to suggest a way of sensible standardisation and to advise future pan trap studies.

## 2 MATERIALS AND METHODS

We used a systematic approach for this review (Mengist et al., 2020). First, we searched the Web of Science (WOS) Core Collection using the following search string (without restrictions on publication year): TS = ((bee OR bees OR bumblebee OR bumblebees OR *bee OR *bees OR apidae OR apoidea OR honeybee OR honeybees OR honey OR mellifera)) AND TS = (pan trap OR pan trap* OR bowl OR bowl* OR Moericke). The final run of the search was on 13 April 2022. We identified post‐hoc that many studies using pan traps were not included in the search results, because the authors only mention the phrase “pan trap” in their methods section rather than in a WOS indexed field. In order to ensure full search reproducibility (Pozsgai et al., 2021), we proceeded with the systematic approach and additionally provide the complete list of references (Table S1).

We successively screened title, abstract and full text of the resulting studies, removing irrelevant studies (i.e., studies not concerning bees and/or not using pan traps and/or not written in English) and meeting abstracts from the dataset. Using an a‐priori defined set of criteria (Table S3), we extracted all relevant information from the full text of the remaining studies. Duplicate studies, that is, studies explicitly indicating a repeated use of the same data set, were omitted from analysis (Table S1). A few studies with striking similarities in their methods section, but no information on earlier use of data were retained in the analysis. When a publication contained multiple studies, each of them reporting different sampling procedures, each study was considered separately.

Shades of a specific colour were pooled within the basic colour in the analysis (e.g., light and dark blue were considered as blue). Approximations of continuous variables (specifically diameter, volume, height, distance) were noted as their exact values (e.g., 10 cm instead of “approximately 10 cm”), because many studies did not report measurement inaccuracy. Studies with ambiguous details about their methods were included in our analyses only with those parts of data for which collection methods were sufficiently described. For studies reporting multiple diameters of pan traps (such as upper and bottom diameter, to describe pan trap shape), we used the upper rim diameter in the analysis. We regarded the terms “surfactant” and “detergent” for the trap liquid as synonyms.

We created a subset of relevant studies that statistically analysed relationships between floral resources (floral diversity and abundance) and bees sampled by pan traps (number of collected bees, i.e. “abundance”, and collected bee diversity, i.e., “bee diversity”). All these studies reported methodological details on sampling of floral resources and an analysis of their effects on bee abundance and/or diversity. Studies pooling pan trap samples with other collection methods and/or combining sampling results for bees and other taxa prior to analysis as well as studies reporting vegetation surveys without floral data (for example plant cover data) were omitted.

## 3 RESULTS

The literature search yielded 369 publications, including seven duplicates and 88 irrelevant publications (Table S1). The remaining 274 publications contained 290 relevant pan trap case studies. These relevant studies were spread unevenly across the globe, with the majority conducted in North America (145 studies) and Europe (82; South & Central America: 22; Africa: 16; Asia: 13; Australia & New Zealand: 12). The most common shape of the bowls was round, with a few studies using rectangular or hexagonal containers as pan traps. The complete relevant information extracted from the references is given in Table S2.

### 3.1 Standardisation of pan traps

#### 3.1.1 Pan coloration

**FIGURE 1:**
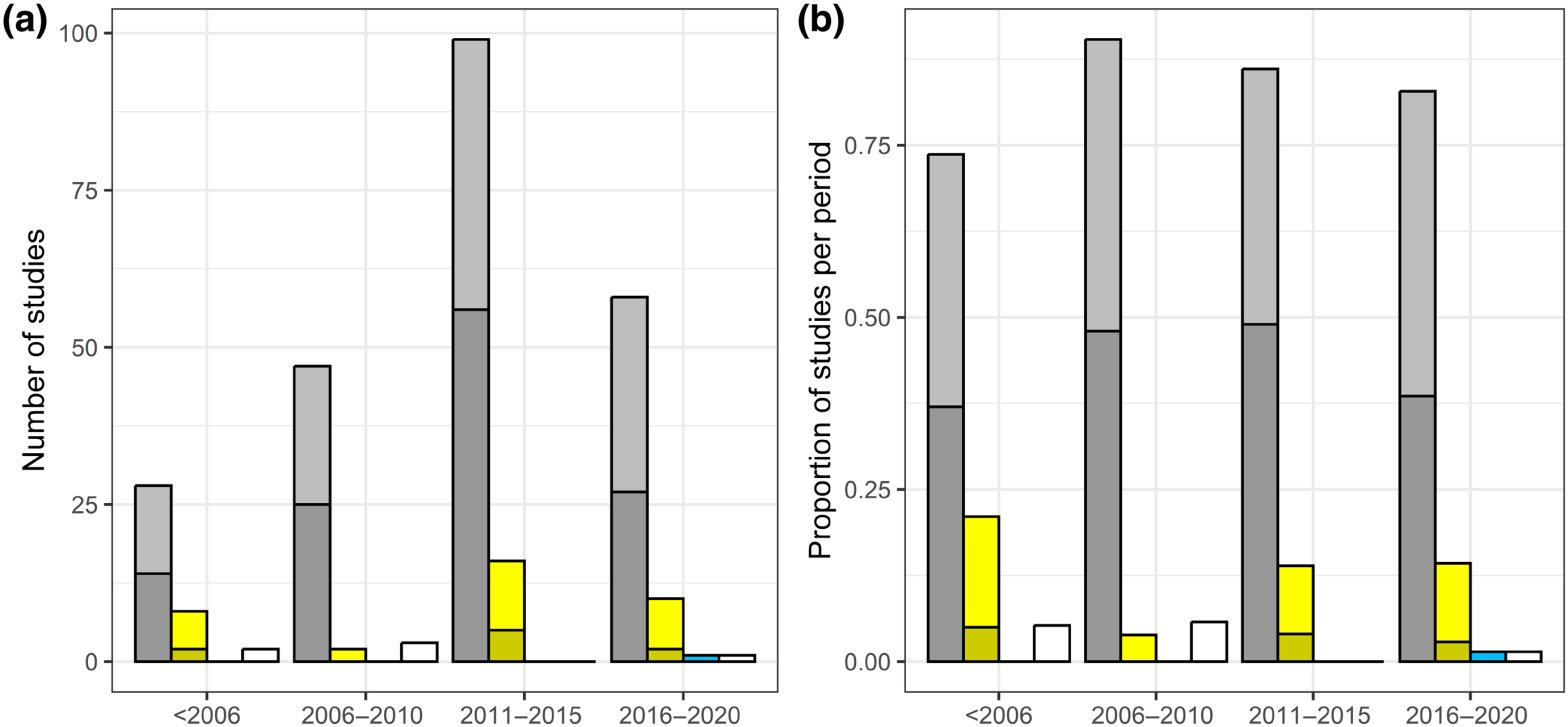
Absolute (a) and relative (b) number of studies (*n* = 275) using pan traps with single colours (yellow, blue, white; represented by coloured bars) or combinations of two or more colours (grey bars). Darker shadings represent the proportion of studies mentioning fluorescence and/or UV reflection (none for single white and blue colours). For multi‐year studies, the first study year was used.

For decades, colour combinations have been the prevailing approach compared to single‐coloured pan traps (Figure 1). Forty‐four studies used only one colour (37 yellow, 6 white, 1 blue), while the majority of studies (199) used a combination of three colours, usually yellow, blue, and white (193). Few studies used a combination of more than three colours (20) and/or other colours (26), including red, orange, green, purple, violet, pink, magenta, turquoise, beige, black and clear pan trap surfaces. 98 studies provided reproducible details on colour manufacturer and/or characteristics (in case of unpainted traps often by providing details on pan manufacturer/supplier and/or model), and 12 studies referred to details on spectral reflectance in UV and/or visible wavelength ranges. In these studies, main peaks in relative reflectance diverged less than 50 nm (yellow) or 100 nm (yellow UV). Main peaks in relative reflectance for blue and blue UV traps were virtually identical, while white and white UV traps did not generally show marked peaks; light was reflected rather uniformly in a range between approximately 400 and 800 nm. Less than half of the studies that report the colours used for pan traps (136 out of 287) mention the use of UV colours. Of these studies, 88 mention fluorescence, while 26 mention UV reflection.

Traps of some studies differed in where they were coloured. Of the 36 studies that applied paint to bowls and provided statements or photos on colour position, 14 studies used pan traps coloured with the attracting colour both on the interior and the exterior surface. The remaining 22 (out of 36) studies reported pan traps with the attracting colour on the interior surface only, the exterior surface being white (13 studies), yellow, brown, green or black (one study, respectively; one study using both traps white and green on the outside).

Among the 243 studies using sets of pan traps with more than one colour, 163 stated the distance among differently coloured traps. About half of these studies (82 out of 163) used distances of 3 m or more (57 using 5 m or more, max = 50 m) between differently coloured traps. Conversely, the other half of the studies (78 out of 163) set differently coloured traps less than 3 m apart from each other, with 48 studies using distances of less than 1 m.

#### 3.1.2 Pan size

**FIGURE 2:**
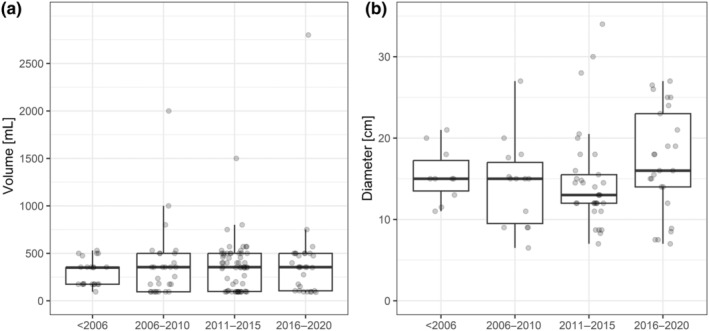
Pan trap (a) volumes (*n* = 139 studies) and (b) diameters (*n* = 82 studies) used in the reviewed literature during different time intervals. Studies were only included when using single pan trap sizes. Shown are box‐ and whisker plots with each data point (grey) representing a single study. Points are jittered on the *x*‐axis for better visibility. For multi‐year studies, the first study year was used.

In total, 142 studies provided single values for pan trap volumes, the average volume being 358.1 ± 27.5 mL (mean ± SE, range: 88.7–2800.0 mL, Figure 2a; 139 studies reporting the first sampling year). A total of 88 studies provided exact single pan trap diameter values, the average diameter being 15.5 ± 0.6 cm (mean ± SE, range: 6.5–34.0 cm, Figure 2b; 82 studies reporting unequivocal details for the first sampling year).

#### 3.1.3 Trapping liquid

Of the 259 studies that provided information on trap liquid composition with various levels of detail, two used propylene glycol without water, while all others used watery solution as basis. Out of the 259 studies only 63 described surfactant details (most often brand names and manufacturer of commercial products). The surfactant: solvent ratio was often described vaguely. Scent details were described as a property of the surfactant in 50 studies, including 41 studies mentioning the use of unscented surfactant. Further components of the trap solution included preservatives (sodium benzoate, ethylene or propylene glycol, sodium chloride and formaldehyde) or other attractants (honey), which were used in a few studies (12 studies in total).

The authors of 86 studies provided explicit figures or sufficient information to derive the amount of trap liquid relative to the total volume of the pan trap. In half of these studies (43 out of 86), traps were filled between 50% and less than 75% of the total pan trap volume. In 35 studies, traps were filled 75% of the volumes or more, while nine studies used traps filled less than 50% (two of these using propylene glycol as trap liquid; a single study used both, traps filled 50% and below 50%, respectively).

#### 3.1.4 Trap height

In total, 198 studies provided information about pan trap height above ground level. The majority of these studies (142 out of 198) used elevated pan traps, while 56 studies placed pan traps on the ground. Overall, 71 studies adjusted pan trap elevation to the surrounding vegetation and/or flower stratum.

#### 3.1.5 Sampling duration

**FIGURE 3:**
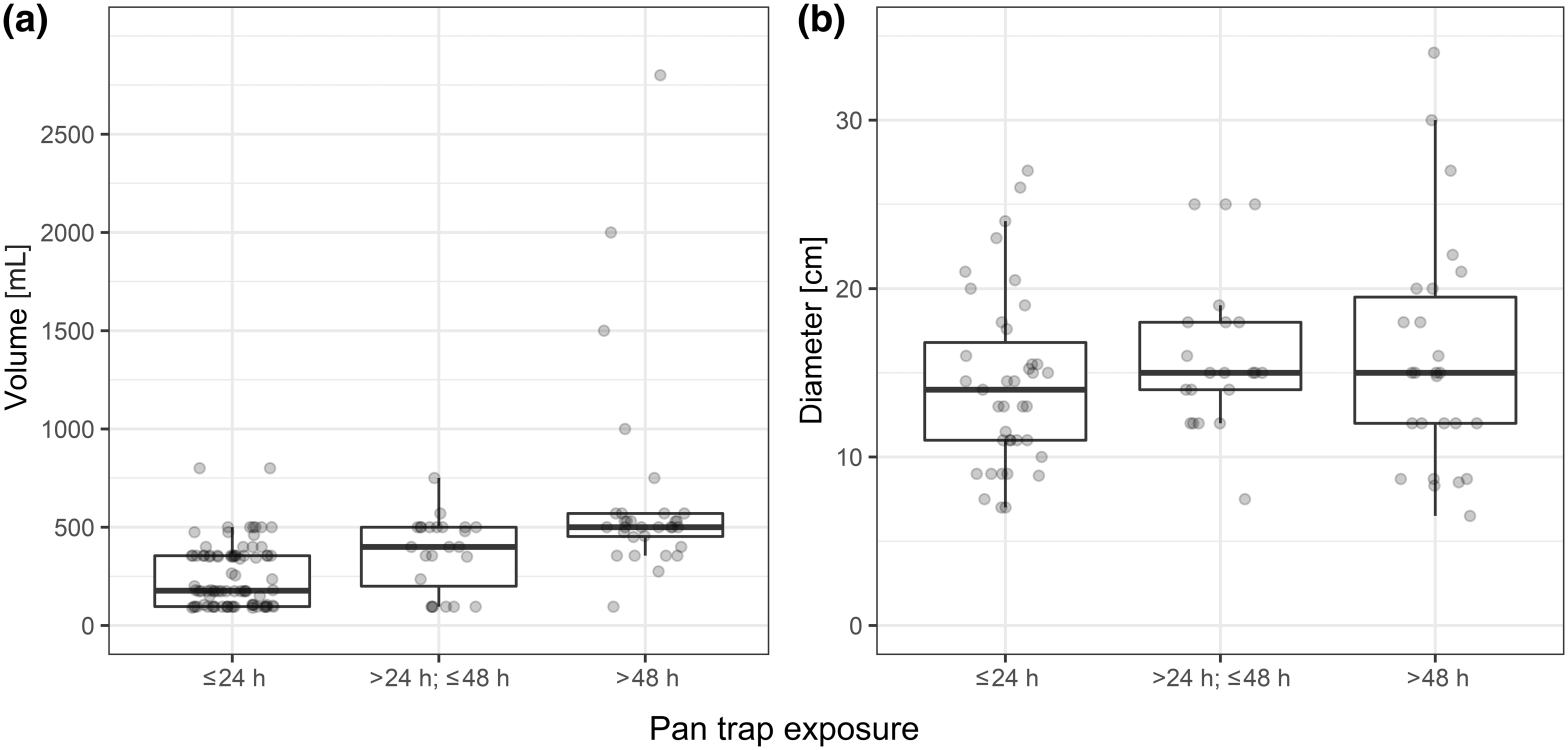
Pan trap volume (*n* = 141 studies) and diameter (*n* = 86 studies) in relation to pan trap exposure time. Shown are box‐ and whisker plots with each data point (grey) representing a single study. Only studies using single pan trap sizes are shown. Points are jittered on the *x*‐axis for better visibility.

A total of 280 studies provided information on the duration of sampling events that could be assigned to a single category and was 24 h or less in 162 studies, while 53 studies used sampling events of more than 24 h up to 48 h, and 65 studies sampled longer than 48 h. Studies that exposed their traps longer, also tended to use larger trap volumes, but not larger trap diameters (Figure 3).

### 3.2 Floral survey methodologies

We identified 109 studies that sampled floral data in addition to bee data. The 35 studies that used floral data as an explanatory variable for the variance of bee abundance and/or bee diversity in pan traps, did not follow a standardised protocol for floral data collection (Table 1). Studies varied in their temporal and spatial relation between floral surveys and bee sampling, in the definition of the target flora and of floral units, in their observation area used for the survey, in the abundance measure (quantitative, semi‐quantitative, presence‐absence) and the processing of raw data (including data pooling and transformation of raw data prior to analysis). Ranges in floral abundance and floral richness covered varied markedly across studies. Although an important parameter, the minority of studies reported adaptation of pan trap height to the surrounding flower and/or vegetation level (Table 1).

**TABLE 1:** Correlations between diversity as well as abundance of bees sampled in pan traps and flowers around pan traps, as observed in the Web of Science listed literature.

References	Correlation: Bee diversity &		Correlation: Bee abundance &		Methods								
	floral diversity	floral abundance	floral diversity	floral abundance	Distance	Timing	Floral units	Observation area	Abundance measure	Pooling	Assessed floral abundance range	Assessed floral richness range	Height adaptation
Abbate et al. (2019)	–	–	–	None^1^				Square ≤ 2 × 2 m	Quantitative	None		–	
Adedoja et al. (2019)	–	–	–	Negative^1^				Square ≤ 2 × 2 m	Quantitative	Pooled	0; >600 [flowers]/10 m^2^	–	Adapted
Bergholz et al. (2022)	None^2^	–	None^2^	–	≤5 m	Simultaneous		Radius ≤ 5 m	–	None	–	1; 22 [species/5 m‐radius]	
Bukovinszky et al. (2017)	–	None^1^	–	–		Longer	Applied	Transect (100 m^2^)	Quantitative	Pooled		–	Adapted
Carper et al. (2014)	None^1^ & positive^1^	–	None^1^ & positive^1^	None^1^			Applied	Area > 2 × 2 m	Quantitative	Pooled		3; 14 [species/plot]	
Classen et al. (2015)	–	–	–	Positive^1^	≤50 m		Applied	Area > 2 × 2 m	Quantitative	Pooled	0; >60,000 [flowers/0.25 ha]	–	
Geslin et al. (2016)	None^1,3^	None^1,3^	None^1,3^	None^1,3^	>50 m			Area > 2 × 2 m	Presence/absence	Pooled	29; 480 [summed index/50 m^2^]	9; 69 [species/50 m^2^]	
Griffiths‐Lee et al. (2022)	None^1^	–	None^4,6^	–		Simultaneous		Area > 2 × 2 m	–	None	–		
Kehinde et al. (2018)^a^	–	None^2^	–	Negative^2^ & positive^2^		≤14 days	Applied	Square ≤ 2 × 2 m	Semi‐quantitative	Pooled	0; 5 [abundance class]	–	
Kovács‐Hostyánszki et al. (2013)	–	None^2,4,5^	–	None^2,4,5^	>50 m	≤14 days		Transect (200 m)	Quantitative	Pooled		–	
Kovács‐Hostyánszki et al. (2021)	–	None^4,6^	–	None^4,6^ & negative^7^		Longer	Applied	Square ≤ 2 × 2 m	Quantitative	Pooled		–	
Larkin and Stanley (2021)	None^4^	None^4^	None4	None^4^		Longer	Applied	Square ≤ 2 × 2 m	Quantitative	Pooled	<500; >2000 [flowers/transect]	10; 39 [species/transect]	Adapted
Lozada‐Gobilard et al. (2021)	–	None^11^ & positive^1,6^	–	None^6^ & positive^1,11^		Simultaneous		Area > 2 × 2 m	Semi‐quantitative	Pooled	0; ≥50 [%]	–	
Lucas et al. (2017)	–	–	–	None^4^, negative^8^, positive^4,8^			Applied	Square ≤ 2 × 2 m	Quantitative	Pooled	4.5; 334.5 [flowers/m^2^]	–	Adapted
Milam et al. (2018)	–	–	None^2^	None^2^				Transect (100 m)	Semi‐quantitative	Pooled	1; 10 [rank]	16; 33 [species/transect]	
Morandin and Kremen (2013)	–	–	–	None^7^ & negative^2^	≤50 m			Square ≤ 2 × 2 m	Semi‐quantitative	None		–	
Morrison et al. (2017)	Positive^2^	–	None^2^	–	≤5 m	Simultaneous	Applied	Square ≤ 2 × 2 m	–	Pooled	–	4; 27 [species/m^2^]	Adapted
Nelson et al. (2021)	Positive^1^	None^1^	Positive^1^	Negative^1^	≤50 m			Square ≤ 2 × 2 m	Quantitative	Pooled	0; >4000 [flowers/m^2^]	3; >15 [species/m^2^]	
O'Connor et al. (2019)^a^	–	–	–	None^4^ & negative^4,6^	≤50 m			Square ≤ 2 × 2 m	Quantitative	Pooled	<7.4; >148 & <54.6; >665 [flowers/m^2^]	–	Adapted
O'Connor et al. (2019)^a^	–	None^5^ & positive^7^	–	None^4,6^	>50 m	Simultaneous	Applied	Transect (200 m)	Semi‐quantitative	None	1; 20,000 [μg sugar per m^2^ per hour]	–	Adapted
Olynyk et al. (2021)	–	–	None^1,3,6,11,19^ & positive^3,6^	None^1,3,6,11,19^, negative^1,11^, positive^6^	≤5 m	Simultaneous		Radius ≤ 5 m	Quantitative	None	0; 1 [indexed number of flowering stems]	1; 15 [species/radius]	
Pardee and Philpott (2014)	–	None^1,3,9^	–	None^1,3,9^	≤50 m	≤14 days		Square ≤ 2 × 2 m	Quantitative	Pooled	11; 1070 [flowers/25 m radius]/0; 370 [flowers/4 m^2^]/0; 8161 [cm^3^ floral volume/4 m^2^]	–	
Pei et al. (2022)	None^1^	None^1^	Negative^1^	Negative^1^		≤14 days		Transect (100 m)	Quantitative	Pooled	0.77 ± 0.18 [mean & 95% CI; flowering stem density/3000 m^2^]	0.0053 ± 0.00070 [mean & 95% CI; species/3000 m^2^]	Adapted
Proesmans et al. (2019)	None^b^ ^,1^	None^b^ ^,1^	None^b^ ^,1^	None^b^ ^,1^	>50 m	Longer		Transect (200 m)	Semi‐quantitative	Pooled			
Rhoades et al. (2017)	None^1^	–	–	–	≤50 m	Simultaneous		Radius > 5 m	–	Pooled	–		
Roberts et al. (2017)	None^b^ ^,1^ & positive^1^	None^b^ ^,1^ & positive^1^	None^b^ ^,1,3,6,9,10,12,13,14,15,16,17,18^; negative^11,13,18^; positive^1,3,6,9,10,11,12;14,15^	None^b^ ^,1,3,6,9,10,12,13,14,15,16,17,18^; negative^11,13,18^; positive^1,3,6,9,10,11,12,14,15^	≤5 m	Simultaneous		Radius ≤ 5 m	Quantitative	Pooled			
Rodríguez and Kouki (2015)	–	None^1^	–	None^4,6^ & positive^4^	≤50 m	≤14 days		Square ≤ 2 × 2 m	Quantitative	None	5.1 ± 2.5; 78.7 ± 66.16 [mean ± SD, flowers/m^2^]	–	
Samnegård et al. (2015)	None^2^	–	–	None^2^	>50 m			Area > 2 × 2 m	Semi‐quantitative	Pooled			
Templ et al. (2019)	–	None^1^	–	None^1^		Simultaneous		Square ≤ 2 × 2 m	Quantitative	Pooled		–	Adapted
Torné‐Noguera et al. (2016)	–	None^2^	–	Positive^c^ ^,2^	≤50 m			Transect (40 m)	Quantitative	Pooled		–	
Turo et al. (2021)	None^1^	None^1^	None^1,3,8,9^	None^1,3,8,9^	≤50 m	Longer	Applied	Square ≤ 2 × 2 m	Quantitative	None	0; >6000 (bloom area)		
Westerberg et al. (2021)	–	–	–	Negative^11^	≤50 m	≤14 days		Square ≤ 2 × 2 m	Presence/absence	None	<0.024; >42 (odds of flower per m^2^)	–	Adapted
Wood et al. (2015)	–	–	–	Positive^4^		Longer	Applied	Transect (variable length)	Semi‐quantitative	None	<1; >100 (flowering units per m^2^)	–	
Zou et al. (2017)	–	–	–	Positive^7^		≤14 days		Square ≤ 2 × 2 m	Presence/absence	Pooled		–	

*Note*: The varying correlations found in different studies are accompanied by differences in methods: distance (between pan traps and floral observations); timing (of floral surveys with regard to bee sampling); floral units; observation area (used in the floral survey); abundance measure (for floral data); pooling (of bee and/or floral data across multiple sampling events, i.e., over time); assessed range for floral abundance and richness; height adaption (of pan traps to flower/ vegetation level). Bee groups analysed: ^1^all bee taxa, ^2^without *Apis* spp., ^3^ground nesting bees, ^4^bumble bees, ^5^wild bees excl. bumble bees, ^6^solitary bees, ^7^ *Apis* spp., ^8^ *Lasioglossum* spp., ^9^cavity nesting bees, ^10^polylectic bees, ^11^eusocial bees, ^12^pith‐nesting bees, ^13^small bees, ^14^medium sized bees, ^15^large bees, ^16^oligolectic bees, ^17^parasitic bees, ^18^soft wood nesting bees, ^19^above‐ground nesting bees.

Abbreviation: –, not applicable.

^a^ Reference comprising two separate studies.

^b^ Composite floral index accounting for floral abundance and diversity.

^c^ Direction of correlation explicit for one target plant species (only marginally significant correlation); direction of correlation for two other plant species (significant correlation) inferred from results and discussion context.

#### 3.2.1 Temporal and spatial gaps between floral and bee sampling

Very few of the 35 studies give any valuable information on the time span between floral survey and bee sampling. Time periods ranged from zero days (simultaneous sampling, Table 1) up to a year (Bukovinszky et al., 2017); non‐simultaneous flower sampling was generally conducted within 14 days (Table 1) or approximately within a month before/after bee sampling (Kovács‐Hostyánszki et al., 2021; Larkin & Stanley, 2021; Proesmans et al., 2019; Turo et al., 2021; Wood et al., 2015). A minority of studies (4 out of 19) focussed their floral samplings close to (≤5 m) pan traps.

#### 3.2.2 A priori selection of sampled flora

Several studies restricted their flower sampling to a particular group of plant species, for example entomophilous flowering plants (Geslin et al., 2016; Proesmans et al., 2019; Rhoades et al., 2017; Templ et al., 2019), and bee‐supporting plants (Milam et al., 2018). In some cases, flower surveys were restricted – based on taxonomy and growth form – to only herbaceous flowering plants (Kovács‐Hostyánszki et al., 2013), forbs (Bergholz et al., 2022), herbs, shrubs and trees (Samnegård et al., 2015), dicotyledonous species (Wood et al., 2015), or to plant species excluding grasses, sedges and rushes (Lucas et al., 2017). Likewise, Olynyk et al. (2021) excluded exclusively wind‐pollinated plant species from the dataset prior to analysis. In some cases, floral surveys restricted the number of plant species due to their flower abundance at the time. Torné‐Noguera et al. (2016) focussed on the six main flowering species, which accounted for more than 70% of the flowers present, while O'Connor et al. (2019) included at least five most common flowering plant species per transect section. In addition, O'Connor et al. (2019), in another survey, and Zou et al. (2017) focussed on crops (apple, strawberry, field beans and oilseed rape).

#### 3.2.3 Sampling replication and raw data

Nearly half of the studies (17 out of 35) used replicate squares of up to 4 m^2^ for flower cover assessment. Few studies used radii around pan traps or assessed flower cover along transects without placing rectangles (Table 1).

Many studies quantitatively assessed floral abundance on a continuous scale in the field (Table 1) by counting (open) flowers or floral units per area (e.g., Larkin & Stanley, 2021; Wood et al., 2015), flowering stems (Olynyk et al., 2021; Pei et al., 2022), or by extrapolating from subsamples to a larger population (Carper et al., 2014; Nelson et al., 2021). Some of these studies converted the number of flowers or floral units per area into estimates of flower cover using average inflorescence size (Bukovinszky et al., 2017; Kovács‐Hostyánszki et al., 2021; Turo et al., 2021). As an alternative option to continuous data, some studies estimated flowers/floral units or floral cover semi‐quantitatively, that is, on an ordinal 2–7 point scale (Table 1). These values were sometimes converted into a quantitative (continuous‐scale) measure before analysis (Griffiths‐Lee et al., 2022; Morandin & Kremen, 2013; Samnegård et al., 2015; Wood et al., 2015). This is also true for O'Connor et al. (2019), who converted ordinal‐scale floral estimates into an estimate of nectar sugar availability, using per‐day values for nectar production, following Baude et al. (2016).

In addition to continuous and ordinal data, presence/absence of flowers were the primary data in some studies. Such data were generally processed prior to the analysis. Geslin et al. (2016) derived an index of abundance from multiple grid cells based on presence/absence of flowering plants in these replicated grid cells. Westerberg et al. (2021) and Zou et al. (2017) assessed presence/absence of flowers per area from photos of sampling squares. While Zou et al. (2017) used the number of grid cells with flowers as a proxy for flower cover, Westerberg et al. (2021) calculated the odds for any flower or for flowers of a particular colour occurring in a sampling square.

#### 3.2.4 Usage of flower units

Several studies used a floral‐unit approach, that is, mainly considering composite flowers (Asteraceae) and umbels from umbellifers (Apiaceae) as one unit instead of multiple flowers (Table 2). Rather than on plant taxonomy, some studies base their definition of a floral unit on insect behaviour. In these studies, a floral cluster was defined as one unit that a pollinator (Classen et al., 2015) or a bee (Carvalheiro et al., 2008) of about 1 cm length, an average‐sized insect (Morrison et al., 2017) or flower visitor (Grass et al., 2016), or a medium‐sized bee (Dicks et al., 2002) would exploit by walking rather than by flying. Some of these behaviour‐based definitions ultimately rely on Saville (1993), who defined blossom units as inflorescences between which bumblebees usually fly. As another approach for simplifying fieldwork, flower‐bearing branches (Carper et al., 2014) and stems (O'Connor et al., 2019) were used as units of observation for calculating the number of flowers, based on the number of flowers per branch or stem, respectively.

**TABLE 2:** Usage of floral units by studies investigating correlations between bee samples in pan traps and floral context, showing inflorescence types, relevant plant taxa and references referred to.

Study	Heads	Racemes	Umbels	Flower stalks	Spikes	Capitula	Single flowers	References
Bukovinszky et al. (2017)	Asteraceae	Fabaceae	Apiaceae	Lamiaceae			Malvaceae, Rosaceae	Rundlöf et al. (2014)
Carper et al. (2014)	Asteraceae							–
Classen et al. (2015)	Asteraceae (e.g.)						Rosaceae (e.g.)	Carvalheiro et al. (2008), Saville (1993)
Kehinde et al. (2018)	*Trifolium repens* (e.g.)		*Daucus carota* (e.g.)		*Rhinanthus minor* (e.g.)	*Centaurea nigra* (e.g.)		Carvell et al. (2004)
Kovács‐Hostyánszki et al. (2021)	Dipsaceae, Asteraceae	Fabaceae	Apiaceae	Campanulaceae, Lamiaceae, Scrophulariaceae			Malvaceae, Rosaceae	Bukovinszky et al. (2017), Rundlöf et al. (2014)
Larkin and Stanley (2021)								Dicks et al. (2002), Saville (1993)
Lucas et al. (2017)	*Trifolium* spp.		Apiaceae		*Narthecium ossifragum*, *Rhinanthus minor*, *Calluna vulgaris*, Orchidaceae			Dicks et al. (2002), Saville (1993)
Morrison et al. (2017)								Grass et al. (2016), Dicks et al. (2002), Saville (1993)
O'Connor et al. (2019)	(not specified)		(not specified)		(not specified)			–
Turo et al. (2021)			*Achillea millefolium*, *Daucus carota*		*Plantago major*, *Plantago lanceolata*		*Cichorium intybus*, Fabaceae	–
Wood et al. (2015)	*Trifolium pratense* (e.g.)		*Daucus carota* (e.g.)		*Rhinanthus minor* (e.g.)	*Centaurea nigra* (e.g.)	(not specified)	Carvell et al. (2007)

### 3.3 Relationship between bee samples and floral environment

Overall, 35 studies included floral data generated from local surveys in their analyses of bee abundance and/or diversity derived from pan trapping (Table 1). No study found a negative correlation between sampled bee diversity and floral diversity or abundance, while studies with non‐significant correlations exceed the number of studies with significant positive correlations (9 vs. 3 studies for floral diversity; 17 vs. 3 studies for floral abundance; Table 1). Likewise, we registered more studies with bee abundance and floral diversity not being significantly correlated (12 studies) than studies with positive (4) or negative correlations (1) between the two parameters. Most often, studies correlated floral abundance and bee abundance; fewer studies described a negative (11) or positive correlation (10) between the two parameters than no significant correlation (20 studies). Resulting correlations varied not only across all 35 studies, but also within groups of studies applying a similar method or analysing a similar sub‐group of bees (Table 1).

## 4 DISCUSSION

### 4.1 Standardisation of pan traps

In general, on the one hand, we find strong tendencies towards standardisation of certain pan trap properties across studies, such as trap coloration and elevation. These properties may either have been established through repeated adoption of the same methodology, or may be based on robust experimental evidence. On the other hand, several other important properties vary greatly between studies, such as trap volume, trap diameter and sampling duration. While there may be valid reasons to adapt established methodology to study context (e.g., fauna and research question), we identified fundamental research gaps that will help to standardise pan trap methodology across studies. Most importantly, our literature review revealed that many studies lack sufficient details on pan trap methodology, rendering cross‐study comparisons more difficult. In the following, the different aspects of the pan trap methods with potential for standardisation are discussed in detail.

#### 4.1.1 Pan coloration

Coloration is a well‐investigated pan trap characteristic. In general, researchers agree that a combination of yellow, blue and white traps covers taxonomic groups best (as proposed before by e.g., Buffington et al., 2021; Krahner et al., 2021; see Sircom et al., 2018 for a review of earlier findings). Studies using single colours were uncommon with yellow often being the preferred colour in these studies. Yellow has been found the most efficient colour for sampling whole bee communities (Gollan et al., 2011; Krahner et al., 2021; Li et al., 2021), although some studies concluded that other colours provide a better sampling efficiency (Acharya et al., 2021) or that they could not identify a single preferable colour (Joseph et al., 2020; Moreira et al., 2016; Padrón et al., 2021; Saunders & Luck, 2013; Sircom et al., 2018). Besides the obvious benefit of saving resources, reasons for using single colours may include similarity of colours to flowers in the immediate surrounding (Geeraert et al., 2020), maximising sampled bee abundance (Li et al., 2021; Olson et al., 2021) or sampled bee species richness (Sanchez et al., 2019) or both (Sahli et al., 2016). Choosing colours representing floral colours in the sampling region (Westphal et al., 2008) or using the colour that is known to attract a specific taxon may be other reasons to decide on the number of used pan trap colours. For the sake of standardisation of sampling effort via colour attractiveness, pan trap exposure to sunlight needs to be considered in addition to the colours used. This is because light conditions, for example, as a consequence of canopy structure casting a shadow over pan traps, may influence the capture rate of different colours for hymenopterans (Abrahamczyk et al., 2010).

Fluorescent colours, that is, those enhanced in reflection by emission of visible light following absorbance of UV light, were integrated into bee sampling a long time ago (e.g., 2001 by Shapiro et al., 2014; 2002 by Roulston et al., 2007). The Bee Inventory Plot (LeBuhn et al., 2003), an early approach to standardised bee sampling methods, mentioned fluorescence explicitly. Despite that, authors continued to describe colour specifications with regard to UV light behaviour imprecisely. Accurately distinguishing between UV‐reflective and fluorescent colours in future studies is essential, since pigments reflecting UV light exist and may be relevant for attracting bees (Koethe et al., 2018). Over the past two decades, UV‐reflective and/or fluorescent colours served as pan trap colours in roughly half of the studies (Figure 1). Robust experimental evidence for the effect of UV‐fluorescence and/or emission on bee sampling results is still lacking, despite indications for an increase in bee attraction through such colours (Droege, 2005; Stephen & Rao, 2005; but see Prendergast et al., 2020). All future studies should state (and validate) details on spectral reflectance in UV and/or visible wavelength ranges of pan colours as a standard when conducting pan trap sampling. It is also important to track potential changes in spectral reflectance due to the ageing process of colour pigments through repeated use of pan traps over one or more sampling seasons.

Despite early advice that coloration of the inner and upper trap surface is sufficient (LeBuhn et al., 2003), researchers continued using coloration on the outside of the trap. When using spray colours, coloration of both in‐ and outside ensures comparability with unsprayed pans made of coloured plastics, and avoids the need to define the basic colour of the unsprayed side of the pan. However, there are also some arguments in favour of dual coloration. Using inconspicuous colours on the exterior trap surface (Davis et al., 2018; Perillo et al., 2017) may decrease long‐range insect attraction (for which pan trap studies often receive criticism). Thus, such camouflaging potentially helps to focus sampling effort on the trap‐surrounding habitat, generating more habitat‐specific data, as has been shown for hoverflies (Laubertie et al., 2006). Given the lack of knowledge regarding bees, we encourage future studies to experimentally address the effect of camouflaging on bee sampling results.

Set‐ups of pan traps with multiple colours improve capture rates by separating individual traps by a distance of at least 3 m. This approach prevents competition for bees between differently coloured traps (Droege et al., 2010), but also involves more effort for installing pan traps compared to more clustered traps within a set. In about half of the studies that share details on spatial separation of traps within colour sets, researchers increased spatial sampling effort with a given number of pan traps, rather than clustered pan traps of different colours close to each other. Clustering is more convenient, especially when deploying elevated pan traps attached to a single rather than multiple poles per set. In 80 of the here‐reviewed studies, trap separation distance within a set (or the lack of it) remained unmentioned, thus evading our analysis, which was solely based on explicit information. We advocate for well‐separated pan traps as the standard approach, where this is feasible, and prefer them to clustered arrangements (e.g., attaching several pans to the same post), as long as additional research and experimental evidence does not relativise the findings by Droege et al. (2010) on the matter.

#### 4.1.2 Pan size

Variation of pan trap diameter and volume has been reviewed before (Gonzalez et al., 2020), and we found similar mean values for both parameters. Ranges for trap diameter and volume were larger in our review than the ones presented by Gonzalez et al. (2020). This comes as no surprise considering the larger number of studies reviewed here. Despite several attempts to standardise pan trap size (Carvell et al., 2016; Droege et al., 2016; LeBuhn et al., 2003), bee researchers continued using various pan trap diameters and volumes. Nevertheless, these three publications resonated in the research community: before 2006, researchers used 180 mL volumes more frequently (as suggested by LeBuhn et al., 2003) than for example 100 mL volumes (recommended by Carvell et al., 2016 and Droege et al., 2016), which became more popular in the last decade (Figure 2). Volumes of the majority of studies fell into three classes (either 100 mL, 350 mL or 500 mL). Such persisting variation in volumes and diameters highlights the still unresolved question of how pan trap size influences bee sampling results. It may also mirror sampling approaches getting adapted to study requirements (e.g., national park authorities may restrict invasive sampling to minimise disturbance, thus pan size and sampling duration).

Investigations into the effect of pan trap size on bee sampling remain insufficient to reach a consensus. While some studies found a positive correlation between trap size and the number of sampled bees (Wilson et al., 2016), others did not (Droege, 2005; Gonzalez et al., 2020). Nevertheless, size information is essential to standardise capture rates among studies. Trap (upper) diameter should be reported rather than only volume, since wider pans expose colour stimuli more than narrow ones with the same volume. Large bees may escape more easily from smaller pan traps (Krahner et al., 2021; Ropars et al., 2020; Rothwell & Ginsberg, 2019) compared to small bees, but this assumption lacks evidence (Gonzalez et al., 2020; Wilson et al., 2016). As long as further evidence is lacking regarding trap size effects on the sampled bee community, we advocate for using larger diameters (i.e., ≥15 cm) and volumes (i.e., ≥500 mL) to maximise sampling efficacy.

#### 4.1.3 Trapping liquid

The additive to lower the surface tension (including composition and quantity) is the most important aspect of the trap liquid, although other factors (e.g., preservative, toxicity and rate of evaporation) may be worth to consider (see Deville & Wheeler, 1998). The lowered surface tension of aqueous trapping solutions prevents bees from escaping. Despite suspected effects of surfactant chemistry and quantity, the reviewed studies rarely gave sufficient detail to make results reproducible (but see Larsen et al., 2014). Manufacturers may change composition of products over time; stating brand names is therefore of limited value for making study results comparable in the long term. Using pure chemical substances would solve this problem, but would increase costs and is uncommon (but see Prendergast et al., 2020). Multiple studies are explicit about potential olfactory stimuli emitted from the trapping liquid, as these may deter (Droege et al., 2016; LeBuhn et al., 2016) or attract different bee species and thus might filter the sampled fauna. Detergents with specific scents related to natural scents were used intentionally in a few studies, for example, lemon scent (Heneberg et al., 2019; Heneberg & Bogusch, 2014, 2020), and apple scent (Larsen et al., 2014). However, no rationale supports the usage of a specific scent in these studies. To the best of our knowledge, the effect of surfactant concentration on the (reduced) escape rate of bees has never been investigated. Future experimental studies on different combinations of (ideally pure) non‐fragrant substances in various concentrations could help to formulate precise methodological setup recommendations. We recommend that researchers are more explicit about what and how much they use in their future works.

Liquid volumes can affect sampling efficacy (Montgomery et al., 2021) and should be adjusted to expected evaporation where possible, yet only a small fraction of studies shares information on the amount of liquid filled into traps (absolute figures or values relative to trap volume). Small pan traps are more prone to drying out than larger traps (Droege et al., 2016; Gonzalez et al., 2020). Across studies, researchers tended to expose greater volume traps longer than smaller volume traps (Figure 3), which might indicate that evaporation was accounted for. Almost half of the studies that provided information on liquid volume filled their traps to 50%–75% of the total volume. This may indicate an optimal trade‐off: while too low volumes may reduce capture rates, filling above 75% involves a high risk of spillover (with loss of specimens) during rainfall. Experimental studies are required to identify effects of liquid volume on bee sampling, for comparisons across studies that do not share a common methodology in this regard.

#### 4.1.4 Trap height

Matching our expectations, the majority of studies used elevated pan traps, and many of these did so to adapt trap height to the floral stratum, that is, to place traps at the height of or slightly above surrounding flowers. Proximity of pan traps to flowers has been suggested to yield samples representative of the flower visiting bee community (Cane et al., 2000), excluding bees not searching for floral rewards (Mpondo et al., 2021). Elevated pan traps collect more bee individuals and species than ground‐level traps (Geroff et al., 2014; McCravy & Ruholl, 2017). Valid reasons for placing pan traps on the ground are surrounding flowers being near or at ground level (Leong & Thorp, 1999), reduction of evaporation and protection of traps against the wind (Cunningham‐Minnick et al., 2020), or camouflaging outside coloration.

Pan trap samples are sometimes suspected to collect individuals in transit rather than bees foraging in the sampled habitat (Hopwood, 2008; Wheelock & O'Neal, 2016). Given that colour stimuli play an important role in long‐distance flower detection by bees (Giurfa et al., 1996; Spaethe et al., 2001), researchers may want to reduce lateral visibility of traps. This is often achieved by setting them up within the surrounding canopy (e.g., Pei et al., 2022; Westphal et al., 2008) and hiding them from side view, which also prevents vandalism, including interference with wildlife (Pei et al., 2022). In contrast, some researchers place pan traps slightly above the vegetation and/or flower stratum (e.g., Buri et al., 2014; Olson et al., 2021), possibly increasing detection of pan traps by foraging bees. While Ballare et al. (2019) removed vegetation only above pan traps to increase trap visibility, Sheffield et al. (2013) used a grey plastic rim around traps to standardise trap visibility throughout the season. Phillips et al. (2019) went even further and flattened the vegetation surrounding their pan traps. For inventories and monitoring programmes that investigate foraging bees associated with a particular habitat, the latter methods may confound sampling results. In these programmes, pan traps that are adapted in height to the surrounding floral stratum became accepted as state of the art.

#### 4.1.5 Sampling duration

Most of the reviewed studies gave trap sampling duration as an approximate value, including “greater‐than” and “less‐than” values. This obscures sampling effort, making inter‐study comparison difficult. Moreover, because more precise information was unusual, we were unable to conduct a more detailed analysis of the relationship between sampling time and pan trap size in this review (Figure 3). Sampling duration was often less than 1 day when pan traps were combined with another sampling method (e.g., sweep‐netting or transect walks; Kwaiser & Hendrix, 2008; Osterman et al., 2021). Studies that simultaneously investigated multiple sites within a region often sampled for about 1 day (e.g., Acharya et al., 2021; McKechnie et al., 2017). When a limited number of staff members had to sample larger areas, sampling duration was usually extended to two or more days (e.g., Joseph et al., 2020; Morandin & Winston, 2005). Some studies extended the duration of some sampling events when they had to deal with unfavourable conditions such as cloud cover, rain or wind to retain the number of days with favourable conditions in all sampling sites (e.g., Morandin & Winston, 2005; Pfiffner et al., 2018). Such extension of sampling should be used with care since further sources of variation (evaporation and temporal species turnover) are introduced in an unbalanced manner, likely to affect bee community composition (Tuell & Isaacs, 2010). LeCroy et al., 2020 found a linear relationship between bowl days (number of pan traps*sampling days) and the number of sampled individuals. However, the relation between sampling duration and the number of sampled individuals lacks experimental evidence, and researchers should aim for sampling durations and conditions as equal as possible across sampling sites.

### 4.2 Floral survey methodologies

#### 4.2.1 Temporal and spatial gaps between floral and bee sampling

While floral vegetation should ideally be sampled on the same day as bees (simultaneous sampling), manageable workload may enforce a shift of multiple days (non‐simultaneous sampling). The majority of non‐simultaneous floral samplings took place within 14 days before/after bee sampling (Table 1), with the exception of one study sampling with a 1 year gap (Bukovinszky et al., 2017). Extended periods between pan trap and floral resource sampling are likely to confound results on the relationship between sampled flora and bee fauna, since both floral composition and the composition of bee communities may substantially change over the course of a few weeks. Adult females of many solitary bee species have a life span of 4–6 weeks (Schindler et al., 2013), and flowering time of a local plant population may be within the same order of magnitude (Herrera, 1986). For accurate floral data, a gap of 7 days or less between sampling events seems reasonable, while for bees with a life span of 4–6 weeks, the double amount may be acceptable. Without further experimental proof, researchers should aim for shorter gaps. Likewise, future studies on correlations between bee and flower samples should refrain from pooling floral and/or bee data across temporally replicated samples in order to preserve a possible tight link between floral and bee data.

The spatial context, in which pan traps were embedded in their floral environment, varied markedly among the studies. Distances between pan traps and vegetation samples ranged from less than 1 m (Morrison et al., 2017) to 200 m (Proesmans et al., 2019). Such distances cover the foraging range of less mobile bee species (e.g., Hofmann et al., 2020; Westphal et al., 2006). Thus, all reviewed studies that investigated the relationship between floral resources and bee communities (Table 1) sampled flowers at a bee‐relevant spatial scale. For investigating potential competition between real flowers and pan traps, the appropriate scale for flower sampling may be much smaller than 200 m (see Section 4.3 below).

#### 4.2.2 A priori selection of sampled flora

The trade‐off between the number of simultaneously sampled locations in a landscape and local floral sampling effort affects the choice of floral assessment method and may restrict or simplify study designs. Focussing floral assessment on a (sub) set of plant species is a common way to save resources in fieldwork. All subsetting to entomophily (entomophilous and bee‐supporting flowering plants) can be considered ecologically justified, but may not account for some relevant food plants for bees (such as *Quercus* spp., e.g., Radmacher & Strohm, 2010). Focussing on the dominant flowering species (e.g., O'Connor et al., 2019) may not account for resource use in pollen‐specialist bees, thus potentially obscures the relationship between assessed floral resources and part of the sampled bee community. Floral resources from trees were assessed in a minority of studies (e.g., Carper et al., 2014; Classen et al., 2015; Samnegård et al., 2015). In addition to the herbaceous and shrubby vegetation, trees as a mass‐flowering crop may significantly improve the assessment of relevant floral resources and may be worth the additional effort.

#### 4.2.3 Sampling replication and raw data

Assessing the number of flowers or flower units per area, usually within replicated quadrats (relevés; e.g. Nelson et al., 2021; Pardee & Philpott, 2014), is the most common method among studies investigating potential correlations between floral and bee samples. Counting flower units is time consuming, and researchers often preferred time‐saving approaches, such as flower cover estimation (e.g., Morandin & Kremen, 2013; Zou et al., 2017). Flower cover as a proxy for resource availability (Potts et al., 2003; Torresani et al., 2023) is a useful alternative. Composite indices that summarise abundance and diversity information in a single figure (e.g., Proesmans et al., 2019; Roberts et al., 2017) rendered comparison with other studies virtually impossible. The relatively novel approach of using presence/absence of plant species as primary data on floral abundance (Geslin et al., 2016; Westerberg et al., 2021; Zou et al., 2017) has a great potential for reducing fieldwork and should be investigated further.

Researchers frequently processed floral raw data. Introducing pseudo‐precision in statistical models by converting primary data sampled on an ordinal point scale into numbers of flowers or flowering units (Samnegård et al., 2015; Wood et al., 2015) or into percent cover (Morandin & Kremen, 2013) saves time in the field but is a questionable approach. Subsequent analysis should therefore incorporate ordinal classes rather than floral variables on a metric scale. More generally, conversions and extrapolations concealed raw data and hindered cross‐comparisons between studies. We advocate for using unprocessed floral sampling data whenever possible, or providing them as a supporting information.

#### 4.2.4 Usage of flower units

Floral units were counted in almost one‐third of the studies considered, although the definition of units varied. Those definitions involving behaviour (flying and walking) have the drawback of being species‐specific (energy consumption and time budget), reducing reproducibility. The categorisation of flowers into units is primarily not only a timesaving approach, but also reflects how wild bees perceive their floral environment (functional units). Researchers have to be explicit about the taxonomic group (generally plant family; e.g., Apiaceae) and structure (e.g., umbel) that they consider a flower unit (see Table 2). Some researchers speeded up floral surveys by sampling larger units of observation, such as branches and stems. Future comparative experimental studies should investigate such simplifications of floral resource data and if they can always reliably reveal potential links between bees and flowers at the habitat scale.

### 4.3 Relationship between bee samples and floral environment

The numbers of flowers surrounding the pan traps are often thought to significantly affect local pan trapping results. It was therefore surprising that only a minority of all here‐reviewed studies included an investigation of the effect of floral availability on sampled bee communities. We were also surprised that these studies did not support a consistent effect of floral abundance and richness on bee abundance and richness. Most studies reported a lack of correlation between bee abundance/diversity and flower abundance/diversity. Studies with significant positive or negative correlations do not have obvious characteristics in common (Table 1, Table S2). We suspect that some of the reviewed papers may have dismissed floral data after pre‐analyses due to non‐significant results. For our review and for comparison in general, it would be favourable that such non‐significant correlation results were more generally considered of importance and publishable.

Competition between pan trap units levels off at a distance of about 3–5 m (Droege et al., 2010), which may also apply for competition between flowers and pan traps. Surprisingly most of the here reviewed studies (all but four) investigated the link between floral resources and bee communities at a spatial scale of more than 5 m (Table 1). While sampling farther away from pan traps may be a suitable approach in habitats where floral resources occur homogeneously at small and large scales, we endorse future studies to collect floral data at a spatial scale of a few meters when assessing potential competition between pan traps and flowers.

The lack of evidence and the ambiguous results even within single studies may result from the apparent disagreement on standardisation of floral survey methodology (and to a lesser extent of pan trap methods; Table 1 and Table S2). Up to now, we do not understand if or how floral resources may compete with pan traps for sampled bees or facilitate sampling with pan traps. Due to the many discrepancies in sampling methodologies, we still lack evidence that the incorporation of floral environment data into analyses adequately addresses a potential bias in pan trapping results (Potts et al., 2021). Such evidence could be provided by future multi‐year experiments, which make use of standardised vegetation and pan trapping methodologies, while manipulating floral cover or using an existing range of floral cover (ideally from 0% to 100%) and considering recommendations made earlier.

## 5 CONCLUSION

Despite the long tradition of pan trapping bees, multiple aspects of this method remain non‐standardised. Some factors have not been systematically investigated so far (e.g., trap liquid, camouflaging, size, and shape), and should be addressed in the future. For some parameters, a variety of methods is used despite existing examples of best practice (e.g., with regard to pan trap colours, distance between differently coloured pan traps, elevation of pan traps). Researchers should consider previously published results in their sampling designs, if feasible, in order to increase comparability of pan trap studies for bees. More generally, the present work shows that methods should be reviewed regularly, especially when their use increases.

We encourage bee researchers to adopt the general consensus of using multi‐colour (yellow, white and blue) fluorescent pan traps, with single traps separated by 3–5 m in order to increase sampling effort. Pan traps should be raised to the immediate flower stratum, without projecting from it, to target bees foraging at the sampling site and to avoid attraction of bees over longer distances. For increasing sampling efficacy, we suggest using traps not less than 500 mL in volume and minimally 15 cm in diameter, filled between 50% and 75%. When using water, unscented detergent should be used to lower surface tension, in order to avoid deterrence or attraction of specific bee taxa via olfactory stimuli.

How surrounding flowers influence pan trap sampling of bees remains an open question. One important step towards resolving this issue is to identify floral assessment methods that provide floral data at an appropriate resolution and scale. Instead of repeatedly reinventing the wheel, future studies should rely on established methods for floral assessment in a standardised manner, for the sake of comparability. In order to track changes in bee and plant flowering phenology, researchers should target a maximum gap between bee and flower sampling of 7 days or, if unavoidable, up to 2 weeks if the flora allows for it. Researchers should survey flowers within a radius of 5 m around the pan traps or, when surveying at larger distances from the trap, ensure that floral resources at sampling points matches those within the 5 m radius around pan traps. Until we understand the mechanisms underlying the correlations, it may be best practice not to use vegetation data in statistical models to correct pan captures.

Pan traps play an important role in current and future long‐term bee monitoring programmes (Carvell et al., 2016; Droege et al., 2016; Potts et al., 2021), and so there is a need for methodological standardisation, and a strong urge for the research required for it. This need for further standardisation may not have been appreciated over the years due to long‐standing assumptions about the way floral environments bias pan trap results (e.g., Prendergast & Hogendoorn, 2021; Wilson et al., 2008). However, we see a great reward of the expected findings in the long term, as they may fundamentally improve our perception of bee communities. This entails positive repercussions in many associated research areas.

## AUTHOR CONTRIBUTIONS

**André Krahner:** Conceptualization (lead); formal analysis (lead); methodology (lead); writing – original draft (lead); writing – review and editing (equal). **Anke C. Dietzsch:** Writing – original draft (lead); writing – review and editing (equal). **Tobias Jütte:** Writing – original draft (supporting); writing – review and editing (equal). **Jens Pistorius:** Writing – original draft (supporting); writing – review and editing (equal). **Jeroen Everaars:** Writing – original draft (lead); writing – review and editing (equal).

## CONFLICT OF INTEREST STATEMENT

There was no conflict of interest for the authors in this study.

## Supporting information


Tables S1–S3.

## Data Availability

Relevant data are provided as electronic supplementary material.
