# Supplementary material for: Standardising bee sampling: A systematic review of pan trapping and associated floral surveys
Source: Ecol Evol. 2024 Mar 17;14(3):e11157. doi: 10.1002/ece3.11157 (PMC10944983; doi:10.1002/ece3.11157)
Supplement: Supplementary file 1 — Tables S1–S3. [file ECE3-14-e11157-s001.zip › Table_S1.docx]

**Table S1.** Complete list of studies resulting from the systematic literature research, including classification according to relevance.

| Author | | Relevance | | Remarks | |
| --- | --- | --- | --- | --- | --- |
| Abbate, Campbell, Kimmel, & Kern, 2019 | | Relevant | |  | |
| Abrahamczyk, Steudel, & Kessler, 2010 | | Relevant | |  | |
| Acharya et al., 2021 | | Relevant | |  | |
| Ackermann & Weigend, 2006 | | Not relevant | | no use of pan traps | |
| Adedoja, Kehinde, & Samways, 2021 | | Relevant | |  | |
| Adedoja, Kehinde, & Samways, 2019 | | Relevant | |  | |
| Affek, Regulska, Kolaczkowska, Kowalska, & Affek, 2021 | | Relevant | |  | |
| Aguiar & Ramos, 2020 | | Not relevant | | no use of pan traps | |
| Aizen & Feinsinger, 1994 | | Relevant | |  | |
| M. L. S. Almeida et al., 2020 | | Relevant | |  | |
| R. P. S. Almeida, Arruda, F., V, Silva, & Coelho, 2019 | | Relevant | |  | |
| Amy et al., 2018 | | Relevant | |  | |
| Andersson, Koffman, Sjödin, & Johansson, 2017 | | Relevant | |  | |
| Arnan, Bosch, Comas, Gracia, & Retana, 2011 | | Relevant | |  | |
| Ashcroft, Gollan, & Batley, 2012 | | Relevant | |  | |
| Assis et al., 2021 | | Relevant | |  | |
| Ávila-Gómez, Meléndez-Ramírez, Castellanos, Zuria, & Moreno, 2019 | | Relevant | |  | |
| Bąk-Badowska, 2012 | | Relevant | |  | |
| Ballare et al., 2019 | | Relevant | |  | |
| Banaszak, Motyka, & Szczepko, 2013 | | Relevant | |  | |
| Bartholomew & Prowell, 2005 | | Relevant | |  | |
| Bashir, Saeed, & Sajjad, 2013 | | Relevant | |  | |
| Bashir et al., 2019 | | Relevant | |  | |
| Basque et al., 2019 | | Not relevant | | not a bee study | |
| Basu et al., 2016 | | Relevant | |  | |
| Baum & Wallen, 2011 | | Relevant | |  | |
| Bergholz, Sittel, Ristow, Jeltsch, & Weiss, 2022 | | Relevant | |  | |
| Berglund & Milberg, 2019 | | Relevant | |  | |
| Bessa, Hockings, & Biro, 2021 | | Not relevant | | not a bee study; no use of pan traps | |
| Bhandari, Longing, & West, 2020 | | Relevant | |  | |
| Bhandari et al., 2018 | | Relevant | |  | |
| Biddinger & Joshi, 2017 | | Not relevant | | not a bee study | |
| Boyer, Fragoso, Mabin, & Brunet, 2020 | | Relevant | |  | |
| Braatz et al., 2021 | | Relevant | |  | |
| Breland, Turley, Gibbs, Isaacs, & Brudvig, 2018 | | Relevant | |  | |
| Bried & Dillon, 2012 | | Relevant | |  | |
| Briggs et al., 2022 | | Relevant | |  | |
| Brooks & Nocera, 2020 | | Relevant | |  | |
| Buchanan, Gibbs, Komondy, & Szendrei, 2017 | | Relevant | |  | |
| Buffington et al., 2021 | | Relevant | |  | |
| Bukovinszky et al., 2017 | | Relevant | |  | |
| Buri, Humbert, & Arlettaz, 2014 | | Relevant | |  | |
| Calizaya-Melo, Aguilar, & Lopez Tejeda, 2021 | | Not relevant | | study language is Spanish | |
| J. W. Campbell, Hanula, & Waldrop, 2007 | | Relevant | |  | |
| Joshua W. Campbell, Grodsky, Monroe, & Martin, 2021 | | Relevant | |  | |
| Joshua W. Campbell, Miller, & Martin, 2016 | | Relevant | |  | |
| Joshua W. Campbell, Milne, Dinh, Daniels, & Ellis, 2020 | | Not relevant | | not a bee study | |
| Joshua W. Campbell, Vigueira, Viguiera, & Greenberg, 2018 | | Relevant | |  | |
| Cane, Minckley, & Kervin, 2000 | | Relevant | |  | |
| Cane, Kervin, & Minckley, 2013 | | Not relevant | | no use of pan traps | |
| Carper, Adler, Warren, & Irwin, 2014 | | Relevant | |  | |
| Castro, Tortosa, Jimenez, & Carpio, 2017 | | Relevant | |  | |
| Cavalcante Neto, Steenbergen, Roberto Zamuner, & Tudella, 2021 | | Not relevant | | not a bee study; no use of pan traps | |
| Ceballos et al., 2015 | | Not relevant | | not a bee study | |
| Choate, Hickman, & Moretti, 2018 | | Relevant | |  | |
| Classen et al., 2015 | | Relevant | |  | |
| Cohen, Egerer, Thomas, & Philpott, 2022 | | Relevant | |  | |
| Connelly, Poveda, & Loeb, 2015 | | Relevant | |  | |
| Cook, Birch, Merickel, Lowe, & Page-Dumroese, 2011 | | Not relevant | | no use of pan traps | |
| Cope, Campbell, Grodsky, & Ellis, 2019 | | Relevant | |  | |
| Corbet, Chen, Chang, & Huang, 2020 | | Not relevant | | no use of pan traps | |
| Corbin, Awde, & Richards, 2021 | | Relevant | |  | |
| Costa, Araujo, Fernandes, Silva, & Sales Junior, 2016 | | Not relevant | | study language is Portuguese | |
| Creedy et al., 2020 | | Relevant | |  | |
| M. A. Cruz-Sanchez, Asis, Gonzalez, Tormos, & Gayubo, 2014 | | Not relevant | | not a bee study | |
| Miguel A. Cruz-Sanchez, Asis, Gayubo, Tormos, & Gonzalez, 2011 | | Not relevant | | not a bee study | |
| Cunningham-Minnick, Peters, & Crist, 2020 | | Relevant | |  | |
| Curtis, Bowie, & Hodge, 2019 | | Relevant | |  | |
| Dafni & Kevan, 1997 | | Not relevant | | no use of pan traps | |
| Dafni & Potts, 2004 | | Not relevant | | not a bee study | |
| Davis, Kelly, Maggs, & Stout, 2018 | | Relevant | |  | |
| Decker & Harmon-Threatt, 2019 | | Relevant | |  | |
| Delphia, Griswold, Reese, O'Neill, & Burkle, 2019 | | Relevant | |  | |
| Demirci, Caner-Saltık, Türkmenoğlu, Özçilingir-Akgün, & Bakırer, 2004 | | Not relevant | | not a bee study | |
| Dingha, Omaliko, Amoah, Jackai, & Shrestha, 2021 | | Relevant | |  | |
| Dingha, Jackai, Amoah, & Akotsen-Mensah, 2021 | | Relevant | |  | |
| Dirrigl, Frank J., Jr., 2012 | | Not relevant | | not a bee study | |
| Dogan & Havvatoglu, 2003 | | Not relevant | | not a bee study | |
| Donovall, Leo R., III & vanEngelsdorp, 2010 | | Relevant | |  | |
| Droege et al., 2010 | | Relevant | |  | |
| Dubet da Silva Mouga, Denise Monique & Warkentin, 2016 | | Relevant | |  | |
| Easton & Goulson, 2013 | | Relevant | |  | |
| Echezona & Offordile, 2011 | | Not relevant | | not a bee study | |
| Eckerter, Buse, Bauhus, Förschler, & Klein, 2021 | | Relevant | |  | |
| Elwell, Griswold, & Elle, 2016 | | Relevant | |  | |
| Estienne, Mundry, Kühl, & Boesch, 2017 | | Not relevant | | no use of pan traps | |
| Estienne, Robira, Mundry, Deschner, & Boesch, 2019 | | Not relevant | | no use of pan traps | |
| Estienne, Stephens, & Boesch, 2017 | | Not relevant | | no use of pan traps | |
| Fan et al., 2017 | | Not relevant | | not a bee study | |
| Fisogni et al., 2020 | | Relevant | |  | |
| Forss, Motes-Rodrigo, Hrubesch, & Tennie, 2020 | | Not relevant | | no use of pan traps | |
| Fortel et al., 2014 | | Relevant | |  | |
| Fox et al., 2015 | | Relevant | |  | |
| Frampton, Droege, Conrad, Prager, & Richards, 2008 | | Relevant | |  | |
| Franceschinelli et al., 2019 | | Relevant | |  | |
| Galbraith, Griswold, Price, & Bosque-Pérez, 2020 | | Relevant | |  | |
| Garibaldi et al., 2021 | | Relevant | |  | |
| Garratt et al., 2019 | | Relevant | |  | |
| Geeraert et al., 2020 | | Relevant | |  | |
| Gerner & Sargent, 2022 | | Relevant | |  | |
| Geroff, Gibbs, & McCravy, 2014 | | Relevant | |  | |
| Gervais, Chagnon, & Fournier, 2018 | | Not relevant | | not a bee study | |
| Gervais, Fournier, Sheffield, & Chagnon, 2017 | | Relevant | |  | |
| Geslin, Le Féon, et al., 2016 | | Relevant | |  | |
| Geslin, Le Féon, Kuhlmann, Vaissière, & Dajoz, 2015 | | Relevant | |  | |
| Geslin, Oddie, et al., 2016 | | relevant | |  | |
| Gezon, Wyman, Ascher, Inouye, & Irwin, 2015 | | Relevant | |  | |
| Giles & Ascher, 2006 | | Relevant | |  | |
| Gill & O'Neal, 2015 | | Relevant | |  | |
| Goldstein & Ascher, 2016 | | Relevant | |  | |
| Goldstein & Scott, 2015 | | Not relevant | | no use of pan traps | |
| Gollan, Ashcroft, & Batley, 2011 | | Relevant | |  | |
| Gonçalves, Sydney, Oliveira, & Artmann, 2014 | | Relevant | |  | |
| González, Salvo, & Valladares, 2015 | | Not relevant | | not a bee study | |
| Gonzalez et al., 2020 | | Relevant | |  | |
| Gonzalez, Park, Çakmak, Hranitz, & Barthell, 2016 | | Relevant | |  | |
| Graham, Gibbs, Wilson, May, & Isaacs, 2021 | | Relevant | |  | |
| Griffiths-Lee, Nicholls, & Goulson, 2022 | | Relevant | |  | |
| Gruber, 2016 | | Not relevant | | no use of pan traps | |
| Grundel, Frohnapple, Jean, & Pavlovic, 2011 | | Relevant | |  | |
| Grundel, Jean, et al., 2011 | | Relevant (duplicate) | | same data as in Grundel, Frohnapple, et al. (2011) | |
| Habel & Ulrich, 2021 | | Relevant | |  | |
| Habel & Ulrich, 2020 | | relevant | |  | |
| Haberski, Hagelin, Barger, Sikes, & DuBour, 2021 | | Not relevant | | no use of pan traps | |
| R. Halinski, dos Santos, Kaehler, & Blochtein, 2018 | | Relevant | |  | |
| Rosana Halinski, Dorneles, & Blochtein, 2015 | | Relevant (duplicate) | | same data as in R. Halinski et al. (2018) | |
| H. G. Hall, 2016 | | Relevant (duplicate) | | same data as in H. G. Hall and Ascher (2010), H. G. Hall and Ascher (2011), and H. G. Hall and Ascher (2014) | |
| H. G. Hall & Ascher, 2010 | | Relevant | |  | |
| H. G. Hall & Ascher, 2011 | | Relevant | |  | |
| H. G. Hall & Ascher, 2014 | | Relevant | |  | |
| M. Hall, 2018 | | Not relevant | | no use of pan traps | |
| M. A. Hall & Reboud, 2019 | | Not relevant | | no use of pan traps | |
| Hanula & Horn, 2011 | | Relevant | |  | |
| Hanula, Horn, & O'Brien, 2015 | | Relevant | |  | |
| Happe et al., 2018 | | Relevant | |  | |
| Harris, Braman, & Pennisi, 2017 | | Relevant | |  | |
| Hatten, Looney, Strange, & Bosque-Pérez, 2013 | | Not relevant | | no use of pan traps | |
| Hendrychová & Bogusch, 2016 | | Relevant | |  | |
| Heneberg, Bogusch, Tauchmanová, Řezáč, & Astapenková, 2017 | | Relevant | |  | |
| Heneberg & Bogusch, 2014 | | Relevant | |  | |
| Heneberg & Bogusch, 2020 | | Relevant | |  | |
| Heneberg, Bogusch, & Řezáč, 2017 | | Relevant | |  | |
| Heneberg, Bogusch, & Řezáč, 2019 | | Relevant | |  | |
| Heneberg, Bogusch, & Řezáč, 2016 | | Relevant | |  | |
| Hevia, Carmona, Azcárate, Heredia, & González, 2021 | | Relevant | |  | |
| Hevia et al., 2016 | | Relevant | |  | |
| Hong & Li, 2005 | | Not relevant | | no use of pan traps | |
| Hopwood, 2008 | | Relevant | |  | |
| Howard, Nisal Ratnayake, Dyer, Garcia, & Dorin, 2021 | | Relevant | |  | |
| J. R. Hudson, Hanula, & Horn, 2013 | | Relevant | |  | |
| J. Hudson, Horn, & Hanula, 2020 | | Relevant | |  | |
| Hung, Sandoval, Ascher, & Holway, 2021 | | Relevant | |  | |
| Hutchinson et al., 2022 | | Relevant | |  | |
| Ikemoto & Yokoi, 2021 | | Not relevant | | no use of pan traps | |
| Ikemoto, Kuramitsu, Sueyoshi, Seguchi, & Yokoi, 2021 | | Relevant | |  | |
| Ishay et al., 2006 | | Not relevant | | not a bee study | |
| Jackson, Turner, & Pearson, 2014 | | Relevant | |  | |
| Johansson, Gustafsson, Andersson, & Hylander, 2020 | | Relevant | |  | |
| Johnson, Edwards, & Johnson, 2021 | | Relevant | |  | |
| Joseph, Harris-Shultz, Jespersen, Vermeer, & Julian, 2020 | | Relevant | |  | |
| Joshi, Biddinger, Fleischer, & Passoa, 2013 | | Not relevant | | not a bee study | |
| Joshi et al., 2015 | | Relevant | |  | |
| Kammerer, Tooker, & Grozinger, 2020 | | Not relevant | | no use of pan traps | |
| Kauffman et al., 2021 | | Not relevant | | not a bee study | |
| Kehinde, Wehrden, Samways, Klein, & Brittain, 2018 | | Relevant | |  | |
| Knapp et al., 2022 | | Relevant | |  | |
| Kohler, Sturm, Sheffield, Carlyle, & Manson, 2020 | | Relevant | |  | |
| Kovac & Stabentheiner, 1999 | | Not relevant | | not a bee study | |
| Kovacic, Puskadija, Ozimec, Majic, & Sarajlic, 2016 | | Relevant | |  | |
| Kovács-Hostyánszki et al., 2013 | | Relevant | |  | |
| Kovács-Hostyánszki, Soltész, Szigeti, Somay, & Báldi, 2021 | | Relevant | |  | |
| E. Kozuharova, 2018 | | Not relevant | | no use of pan traps | |
| E. K. Kozuharova, 2018 | | Not relevant | | no use of pan traps | |
| Krahner, Schmidt, Maixner, Porten, & Schmitt, 2021 | | Relevant | |  | |
| Krewenka, Holzschuh, Tscharntke, & Dormann, 2011 | | Relevant | |  | |
| Ksiazek, Fant, & Skogen, 2012 | | Relevant | |  | |
| Kuhlman & Burrows, 2017 | | Relevant | |  | |
| Kumari, Singh, Singh, Bhatia, & Nain, 2019 | | Not relevant | | not a bee study | |
| Kwaiser & Hendrix, 2008 | | Relevant | |  | |
| Ladd, Yates, Dillon, & Palmer, 2019 | | Relevant | |  | |
| Lagucki, Burdine, & McCluney, 2017 | | relevant | |  | |
| Lamprecht, Maierhofer, & Röllig, 2006 | | Not relevant | | no use of pan traps | |
| Lapuente, Hicks, & Linsenmair, 2017 | | Not relevant | | no use of pan traps | |
| Larkin & Stanley, 2021 | | Relevant | |  | |
| Larsen, Minor, Cruickshank, & Robertson, 2014 | | Relevant | |  | |
| Lasway et al., 2021 | | Relevant | |  | |
| Lazarina et al., 2016 | | Relevant | |  | |
| Le Féon et al., 2013 | | Relevant | |  | |
| Le Féon et al., 2016 | | Relevant | |  | |
| LeCroy, Savoy-Burke, Carr, Delaney, & Roulston, 2020 | | Relevant | |  | |
| Leguizamón, Debandi, & Vázquez, 2021 | | Relevant | |  | |
| Leong & Thorp, 1999 | | Relevant | |  | |
| H. Li et al., 2021 | | Relevant | |  | |
| J.-K. Li & Huang, 2009 | | Not relevant | | no use of pan traps | |
| Longcore et al., 2015 | | Not relevant | | no use of pan traps | |
| Lopes & Machado, 1998 | | Not relevant | | no use of pan traps | |
| Lorandi, Mustin, Halinski, & Iserhard, 2023 | | Relevant | |  | |
| Love & Cane, 2016 | | Relevant | |  | |
| Lowenstein, Huseth, & Groves, 2012 | | Relevant | |  | |
| Lozada-Gobilard et al., 2021 | | Relevant | |  | |
| Lucas, Bull, Vere, Neyland, & Forman, 2017 | | Relevant | |  | |
| Lundin, Rundlöf, Smith, & Bommarco, 2012 | | Not relevant | | not a bee study | |
| Maia et al., 2020 | | Relevant | |  | |
| Marshall et al., 2015 | | Relevant | |  | |
| Martínez-Harms et al., 2012 | | Not relevant | | not a bee study; no use of pan traps | |
| McCravy, 2018 | | Not relevant | | review article | |
| McCravy, Geroff, & Gibbs, 2016 | | Not relevant | | no use of pan traps | |
| McCravy, Geroff, & Gibbs, 2019 | | Relevant (duplicate) | | same data as in Geroff et al. (2014) | |
| McCravy & Ruholl, 2017 | | Relevant | |  | |
| McCune, Normandin, Mazerolle, & Fournier, 2020 | | Relevant | |  | |
| McKechnie, Thomsen, & Sargent, 2017 | | Relevant | |  | |
| McNeil et al., 2019 | | Not relevant | | no use of pan traps | |
| Meagher, Robert L., Jr. et al., 2020 | | Relevant | |  | |
| Meindl & Ashman, 2015 | | Relevant | |  | |
| Mendes et al., 2021 | | Not relevant | | not a bee study | |
| Mentone, Morini, Souza, & Braga, 2009 | | Not relevant | | not a bee study | |
| Methven, Langreney, & Prescott, 2012 | | Not relevant | | not a bee study; no use of pan traps | |
| Meyer, Unternährer, Arlettaz, Humbert, & Menz, 2017 | | Relevant | |  | |
| Middelkoop, van Marwijk, Kemp, & Bolhuis, 2019 | | Not relevant | | not a bee study; no use of pan traps | |
| Miguez & Amela García, 2019 | | Not relevant | | no use of pan traps | |
| Milam, Litvaitis, Warren, Keirstead, & King, 2018 | | Relevant | |  | |
| Milberg, Eriksson, & Bergman, 2021 | | Relevant | |  | |
| Miljanic et al., 2019 | | Relevant | |  | |
| Miller, Brosi, Magnacca, Daily, & Pejchar, 2015 | | Relevant | |  | |
| Minachilis et al., 2020 | | Relevant | |  | |
| Moisan-DeSerres, Chagnon, & Fournier, 2015 | | Relevant | |  | |
| Montgomery, Belitz, Guralnick, & Tingley, 2021 | | Not relevant | | review article | |
| Montoya, Parra, & Wolff, 2021 | | Not relevant | | not a bee study | |
| Morandin & Kremen, 2013 | | Relevant | |  | |
| Morandin & Winston, 2005 | | Relevant | |  | |
| Moreira et al., 2016 | | Relevant | |  | |
| Moroń et al., 2008 | | Relevant | |  | |
| Morrison, Izquierdo, Plaza, & González-Andújar, 2017 | | Relevant | |  | |
| Motyka, Wiśniowski, & Szczepko, 2016 | | Not relevant | | no use of pan traps | |
| Moylett, Youngsteadt, & Sorenson, 2020 | | Relevant | |  | |
| Mpondo, Ndakidemi, Pauly, & Treydte, 2021 | | Relevant | |  | |
| Munyuli, 2012 | | Relevant | |  | |
| Nachev, Stich, & Winter, 2013 | | Not relevant | | no use of pan traps | |
| Neame, Griswold, & Elle, 2013 | | Relevant | |  | |
| Nelson, Frost, & Nielsen, 2021 | | Relevant | |  | |
| Neves Jr. et al., 2021 | | Relevant | |  | |
| Ngo, Gibbs, Griswold, & Packer, 2013 | | Not relevant | | no use of pan traps | |
| Nielsen et al., 2011 | | Relevant | |  | |
| Noel et al., 2021 | | Relevant | |  | |
| Nol, Douglas, & Crins, 2006 | | Relevant | |  | |
| Nooten, Odanaka, & Rehan, 2020 | | Relevant | |  | |
| Normandin, Vereecken, Buddle, & Fournier, 2017 | | Relevant | |  | |
| Nuttman et al., 2011 | | Relevant | |  | |
| O'Connor et al., 2019 | | Relevant | |  | |
| Odanaka, Gibbs, Turley, Isaacs, & Brudvig, 2020 | | Relevant | |  | |
| Olson, Gibbs, & Schmidt, 2021 | | Relevant | |  | |
| Olynyk, Westwood, & Koper, 2021 | | Relevant | |  | |
| Osterman, Theodorou, Radzevičiūtė, Schnitker, & Paxton, 2021 | | Relevant | |  | |
| Padrón et al., 2021 | | Relevant | |  | |
| Pardee & Philpott, 2014 | | Relevant | |  | |
| Parys, Esquivel, Wright, Griswold, & Brewer, 2020 | | Relevant | |  | |
| Pascarella, 2017 | | Relevant | |  | |
| Pei et al., 2022 | | Relevant | |  | |
| Peng & Deng, 2014 | | Not relevant | | not a bee study; no use of pan traps | |
| Perillo, Neves, Antonini, & Martins, 2017 | | Relevant | |  | |
| Perrot, Gaba, Roncoroni, Gautier, & Bretagnolle, 2018 | | Relevant | |  | |
| Pfiffner, Ostermaier, Stoeckli, & Müller, 2018 | | Relevant | |  | |
| Phillips, Gaston, Bullock, & Osborne, 2019 | | Relevant | |  | |
| Pisanty, Scheuchl, & Dorchin, 2016 | | Not relevant | | no use of pan traps | |
| Plascencia & Philpott, 2017 | | Relevant | |  | |
| Popic, Davila, & Wardle, 2013 | | Relevant | |  | |
| Portman, Bruninga-Socolar, & Cariveau, 2020 | | Not relevant | | review article | |
| Prado, Ngo, Florez, & Collazo, 2017 | | Not relevant | | review article | |
| Prendergast & Hogendoorn, 2021 | | Not relevant | | review article | |
| Prendergast, Menz, Dixon, & Bateman, 2020 | | Relevant | |  | |
| Proesmans et al., 2019 | | Relevant | |  | |
| Quistberg, Bichier, & Philpott, 2016 | | Relevant | |  | |
| Ramello, Álvarez, Almada, & Lucia, 2021 | | Relevant | |  | |
| Ramírez-Freire, José Alanís-Flores, Ayala-Barajas, Quiroz-Martínez, & Gerardo Velazco-Macías, 2012 | | Not relevant | | study language is Spanish | |
| Rehan & Richards, 2010 | | Relevant | |  | |
| Rhoades et al., 2017 | | Relevant | |  | |
| M. H. Richards et al., 2011 | | Relevant | |  | |
| M. H. Richards, Vickruck, & Rehan, 2010 | | Relevant | |  | |
| Miriam H. Richards, Onuferko, & Rehan, 2015 | | Relevant | |  | |
| Ritchie, Ruppel, & Jha, 2016 | | Relevant | |  | |
| Roberts, King, & Milam, 2017 | | Relevant | |  | |
| Robertson et al., 2020 | | Not relevant | | not a bee study | |
| Rochlin, White, Reissen, Martheswaran, & Faraji, 2022 | | Relevant | |  | |
| Rodrigo Gómez et al., 2021 | | Relevant | |  | |
| Rodrigo Gómez, Ornosa, Selfa, Guara, & Polidori, 2016 | | Relevant | |  | |
| Rodríguez & Kouki, 2015 | | Relevant | |  | |
| Rogers, Tarpy, & Burrack, 2014 | | Relevant | |  | |
| Ropars et al., 2020 | | Relevant | |  | |
| Rösch, 2005 | | Not relevant | | not a bee study | |
| Rothwell & Ginsberg, 2019 | | Relevant | |  | |
| Roulston, Smith, & Brewster, 2007 | | Relevant | |  | |
| Rubene, Schroeder, & Ranius, 2015 | | Relevant | |  | |
| Rutgers-Kelly & Richards, 2013 | | Relevant (duplicate) | | same data as in M. H. Richards et al., 2011 | |
| Ruttan, Filazzola, & Lortie, 2016 | | Relevant | |  | |
| Sahli, Krushelnycky, Drake, & Taylor, 2016 | | Relevant | |  | |
| Samnegård, Hambäck, Eardley, Nemomissa, & Hylander, 2015 | | Relevant | |  | |
| Sanchez, Carrasco, La Spina, Pérez-Marcos, & Ortiz-Sánchez, 2019 | Relevant | |  | |  |
| Sardiñas & Kremen, 2014 | | Relevant | |  | |
| Saunders & Luck, 2013 | | Relevant | |  | |
| Saunders, Luck, & Mayfield, 2013 | | Relevant | |  | |
| Scherber, Beduschi, & Tscharntke, 2019 | | Relevant | |  | |
| Schlueter & Stewart, 2015 | | Relevant | |  | |
| Schubert, Bewersdorff, Luch, & Schulz, 2012 | | Not relevant | | not a bee study | |
| Schurr et al., 2021 | | Relevant | |  | |
| Shannak, Corsmeier, Kottmeier, & Al-azab, 2014 | | Not relevant | | not a bee study | |
| Shapiro, Tepedino, & Minckley, 2014 | | Relevant | |  | |
| Shi et al., 2021 | | Relevant | |  | |
| Shrestha et al., 2019 | | Relevant | |  | |
| Shrestha et al., 2021 | | Relevant | |  | |
| F. O. Silva, Kevan, Roque, Viana, & Kevan, 2010 | | Not relevant | | no use of pan traps | |
| W. R. Silva & Sazima, 1995 | | Not relevant | | not a bee study; no use of pan traps | |
| Simon et al., 2021 | | Relevant | |  | |
| Sing et al., 2016 | | Relevant | |  | |
| Sircom, Jothi, & Pinksen, 2018 | | Relevant | |  | |
| Sivakoff, Prajzner, & Gardiner, 2018 | | Relevant | |  | |
| Song, Moon, & Park, 2020 | | Not relevant | | not a bee study; no use of pan traps; language non-English | |
| Souza & Campos, 2008 | | Relevant | |  | |
| Spafford & Lortie, 2013 | | Relevant | |  | |
| St. Clair, Dolezal, O'Neal, & Toth, 2020 | | Relevant | |  | |
| Stein et al., 2018 | | Relevant | |  | |
| Stephenson, Dowling, & Krementz, 2020 | | Relevant | |  | |
| Stephenson, Griswold, Arduser, Dowling, & Krementz, 2018 | | Relevant | |  | |
| Straka, Rezkova, Batelka, & Kratochvíl, 2011 | | Relevant | |  | |
| Streinzer, Roth, Paulus, & Spaethe, 2019 | | Not relevant | | no use of pan traps | |
| Suárez et al., 2022 | | Not relevant | | no use of pan traps | |
| Szczepko, Kruk, & Wiśniowski, 2020 | | Not relevant | | not a bee study | |
| Taki, Kevan, & Ascher, 2007 | | Relevant | |  | |
| Talašová et al., 2018 | | Relevant | |  | |
| Tang et al., 2015 | | Relevant | |  | |
| Templ et al., 2019 | | Relevant | |  | |
| A. Thompson et al., 2021 | | Not relevant | | no use of pan traps | |
| H. Thompson, Schneider, Maus, Camata, & Wolf, 2020 | | Relevant | |  | |
| Todd, Gardiner, & Lindquist, 2016 | | Relevant | |  | |
| Toler, Evans, & Tepedino, 2005 | | Relevant | |  | |
| Torné-Noguera, Rodrigo, Osorio, & Bosch, 2016 | | Relevant | |  | |
| Török, Gallé, & Batáry, 2022 | | Relevant | |  | |
| Travis & Gonzalez, 2017 | | Not relevant | | meeting abstract | |
| Tuell, Ascher, & Isaacs, 2009 | | Relevant | |  | |
| Tuell & Isaacs, 2009 | | Relevant | |  | |
| Tuell & Isaacs, 2010 | | Relevant (duplicate) | | same data as in Tuell & Isaacs, 2009 | |
| Turo, Spring, Sivakoff, Delgado de la flor, Yvan A., & Gardiner, 2021 | | Relevant | |  | |
| Uemori, Mita, & Hishi, 2021 | | Relevant | |  | |
| Ugolini, Vignali, Castellini, & Lindström, 1996 | | Not relevant | | not a bee study; no use of pan traps | |
| Ulyshen, Horn, & Hanula, 2022 | | Relevant | |  | |
| Ulyshen, Pokswinski, & Hiers, 2020 | | Relevant | |  | |
| Ulyshen, Soon, & Hanula, 2010 | | Not relevant | | no use of pan traps | |
| Umair Sial et al., 2022 | | Relevant | |  | |
| Urban-Mead et al., 2021 | | Relevant | |  | |
| Joseph, Harris-Shultz, & Jespersen, 2020 | | Relevant | |  | |
| Venjakob, Ruedenauer, Klein, & Leonhardt, 2022 | | Not relevant | | not a bee study; no use of pan traps | |
| Vereecken et al., 2021 | | Relevant | |  | |
| Vickruck, Rehan, Sheffield, & Richards, 2011 | | Relevant | |  | |
| Vieira, Waichert, Williams, & Pitts, 2017 | | Not relevant | | not a bee study | |
| Vrdoljak & Samways, 2012 | | Relevant | |  | |
| Wagner, Ascher, & Bricker, 2014 | | Relevant | |  | |
| Wang, Hua, Wang, Wilcove, & Yu, 2019 | | Relevant | |  | |
| Watson, Wolf, & Ascher, 2011 | | Relevant | |  | |
| Wester, Stanway, & Pauw, 2009 | | Not relevant | | not a bee study; no use of pan traps | |
| Westerberg, Berglund, Jonason, & Milberg, 2021 | | Relevant | |  | |
| Westphal et al., 2008 | | Relevant | |  | |
| Wheelock & O'Neal, 2016 | | Relevant | |  | |
| Wheelock, Rey, & O'Neal, 2016 | | Relevant | |  | |
| Wilson, Griswold, & Messinger, 2008 | | Relevant | |  | |
| Wilson et al., 2016 | | Relevant | |  | |
| Wood, Holland, & Goulson, 2015 | | Relevant | |  | |
| Zhu, Yang, & Li, 2013 | | Not relevant | | no use of pan traps | |
| Zou, Bianchi, et al., 2017 | | Relevant | |  | |
| Zou, Xiao, et al., 2017 | | Relevant (duplicate) | | same data as in Zou, Bianchi, et al., 2017 | |
| Zumpf et al., 2021 | | Relevant | |  | |

References

Abbate, A., Campbell, J. W. [Joshua W.], Kimmel, C. B., & Kern, W. H. (2019). Urban development decreases bee abundance and diversity within coastal dune systems. *Global Ecology and Conservation*, *20*, e00711. https://doi.org/10.1016/j.gecco.2019.e00711

Abrahamczyk, S., Steudel, B., & Kessler, M. (2010). Sampling Hymenoptera along a precipitation gradient in tropical forests: The effectiveness of different coloured pan traps. *Entomologia Experimentalis Et Applicata*, *137*(3), 262–268. https://doi.org/10.1111/j.1570-7458.2010.01063.x

Acharya, R. S., Leslie, T., Fitting, E., Burke, J., Loftin, K., & Joshi, N. K. (2021). Color of Pan Trap Influences Sampling of Bees in Livestock Pasture Ecosystem. *BIOLOGY-BASEL*, *10*(5), 445. https://doi.org/10.3390/biology10050445

Ackermann, M., & Weigend, M. (2006). Nectar, floral morphology and pollination syndrome in Loasaceae subfam. Loasoideae (Cornales). *Annals of Botany*, *98*(3), 503–514. https://doi.org/10.1093/aob/mcl136

Adedoja, O., Kehinde, T., & Samways, M. J. (2019). Time since fire strongly and variously influences anthophilous insects in a fire-prone landscape. *Ecosphere*, *10*(9), e02849. https://doi.org/10.1002/ecs2.2849

Adedoja, O., Kehinde, T., & Samways, M. J. (2021). Age class of alien tree stands retained for mammal protection have differential effects on flower-visiting insect assemblages. *Insect Conservation and Diversity*, *14*(6), 814–824. https://doi.org/10.1111/icad.12517

Affek, A. N., Regulska, E., Kolaczkowska, E., Kowalska, A., & Affek, K. (2021). Pollination Potential of Riparian Hardwood Forests-A Multifaceted Field-Based Assessment in the Vistula Valley, Poland. *Forests*, *12*(7), 907. https://doi.org/10.3390/f12070907

Aguiar, A. J. C., & Ramos, K. S. (2020). Two New Species of Anthrenoides Ducke, 1907 (Hymenoptera: Apidae: Andreninae) with a Checklist of Andrenine Bees of Cerrado Savanna. *Annales Zoologici*, *70*(4), 561–572. https://doi.org/10.3161/00034541ANZ2020.70.4.006

Aizen, M. A., & Feinsinger, P. (1994). Habitat fragmentation, native insect pollinators, and feral honey bees in Argentine "Chaco Serrano". *Ecological Applications*, *4*(2), 378–392. https://doi.org/10.2307/1941941

Almeida, M. L. S., Carvalho, G. S., Novais, J. R., Storck-Tonon, D., Oliveira, M. L., Mahlmann, T., . . . Pereira, M. J. B. (2020). Contribution of the Cerrado as Habitat for Sunflower Pollinating Bees. *Sociobiology*, *67*(2), 281–291. https://doi.org/10.13102/sociobiology.v67i2.4865

Almeida, R. P. S., Arruda, F., V, Silva, D. P. [D. P.], & Coelho, B. W. T. [B. W. T.] (2019). Bees (Hymenoptera, Apoidea) in an Ecotonal Cerrado-Amazon Region in Brazil. *Sociobiology*, *66*(3), 457–466. https://doi.org/10.13102/sociobiology.v66i3.3463

Amy, C., Noël, G., Hatt, S., Uyttenbroeck, R., van de Meutter, F., Genoud, D., & Francis, F. (2018). Flower Strips in Wheat Intercropping System: Effect on Pollinator Abundance and Diversity in Belgium. *Insects*, *9*(3), 114. https://doi.org/10.3390/insects9030114

Andersson, P., Koffman, A., Sjödin, N. E., & Johansson, V. (2017). Roads may act as barriers to flying insects: Species composition of bees and wasps differs on two sides of a large highway. *Nature Conservation*. (18), 47–59. https://doi.org/10.3897/natureconservation.18.12314

Arnan, X., Bosch, J., Comas, L., Gracia, M., & Retana, J. (2011). Habitat determinants of abundance, structure and composition of flying Hymenoptera communities in mountain old-growth forests. *Insect Conservation and Diversity*, *4*(3), 200–211. https://doi.org/10.1111/j.1752-4598.2010.00123.x

Ashcroft, M. B., Gollan, J. R., & Batley, M. (2012). Combining citizen science, bioclimatic envelope models and observed habitat preferences to determine the distribution of an inconspicuous, recently detected introduced bee (Halictus smaragdulus Vachal Hymenoptera: Halictidae) in Australia. *Biological Invasions*, *14*(3), 515–527. https://doi.org/10.1007/s10530-011-0092-x

Assis, J. C. de, Toppa, R. H., Martines, M. R., Arena, M. V. N., Da Silva Souza, M., Carneiro, L. T., . . . Da Silva-Zacarin, E. C. M. (2021). The influence of climate and seasonality on bee communities: A complementary method for bee sampling in forest patches of an anthropic matrix. *International Journal of Tropical Insect Science*, *41*(1), 711–723. https://doi.org/10.1007/s42690-020-00261-1

Ávila-Gómez, E. S., Meléndez-Ramírez, V., Castellanos, I., Zuria, I., & Moreno, C. E. (2019). Prickly pear crops as bee diversity reservoirs and the role of bees in Opuntia fruit production. *Agriculture, Ecosystems & Environment*, *279*, 80–88. https://doi.org/10.1016/j.agee.2019.04.012

Bąk-Badowska, J. (2012). Spatial Diversification of Bee (Hymenoptera: Apoidea: Apiformes) Assemblages in Forest Communities of the Suchedniów-Oblęgorek Landscape Park. *Journal of Apicultural Science*, *56*(2), 89–106. https://doi.org/10.2478/v10289-012-0027-5

Ballare, K. M., Pope, N. S., Castilla, A. R., Cusser, S., Metz, R. P., & Jha, S. (2019). Utilizing field collected insects for next generation sequencing: Effects of sampling, storage, and DNA extraction methods. *Ecology and Evolution*, *9*(24), 13690–13705. https://doi.org/10.1002/ece3.5756

Banaszak, J., Motyka, E., & Szczepko, K. (2013). Andrena Florivaga Eversmann 1852 (Hymenoptera: Apoidea: Andrenidae) - A New Bee Species of the Genus Andrena in Poland. *Journal of Apicultural Science*, *57*(1), 45–50. https://doi.org/10.2478/jas-2013-0005

Bartholomew, C. S., & Prowell, D. (2005). Pan Compared to Malaise Trapping for Bees (Hymenoptera: Apoidea) in a Longleaf Pine Savanna. *Journal of the Kansas Entomological Society*, *78*(4), 390–392. https://doi.org/10.2317/0409.24.1

Bashir, M. A., Saeed, S., & Sajjad, A. (2013). Monitoring Hymenoptera and Diptera Pollinators in a sub-tropical forest of southern Punjab, Pakistan. *Pakistan Journal of Agricultural Sciences*, *50*(3), 359–366.

Bashir, M. A., Saeed, S., Sajjad, A., Khan, K. A., Ghramh, H. A., Shehzad, M. A., . . . Ansari, M. J. (2019). Insect pollinator diversity in four forested ecosystems of southern Punjab, Pakistan. *Saudi Journal of Biological Sciences*, *26*(7), 1835–1842. https://doi.org/10.1016/j.sjbs.2018.02.007

Basque, C., Cambou, S., Peron, F., Le Paih, L., Marzin, C., Hanaoka, K., . . . Lethuaut, L. (2019). Food preference and olfactory discrimination tests: A complementary approach to understand the drivers of hedonic responses in dogs. *Journal of Sensory Studies*, *34*(2). https://doi.org/10.1111/joss.12483

Basu, P., Parui, A. K., Chatterjee, S., Dutta, A., Chakraborty, P., Roberts, S., & Smith, B. (2016). Scale dependent drivers of wild bee diversity in tropical heterogeneous agricultural landscapes. *Ecology and Evolution*, *6*(19), 6983–6992. https://doi.org/10.1002/ece3.2360

Baum, K. A., & Wallen, K. E. (2011). Potential Bias in Pan Trapping as a Function of Floral Abundance. *Journal of the Kansas Entomological Society*, *84*(2), 155–159. https://doi.org/10.2317/JKES100629.1

Bergholz, K., Sittel, L.‑P., Ristow, M., Jeltsch, F., & Weiss, L. (2022). Pollinator guilds respond contrastingly at different scales to landscape parameters of land-use intensity. *Ecology and Evolution*, *12*(3), e8708. https://doi.org/10.1002/ece3.8708

Berglund, H.‑L., & Milberg, P. (2019). Sampling of flower-visiting insects: Poor correspondence between the catches of colour pan-trap and sweep netting. *European Journal of Entomology*, *116*, 425–431. https://doi.org/10.14411/eje.2019.043

Bessa, J., Hockings, K., & Biro, D. (2021). First Evidence of Chimpanzee Extractive Tool Use in Cantanhez, Guinea-Bissau: Cross-Community Variation in Honey Dipping. *Frontiers in Ecology and Evolution*, *9*, 625303. https://doi.org/10.3389/fevo.2021.625303

Bhandari, K. B., Longing, S. D. [Scott D.], & West, C. P. [Charles P.] (2020). Bees Occurring in Corn Production Fields Treated with Atoxigenic Aspergillus flavus (Texas, USA). *Agronomy*, *10*(4), 571. https://doi.org/10.3390/agronomy10040571

Bhandari, K. B., West, C. P. [C. P.], Longing, S. D. [S. D.], Brown, C. P., Green, P. E., & Barkowsky, E. (2018). Pollinator Abundance in Semiarid Pastures as Affected by Forage Species. *Crop Science*, *58*(6), 2665–2671. https://doi.org/10.2135/cropsci2018.06.0393

Biddinger, D. J., & Joshi, N. K. (2017). First report of native Astata unicolor (Hymenoptera: Crabronidae) predation on the nymphs and adults of the invasive brown marmorated stink bug (Hemiptera: Pentatomidae). *Florida Entomologist*, *100*(4), 809–812. https://doi.org/10.1653/024.100.0408

Boyer, K. J., Fragoso, F. P., Mabin, M. E. D., & Brunet, J. (2020). Netting and pan traps fail to identify the pollinator guild of an agricultural crop. *Scientific Reports*, *10*(1), 13819. https://doi.org/10.1038/s41598-020-70518-9

Braatz, E. Y., Gezon, Z. J., Rossetti, K., Maynard, L. T., Bremer, J. S., Hill, G. M., . . . Daniels, J. C. (2021). Bloom evenness modulates the influence of bloom abundance on insect community structure in suburban gardens. *PeerJ*, *9*, e11132. https://doi.org/10.7717/peerj.11132

Breland, S., Turley, N. E., Gibbs, J. [Jason], Isaacs, R., & Brudvig, L. A. (2018). Restoration increases bee abundance and richness but not pollination in remnant and post-agricultural woodlands. *Ecosphere*, *9*(9), e02435. https://doi.org/10.1002/ecs2.2435

Bried, J. T., & Dillon, A. M. (2012). Bee diversity in scrub oak patches 2 years after mow and herbicide treatment. *Insect Conservation and Diversity*, *5*(3), 237–243. https://doi.org/10.1111/j.1752-4598.2011.00154.x

Briggs, E. L., Baranski, C., Münzer Schaetz, O., Garrison, G., Collazo, J. A., & Youngsteadt, E. (2022). Estimating bee abundance: Can mark-recapture methods validate common sampling protocols? *Apidologie*, *53*(1), 10. https://doi.org/10.1007/s13592-022-00919-4

Brooks, D. R., & Nocera, J. J. (2020). Bumble bee (Bombus spp.) diversity differs between forested wetlands and clearcuts in the Acadian forest. *Canadian Journal of Forest Research*, *50*(12), 1399–1404. https://doi.org/10.1139/cjfr-2020-0094

Buchanan, A. L., Gibbs, J. [Jason], Komondy, L., & Szendrei, Z. (2017). Bee Community of Commercial Potato Fields in Michigan and Bombus impatiens Visitation to Neonicotinoid-Treated Potato Plants. *Insects*, *8*(1), 30. https://doi.org/10.3390/insects8010030

Buffington, M. L., Garretson, A., Kula, R. R., Gates, M. W., Carpenter, R., Smith, D. R., & Kula, A. A. R. (2021). Pan trap color preference across Hymenoptera in a forest clearing. *Entomologia Experimentalis Et Applicata*, *169*(3), 298–311. https://doi.org/10.1111/eea.13008

Bukovinszky, T., Verheijen, J., Zwerver, S., Klop, E., Biesmeijer, J. C., Wäckers, F. L., . . . Kleijn, D. (2017). Exploring the relationships between landscape complexity, wild bee species richness and reproduction, and pollination services along a complexity gradient in the Netherlands. *Biological Conservation*, *214*, 312–319. https://doi.org/10.1016/j.biocon.2017.08.027

Buri, P., Humbert, J.‑Y., & Arlettaz, R. (2014). Promoting pollinating insects in intensive agricultural matrices: Field-scale experimental manipulation of hay-meadow mowing regimes and its effects on bees. *PLOS ONE*, *9*(1), e85635. https://doi.org/10.1371/journal.pone.0085635

Calizaya-Melo, Y. A., Aguilar, M. L., & Lopez Tejeda, E. (2021). High Andean bees (Hymenoptera: Apoidea) in Arequipa, Peru. *ACTA BIOLOGICA COLOMBIANA*, *26*(3), 295–302. https://doi.org/10.15446/abc.v26n3.86011

Campbell, J. W. [J. W.], Hanula, J. L. [J. L.], & Waldrop, T. A. (2007). Effects of prescribed fire and fire surrogates on floral visiting insects of the blue ridge province in North Carolina. *Biological Conservation*, *134*(3), 393–404. https://doi.org/10.1016/j.biocon.2006.08.029

Campbell, J. W. [Joshua W.], Grodsky, S. M., Monroe, A. P., & Martin, J. A. (2021). Bee (Apoidea) community response to perennial grass treatments managed for livestock production and conservation. *Agriculture, Ecosystems & Environment*, *313*, 107391. https://doi.org/10.1016/j.agee.2021.107391

Campbell, J. W. [Joshua W.], Miller, D. A., & Martin, J. A. (2016). Switchgrass (Panicum virgatum) Intercropping within Managed Loblolly Pine (Pinus taeda) Does Not Affect Wild Bee Communities. *Insects*, *7*(4), 62. https://doi.org/10.3390/insects7040062

Campbell, J. W. [Joshua W.], Milne, M., Dinh, B. T., Daniels, J. C., & Ellis, J. D. (2020). Spider (Araneae) abundance and species richness comparison between native wildflower plantings and fallow controls in intensively managed agricultural areas. *Arthropod-Plant Interactions*, *14*(2), 263–274. https://doi.org/10.1007/s11829-019-09725-9

Campbell, J. W. [Joshua W.], Vigueira, P. A., Viguiera, C. C., & Greenberg, C. H. (2018). The Effects of Repeated Prescribed Fire and Thinning on Bees, Wasps, and Other Flower Visitors in the Understory and Midstory of a Temperate Forest in North Carolina. *Forest Science*, *64*(3), 299–306. https://doi.org/10.1093/forsci/fxx008

Cane, J. H., Kervin, L. J., & Minckley, R. (2013). Sensitivity of Systematic Net Sampling for Detecting Shifting Patterns of Incidence and Abundance in a Floral Guild of Bees at Larrea tridentata. *Journal of the Kansas Entomological Society*, *86*(2), 171–180. https://doi.org/10.2317/JKES121126.1

Cane, J. H., Minckley, R. L., & Kervin, L. J. (2000). Sampling bees (Hymenoptera : Apiformes) for pollinator community studies: Pitfalls of pan-trapping. *Journal of the Kansas Entomological Society*, *73*(4), 225–231.

Carper, A. L., Adler, L. S., Warren, P. S., & Irwin, R. E. (2014). Effects of Suburbanization on Forest Bee Communities. *Environmental Entomology*, *43*(2), 253–262. https://doi.org/10.1603/EN13078

Castro, J., Tortosa, F. S., Jimenez, J., & Carpio, A. J. (2017). Spring evaluation of three sampling methods to estimate family richness and abundance of arthropods in olive groves. *Animal Biodiversity and Conservation*, *40*(2), 193–210. https://doi.org/10.32800/abc.2017.40.0193

Cavalcante Neto, J. L., Steenbergen, B., Roberto Zamuner, A., & Tudella, E. (2021). Wii training versus non-Wii task-specific training on motor learning in children with developmental coordination disorder: A randomized controlled trial. *Annals of Physical and Rehabilitation Medicine*, *64*(2), 101390. https://doi.org/10.1016/j.rehab.2020.03.013

Ceballos, M. R., Gorricho, J. L., Palma Gamboa, O., Huerta, M. K., Rivas, D., & Erazo Rodas, M. (2015). Fuzzy System of Irrigation Applied to the Growth of Habanero Pepper (Capsicum chinense Jacq.) under Protected Conditions in Yucatan, Mexico. *International Journal of Distributed Sensor Networks*, *11*(6), 123543. https://doi.org/10.1155/2015/123543

Choate, B. A., Hickman, P. L., & Moretti, E. A. (2018). Wild bee species abundance and richness across an urban-rural gradient. *Journal of Insect Conservation*, *22*(3-4), 391–403. https://doi.org/10.1007/s10841-018-0068-6

Classen, A., Peters, M. K., Kindeketa, W. J., Appelhans, T., Eardley, C. D., Gikungu, M. W., . . . Steffan-Dewenter, I. (2015). Temperature versus resource constraints: Which factors determine bee diversity on Mount Kilimanjaro, Tanzania? *Global Ecology and Biogeography*, *24*(6), 642–652. https://doi.org/10.1111/geb.12286

Cohen, H., Egerer, M., Thomas, S.‑S., & Philpott, S. M. [Stacy M.] (2022). Local and landscape features constrain the trait and taxonomic diversity of urban bees. *Landscape Ecology*, *37*(2), 583–599. https://doi.org/10.1007/s10980-021-01370-z

Connelly, H., Poveda, K., & Loeb, G. (2015). Landscape simplification decreases wild bee pollination services to strawberry. *Agriculture, Ecosystems & Environment*, *211*, 51–56. https://doi.org/10.1016/j.agee.2015.05.004

Cook, S. P., Birch, S. M., Merickel, F. W., Lowe, C. C., & Page-Dumroese, D. (2011). Bumble bee (Hymenoptera: Apidae) community structure on two sagebrush steppe sites in southern Idaho. *Pan-Pacific Entomologist*, *87*(3), 161–171. https://doi.org/10.3956/2010-31.1

Cope, G. C., Campbell, J. W. [Joshua W.], Grodsky, S. M., & Ellis, J. D. (2019). Evaluation of nest-site selection of ground-nesting bees and wasps (Hymenoptera) using emergence traps. *The Canadian Entomologist*, *151*(2), 260–271. https://doi.org/10.4039/tce.2019.3

Corbet, S. A., Chen, F.‑F., Chang, F.‑F., & Huang, S.‑Q. (2020). Transient dehydration of pollen carried by hot bees impedes fertilization. *Arthropod-Plant Interactions*, *14*(2), 207–214. https://doi.org/10.1007/s11829-019-09726-8

Corbin, L. A.‑J., Awde, D. N., & Richards, M. H. [Miriam H.] (2021). Phenological and social characterization of three Lasioglossum (Dialictus) species inferred from long-term trapping collections. *Journal of Hymenoptera Research*, *88*, 17–38. https://doi.org/10.3897/jhr.88.73220

Costa, E. M., Araujo, E. L., Fernandes, D. R. R., Silva, P. A. F., & Sales Junior, R. (2016). Diversity and sampling methods for Hymenoptera in a watermelon crop in the semiarid region. *Horticultura Brasileira*, *34*(2), 257–264. https://doi.org/10.1590/S0102-053620160000200017

Creedy, T. J., Norman, H., Tang, C. Q., Qing Chin, K., Andujar, C., Arribas, P., . . . Vogler, A. P. (2020). A validated workflow for rapid taxonomic assignment and monitoring of a national fauna of bees (Apiformes) using high throughput DNA barcoding. *Molecular Ecology Resources*, *20*(1), 40–53. https://doi.org/10.1111/1755-0998.13056

Cruz-Sanchez, M. A. [M. A.], Asis, J. D. [J. D.], Gonzalez, J. A. [J. A.], Tormos, J. [J.], & Gayubo, S. F. [S. F.] (2014). Wildfires: Its influence on the diversity parameters of predatory-insect communities in a Mediterranean agroecosystem of European interest. *Journal of Insect Conservation*, *18*(5), 903–908. https://doi.org/10.1007/s10841-014-9698-5

Cruz-Sanchez, M. A. [Miguel A.], Asis, J. D. [Josep D.], Gayubo, S. F. [Severiano F.], Tormos, J. [Jose], & Gonzalez, J. A. [Jose A.] (2011). The effects of wildfire on Spheciformes wasp community structure: The importance of local habitat conditions. *Journal of Insect Conservation*, *15*(4), 487–503. https://doi.org/10.1007/s10841-010-9322-2

Cunningham-Minnick, M. J., Peters, V. E., & Crist, T. O. (2020). Bee communities and pollination services in adjacent crop fields following flower removal in an invasive forest shrub. *Ecological Applications*, *30*(4), e02078. https://doi.org/10.1002/eap.2078

Curtis, K., Bowie, M. H., & Hodge, S. (2019). Can native plantings encourage native and beneficial invertebrates on Canterbury dairy farms? *New Zealand Entomologist*, *42*(2), 67–78. https://doi.org/10.1080/00779962.2019.1660450

Dafni, A., & Kevan, P. G. [Peter G.] (1997). Flower size and shape: Implications in pollination. *Israel Journal of Plant Sciences*, *45*(2-3), 201–212. https://doi.org/10.1080/07929978.1997.10676684

Dafni, A., & Potts, S. G. [Simon G.] (2004). The Role of Flower Inclination, Depth, and Height in the Preferences of a Pollinating Beetle (Coleoptera: Glaphyridae). *Journal of Insect Behavior*, *17*(6), 823–834. https://doi.org/10.1023/B:JOIR.0000048991.45453.73

Davis, E. S., Kelly, R., Maggs, C. A., & Stout, J. C. (2018). Contrasting impacts of highly invasive plant species on flower-visiting insect communities. *Biodiversity and Conservation*, *27*(8), 2069–2085. https://doi.org/10.1007/s10531-018-1525-y

Decker, B. L., & Harmon-Threatt, A. N. (2019). Growing or dormant season burns: The effects of burn season on bee and plant communities. *Biodiversity and Conservation*, *28*(13), 3621–3631. https://doi.org/10.1007/s10531-019-01840-6

Delphia, C. M., Griswold, T., Reese, E. G., O'Neill, K. M., & Burkle, L. A. (2019). Checklist of bees (Hymenoptera: Apoidea) from small diversified vegetable farms in south-western Montana. *Biodiversity Data Journal*, *7*, e30062. https://doi.org/10.3897/BDJ.7.e30062

Demirci, Ş., Caner-Saltık, E. N., Türkmenoğlu, A., Özçilingir-Akgün, S., & Bakırer, Ö. (2004). Raw Material Characteristics and Technological Properties of some Medieval Glazed Ceramics and Tiles in Anatolia. *Key Engineering Materials*, *264-268*, 2395–2398. https://doi.org/10.4028/www.scientific.net/KEM.264-268.2395

Dingha, B. N., Jackai, L. E., Amoah, B. A., & Akotsen-Mensah, C. (2021). Pollinators on Cowpea Vigna unguiculata: Implications for Intercropping to Enhance Biodiversity. *Insects*, *12*(1), 54. https://doi.org/10.3390/insects12010054

Dingha, B. N., Omaliko, P. C., Amoah, B. A., Jackai, L. E., & Shrestha, D. (2021). Evaluation of Cowpea (Vigna unguiculata) in an Intercropping System as Pollinator Enhancer for Increased Crop Yield. *Sustainability*, *13*(17), 9612. https://doi.org/10.3390/su13179612

Dirrigl, Frank J., Jr. (2012). Effectiveness of Pan Trapping as a Rapid Bioinventory Method of Freshwater Shoreline Insects of Subtropical Texas. *Southwestern Entomologist*, *37*(2), 133–139. https://doi.org/10.3958/059.037.0205

Dogan, A., & Havvatoglu, Y. (2003). Investigation of Crystallographical Parameters Associated with FCC to BCC Martensitic Transformation in Fe-24.5 at % Pt Alloy. *Canadian Metallurgical Quarterly*, *42*(2), 231–234. https://doi.org/10.1179/cmq.2003.42.2.231

Donovall, Leo R., III, & vanEngelsdorp, D. (2010). A Checklist of the Bees (Hymenoptera: Apoidea) of Pennsylvania. *Journal of the Kansas Entomological Society*, *83*(1), 7–24. https://doi.org/10.2317/JKES808.29.1

Droege, S., Tepedino, V. J. [Vincent J.], LeBuhn, G., Link, W., Minckley, R. L., Chen, Q., & Conrad, C. (2010). Spatial patterns of bee captures in North American bowl trapping surveys. *Insect Conservation and Diversity*, *3*(1), 15–23. https://doi.org/10.1111/j.1752-4598.2009.00074.x

Dubet da Silva Mouga, Denise Monique, & Warkentin, M. (2016). Bee community and associated flora in lowland rain forest and mangrove in southern Brazil. *Revue D'ecologie (Terre Et Vie)*, *71*(4), 385–396.

Easton, A. H., & Goulson, D. [Dave] (2013). The Neonicotinoid Insecticide Imidacloprid Repels Pollinating Flies and Beetles at Field-Realistic Concentrations. *PLOS ONE*, *8*(1), e54819. https://doi.org/10.1371/journal.pone.0054819

Echezona, B. C., & Offordile, J. I. (2011). Responses of flea beetles (Podagrica spp.) and okra plants (Abelmoschus esculentus L. Moench) to differently coloured polyethylene shades. *International Journal of Pest Management*, *57*(2), 161–168. https://doi.org/10.1080/09670874.2010.547282

Eckerter, T., Buse, J., Bauhus, J., Förschler, M. I., & Klein, A. M. (2021). Wild bees benefit from structural complexity enhancement in a forest restoration experiment. *Forest Ecology and Management*, *496*, 119412. https://doi.org/10.1016/j.foreco.2021.119412

Elwell, S. L., Griswold, T., & Elle, E. (2016). Habitat type plays a greater role than livestock grazing in structuring shrubsteppe plant–pollinator communities. *Journal of Insect Conservation*, *20*(3), 515–525. https://doi.org/10.1007/s10841-016-9884-8

Estienne, V., Mundry, R., Kühl, H. S., & Boesch, C. (2017). Exploitation of underground bee nests by three sympatric consumers in Loango National Park, Gabon. *Biotropica*, *49*(1), 101–109. https://doi.org/10.1111/btp.12354

Estienne, V., Robira, B., Mundry, R., Deschner, T., & Boesch, C. (2019). Acquisition of a complex extractive technique by the immature chimpanzees of Loango National Park, Gabon. *Animal Behaviour*, *147*, 61–76. https://doi.org/10.1016/j.anbehav.2018.11.002

Estienne, V., Stephens, C., & Boesch, C. (2017). Extraction of honey from underground bee nests by central African chimpanzees (Pan troglodytes troglodytes) in Loango National Park, Gabon: Techniques and individual differences. *American Journal of Primatology*, *79*(8), e22672. https://doi.org/10.1002/ajp.22672

Fan, X., Hao, Q., Jin, R., Huang, H., Luo, Z., Yang, X., . . . Qiu, T. (2017). Assembly of gold nanoparticles into aluminum nanobowl array. *Scientific Reports*, *7*(1), 2322. https://doi.org/10.1038/s41598-017-02552-z

Fisogni, A., Hautekèete, N., Piquot, Y., Brun, M., Vanappelghem, C., Michez, D., & Massol, F. (2020). Urbanization drives an early spring for plants but not for pollinators. *Oikos*, *129*(11), 1681–1691. https://doi.org/10.1111/oik.07274

Forss, S., Motes-Rodrigo, A., Hrubesch, C., & Tennie, C. (2020). Chimpanzees' (Pan troglodytes) problem-solving skills are influenced by housing facility and captive care duration. *PeerJ*, *8*, e10263. https://doi.org/10.7717/peerj.10263

Fortel, L., Henry, M., Guilbaud, L., Guirao, A. L., Kuhlmann, M., Mouret, H., . . . Vaissière, B. E. (2014). Decreasing abundance, increasing diversity and changing structure of the wild bee community (Hymenoptera: Anthophila) along an urbanization gradient. *PLOS ONE*, *9*(8), e104679. https://doi.org/10.1371/journal.pone.0104679

Fox, K., Anderson, K. M., Andres, R., Foster, M. C., Foster, C. E., Vik, D., . . . Harris, M. O. (2015). Nectar Robbery and Thievery in the Hawk Moth (Lepidoptera: Sphingidae)-Pollinated Western Prairie Fringed Orchid Platanthera praeclara. *Annals of the Entomological Society of America*, *108*(6), 1000–1013. https://doi.org/10.1093/aesa/sav093

Frampton, M., Droege, S., Conrad, T., Prager, S., & Richards, M. H. [Miriam H.] (2008). Evaluation of Specimen Preservatives for DNA Analyses of Bees. *Journal of Hymenoptera Research*, *17*(2), 195–200.

Franceschinelli, E. V., Bergamini, L. L., Silva-Neto, C. M., Elias, M. A. S., Moreira, G. L., Morais, J. M., & Mesquita-Neto, J. N. (2019). Native bee fauna of tomato crops: A comparison of active sampling and pan trapping methods. *Iheringia Série Zoologia*, *109.* https://doi.org/10.1590/1678-4766e2019030

Galbraith, S. M., Griswold, T., Price, W. J., & Bosque-Pérez, N. A. [Nilsa A.] (2020). Biodiversity and community composition of native bee populations vary among human-dominated land uses within the seasonally dry tropics. *Journal of Insect Conservation*, *24*(6), 1045–1059. https://doi.org/10.1007/s10841-020-00274-8

Garibaldi, L. A., Pérez-Méndez, N., Cordeiro, G. D., Hughes, A., Orr, M., Alves-Dos-Santos, I., . . . Viana, B. F. [Blandina Felipe] (2021). Negative impacts of dominance on bee communities: Does the influence of invasive honey bees differ from native bees? *Ecology*, *102*(12), e03526. https://doi.org/10.1002/ecy.3526

Garratt, M. P. D. [M. P. D.], Potts, S. G. [S. G.], Banks, G., Hawes, C., Breeze, T. D. [T. D.], O'Connor, R. S. [R. S.], & Carvell, C. [C.] (2019). Capacity and willingness of farmers and citizen scientists to monitor crop pollinators and pollination services. *Global Ecology and Conservation*, *20*, e00781. https://doi.org/10.1016/j.gecco.2019.e00781

Geeraert, L., Aerts, R., Berecha, G., Daba, G., Fruyt, N. de, D'hollander, J., . . . Honnay, O. (2020). Effects of landscape composition on bee communities and coffee pollination in Coffea arabica production forests in southwestern Ethiopia. *Agriculture, Ecosystems & Environment*, *288*, 106706. https://doi.org/10.1016/j.agee.2019.106706

Gerner, E. E., & Sargent, R. D. (2022). Local plant richness predicts bee abundance and diversity in a study of urban residential yards. *Basic and Applied Ecology*, *58*, 64–73. https://doi.org/10.1016/j.baae.2021.11.004

Geroff, R. K., Gibbs, J. [Jason], & McCravy, K. W. (2014). Assessing bee (Hymenoptera: Apoidea) diversity of an Illinois restored tallgrass prairie: Methodology and conservation considerations. *Journal of Insect Conservation*, *18*(5), 951–964. https://doi.org/10.1007/s10841-014-9703-z

Gervais, A., Chagnon, M., & Fournier, V. [Valerie] (2018). Diversity and Pollen Loads of Flower Flies (Diptera: Syrphidae) in Cranberry Crops. *Annals of the Entomological Society of America*, *111*(6), 326–334. https://doi.org/10.1093/aesa/say027

Gervais, A., Fournier, V. [Valérie], Sheffield, C. S. [Cory S.], & Chagnon, M. (2017). Assessing Wild Bee Biodiversity in Cranberry Agroenvironments: Influence of Natural Habitats. *Journal of Economic Entomology*, *110*(4), 1424–1432. https://doi.org/10.1093/jee/tox173

Geslin, B., Le Féon, V., Folschweiller, M., Flacher, F., Carmignac, D., Motard, E., . . . Dajoz, I. (2016). The proportion of impervious surfaces at the landscape scale structures wild bee assemblages in a densely populated region. *Ecology and Evolution*, *6*(18), 6599–6615. https://doi.org/10.1002/ece3.2374

Geslin, B., Le Féon, V., Kuhlmann, M., Vaissière, B. E., & Dajoz, I. (2015). The bee fauna of large parks in downtown Paris, France. *Annales De La Société Entomologique De France*, *51*(5-6), 487–493. https://doi.org/10.1080/00379271.2016.1146632

Geslin, B., Oddie, M., Folschweiller, M., Legras, G., Seymour, C. L., van Veen, F., & Thébault, E. (2016). Spatiotemporal changes in flying insect abundance and their functional diversity as a function of distance to natural habitats in a mass flowering crop. *Agriculture, Ecosystems & Environment*, *229*, 21–29. https://doi.org/10.1016/j.agee.2016.05.010

Gezon, Z. J., Wyman, E. S., Ascher, J. S. [John S.], Inouye, D. W., & Irwin, R. E. (2015). The effect of repeated, lethal sampling on wild bee abundance and diversity. *Methods in Ecology and Evolution*, *6*(9), 1044–1054. https://doi.org/10.1111/2041-210X.12375

Giles, V., & Ascher, J. S. [John S.] (2006). A Survey of the Bees of the Black Rock Forest Preserve, New York (Hymenoptera: Apoidea). *Journal of Hymenoptera Research*, *15*(2), 208–231.

Gill, K. A., & O'Neal, M. E. [M. E.] (2015). Survey of Soybean Insect Pollinators: Community Identification and Sampling Method Analysis. *Environmental Entomology*, *44*(3), 488–498. https://doi.org/10.1093/ee/nvv001

Goldstein, P. Z., & Ascher, J. S. [John S.] (2016). Taxonomic and Behavioral Composition of an Island Fauna: A Survey of Bees (Hymenoptera: Apoidea: Anthophila) on Martha's Vineyard, Massachusetts. *Proceedings of the Entomological Society of Washington*, *118*(1), 37–92. https://doi.org/10.4289/0013-8797.118.1.37

Goldstein, P. Z., & Scott, V. L. (2015). Taxonomic and Behavioral Components of Faunal Comparisons Over Time: The Bees (Hymenoptera: Anthophila) of Boulder County, Colorado, Past and Present. *Proceedings of the Entomological Society of Washington*, *117*(3), 290–346. https://doi.org/10.4289/0013-8797.117.3.290

Gollan, J. R., Ashcroft, M. B., & Batley, M. (2011). Comparison of yellow and white pan traps in surveys of bee fauna in New South Wales, Australia (Hymenoptera: Apoidea: Anthophila). *Australian Journal of Entomology*, *50*, 174–178. https://doi.org/10.1111/j.1440-6055.2010.00797.x

Gonçalves, R. B., Sydney, N. V., Oliveira, P. S., & Artmann, N. O. (2014). Bee and wasp responses to a fragmented landscape in southern Brazil. *Journal of Insect Conservation*, *18*(6), 1193–1201. https://doi.org/10.1007/s10841-014-9730-9

Gonzalez, V. H. [Victor H.], Osborn, A. L., Brown, E. R., Pavlick, C. R., Enríquez, E., Tscheulin, T., . . . Barthell, J. F. (2020). Effect of pan trap size on the diversity of sampled bees and abundance of bycatch. *Journal of Insect Conservation*, *24*(3), 409–420. https://doi.org/10.1007/s10841-020-00224-4

Gonzalez, V. H. [Victor H.], Park, K. E., Çakmak, I., Hranitz, J. M., & Barthell, J. F. (2016). Pan traps and bee body size in unmanaged urban habitats. *Journal of Hymenoptera Research*, *51*, 241–247. https://doi.org/10.3897/jhr.51.9353

González, E., Salvo, A., & Valladares, G. (2015). Sharing enemies: Evidence of forest contribution to natural enemy communities in crops, at different spatial scales. *Insect Conservation and Diversity*, *8*(4), 359–366. https://doi.org/10.1111/icad.12117

Graham, K. K., Gibbs, J. [Jason], Wilson, J., May, E., & Isaacs, R. (2021). Resampling of wild bees across fifteen years reveals variable species declines and recoveries after extreme weather. *Agriculture, Ecosystems & Environment*, *317*, 107470. https://doi.org/10.1016/j.agee.2021.107470

Griffiths-Lee, J., Nicholls, E., & Goulson, D. [Dave] (2022). Sown mini-meadows increase pollinator diversity in gardens. *Journal of Insect Conservation*, *26*(2), 299–314. https://doi.org/10.1007/s10841-022-00387-2

Gruber, T. (2016). Great Apes Do Not Learn Novel Tool Use Easily: Conservatism, Functional Fixedness, or Cultural Influence? *International Journal of Primatology*, *37*(2), 296–316. https://doi.org/10.1007/s10764-016-9902-4

Grundel, R., Frohnapple, K. J., Jean, R. P., & Pavlovic, N. B. (2011). Effectiveness of Bowl Trapping and Netting for Inventory of a Bee Community. *Environmental Entomology*, *40*(2), 374–380. https://doi.org/10.1603/EN09278

Grundel, R., Jean, R. P., Frohnapple, K. J., Gibbs, J. [Jason], Glowacki, G. A., & Pavlovic, N. B. (2011). A Survey of Bees (Hymenoptera: Apoidea) of the Indiana Dunes and Northwest Indiana, USA. *Journal of the Kansas Entomological Society*, *84*(2), 105–138. https://doi.org/10.2317/JKES101027.1

Habel, J. C., & Ulrich, W. (2020). Ecosystem functions in natural and anthropogenic ecosystems across the East African coastal forest landscape. *Biotropica*, *52*(4), 598–607. https://doi.org/10.1111/btp.12780

Habel, J. C., & Ulrich, W. (2021). Ecosystem functions in degraded riparian forests of southeastern Kenya. *Ecology and Evolution*, *11*(18), 12665–12675. https://doi.org/10.1002/ece3.8011

Haberski, A., Hagelin, J. C., Barger, C. P., Sikes, D. S., & DuBour, K. A. (2021). An efficient method for sampling aerial arthropods at nest sites of an insectivorous songbird in steep decline. *Avian Conservation and Ecology*, *16*(2). https://doi.org/10.5751/ACE-01849-160201

Halinski, R. [R.], dos Santos, C. F., Kaehler, T. G., & Blochtein, B. [B.] (2018). Influence of Wild Bee Diversity on Canola Crop Yields. *Sociobiology*, *65*(4), 751–759. https://doi.org/10.13102/sociobiology.v65i4.3467

Halinski, R. [Rosana], Dorneles, A. L., & Blochtein, B. [Betina] (2015). Bee assemblage in habitats associated with Brassica napus L. *Revista Brasileira De Entomologia*, *59*(3), 222–228. https://doi.org/10.1016/j.rbe.2015.07.001

Hall, H. G. (2016). Color Preferences of Bees Captured in Pan Traps. *Journal of the Kansas Entomological Society*, *89*(3), 273–276. https://doi.org/10.2317/JKESD1600022.1

Hall, H. G., & Ascher, J. S. [John S.] (2010). Surveys of bees (Hymenoptera: Apoidea: Anthophila) in natural areas of Alachua County in north-central Florida. *Florida Entomologist*, *93*(4), 609–629. https://doi.org/10.1653/024.093.0419

Hall, H. G., & Ascher, J. S. [John S.] (2011). Surveys of wild bees (Hymenoptera: Apoidea: Anthophila) in organic farms of Alachua County in north-central Florida. *Florida Entomologist*, *94*(3), 539–552. https://doi.org/10.1653/024.094.0319

Hall, H. G., & Ascher, J. S. [John S.] (2014). The Distinctive Bee Fauna (Hymenoptera: Apoidea: Anthophila) of Sandhill Habitat at the Ordway-Swisher Biological Station in North-Central Florida. *Journal of the Kansas Entomological Society*, *87*(1), 1–21. https://doi.org/10.2317/JKES130224.1

Hall, M. (2018). Blue and yellow vane traps differ in their sampling effectiveness for wild bees in both open and wooded habitats. *Agricultural and Forest Entomology*, *20*(4), 487–495. https://doi.org/10.1111/afe.12281

Hall, M. A., & Reboud, E. L. (2019). High sampling effectiveness for non-bee flower visitors using vane traps in both open and wooded habitats. *Austral Entomology*, *58*(4), 836–847. https://doi.org/10.1111/aen.12416

Hanula, J. L. [James L.], & Horn, S. (2011). Removing an invasive shrub (Chinese privet) increases native bee diversity and abundance in riparian forests of the southeastern United States. *Insect Conservation and Diversity*, *4*(4), 275–283. https://doi.org/10.1111/j.1752-4598.2011.00131.x

Hanula, J. L. [James L.], Horn, S., & O'Brien, J. J. (2015). Have changing forests conditions contributed to pollinator decline in the southeastern United States? *Forest Ecology and Management*, *348*, 142–152. https://doi.org/10.1016/j.foreco.2015.03.044

Happe, A.‑K., Riesch, F., Rösch, V., Gallé, R., Tscharntke, T., & Batáry, P. (2018). Small-scale agricultural landscapes and organic management support wild bee communities of cereal field boundaries. *Agriculture, Ecosystems & Environment*, *254*, 92–98. https://doi.org/10.1016/j.agee.2017.11.019

Harris, B. A., Braman, S. K., & Pennisi, S. V. (2017). Pan Trap Designs for Monitoring Pollinators and Other Beneficial Insects in Conservation Gardens. *Journal of Entomological Science*, *52*(1), 9–14. https://doi.org/10.18474/JES16-13.1

Hatten, T. D., Looney, C., Strange, J. P., & Bosque-Pérez, N. A. [N. A.] (2013). Bumble bee fauna of Palouse Prairie: Survey of native bee pollinators in a fragmented ecosystem. *Journal of Insect Science (Online)*, *13*, 26. https://doi.org/10.1673/031.013.2601

Hendrychová, M., & Bogusch, P. (2016). Combination of reclaimed and unreclaimed sites is the best practice for protection of aculeate Hymenoptera species on brown coal spoil heaps. *Journal of Insect Conservation*, *20*(5), 807–820. https://doi.org/10.1007/s10841-016-9912-8

Heneberg, P., & Bogusch, P. (2014). To enrich or not to enrich? Are there any benefits of using multiple colors of pan traps when sampling aculeate Hymenoptera? *Journal of Insect Conservation*, *18*(6), 1123–1136. https://doi.org/10.1007/s10841-014-9723-8

Heneberg, P., & Bogusch, P. (2020). Identification of a previously overlooked anthropogenic habitat that attracts diverse assemblages of threatened bees and wasps. *Ecological Engineering*, *147*, 105759. https://doi.org/10.1016/j.ecoleng.2020.105759

Heneberg, P., Bogusch, P., & Řezáč, M. (2016). Off-road motorcycle circuits support long-term persistence of bees and wasps (Hymenoptera: Aculeata) of open landscape at newly formed refugia within otherwise afforested temperate landscape. *Ecological Engineering*, *93*, 187–198. https://doi.org/10.1016/j.ecoleng.2016.05.026

Heneberg, P., Bogusch, P., & Řezáč, M. (2017). Roadside verges can support spontaneous establishment of steppe-like habitats hosting diverse assemblages of bees and wasps (Hymenoptera: Aculeata) in an intensively cultivated central European landscape. *Biodiversity and Conservation*, *26*(4), 843–864. https://doi.org/10.1007/s10531-016-1275-7

Heneberg, P., Bogusch, P., & Řezáč, M. (2019). Tiny fragments of acidophilous steppic grasslands serve as yet unknown habitats of endangered aeolian sand specialists among Aculeata (Hymenoptera). *Biodiversity and Conservation*, *28*(1), 183–195. https://doi.org/10.1007/s10531-018-1646-3

Heneberg, P., Bogusch, P., Tauchmanová, P., Řezáč, M., & Astapenková, A. (2017). Common reed (Phragmites australis) gall as the limiting nesting resource of rare wetland bees and wasps (Hymenoptera: Aculeata & Evanioidea) in Central Europe. *Ecological Engineering*, *108*, 100–113. https://doi.org/10.1016/j.ecoleng.2017.08.014

Hevia, V., Bosch, J., Azcárate, F. M., Fernández, E., Rodrigo, A., Barril-Graells, H., & González, J. A. (2016). Bee diversity and abundance in a livestock drove road and its impact on pollination and seed set in adjacent sunflower fields. *Agriculture, Ecosystems & Environment*, *232*, 336–344. https://doi.org/10.1016/j.agee.2016.08.021

Hevia, V., Carmona, C. P., Azcárate, F. M., Heredia, R., & González, J. A. (2021). Role of floral strips and semi-natural habitats as enhancers of wild bee functional diversity in intensive agricultural landscapes. *Agriculture, Ecosystems & Environment*, *319*, 107544. https://doi.org/10.1016/j.agee.2021.107544

Hong, W., & Li, D.‑Z. (2005). Pollination biology of four Pedicularis species (Scrophulariaceae) in northwestern Yunnan, China. *Annals of the Missouri Botanical Garden*, *92*(1), 127–138.

Hopwood, J. L. (2008). The contribution of roadside grassland restorations to native bee conservation. *Biological Conservation*, *141*(10), 2632–2640. https://doi.org/10.1016/j.biocon.2008.07.026

Howard, S. R., Nisal Ratnayake, M., Dyer, A. G., Garcia, J. E., & Dorin, A. (2021). Towards precision apiculture: Traditional and technological insect monitoring methods in strawberry and raspberry crop polytunnels tell different pollination stories. *PLOS ONE*, *16*(5), e0251572. https://doi.org/10.1371/journal.pone.0251572

Hudson, J., Horn, S., & Hanula, J. L. [James L.] (2020). Assessing the Efficiency of Pan Traps for Collecting Bees (Hymenoptera: Apoidea). *Journal of Entomological Science*, *55*(3), 321–328. https://doi.org/10.18474/0749-8004-55.3.321

Hudson, J. R., Hanula, J. L. [James L.], & Horn, S. (2013). Removing Chinese privet from riparian forests still benefits pollinators five years later. *Biological Conservation*, *167*, 355–362. https://doi.org/10.1016/j.biocon.2013.09.001

Hung, K.‑L. J., Sandoval, S. S., Ascher, J. S. [John S.], & Holway, D. A. (2021). Joint Impacts of Drought and Habitat Fragmentation on Native Bee Assemblages in a California Biodiversity Hotspot. *Insects*, *12*(2), 135. https://doi.org/10.3390/insects12020135

Hutchinson, L. A., Oliver, T. H., Breeze, T. D. [Tom D.], O'Connor, R. S. [Rory S.], Potts, S. G. [Simon G.], Roberts, S. P. M., & Garratt, M. P. D. [Michael P. D.] (2022). Inventorying and monitoring crop pollinating bees: Evaluating the effectiveness of common sampling methods. *Insect Conservation and Diversity*, *15*(3), 299–311. https://doi.org/10.1111/icad.12557

Ikemoto, M., Kuramitsu, K., Sueyoshi, M., Seguchi, S., & Yokoi, T. (2021). Relative trapping efficiencies of different types of attraction traps for three insect orders in an agricultural field. *Applied Entomology and Zoology*, *56*(3), 393–405. https://doi.org/10.1007/s13355-021-00748-4

Ikemoto, M., & Yokoi, T. (2021). A test of new trapping methods for honey bees using odor attractants and a dry trap. *Journal of Apicultural Research.* Advance online publication. https://doi.org/10.1080/00218839.2021.1944569

Ishay, J. S., Plotkin, M., Ermakov, N. Y., Volynchik, S., Barkay, Z., & Bergman, D. J. (2006). The thermogenic center in social wasps. *Journal of Electron Microscopy*, *55*(1), 41–49. https://doi.org/10.1093/jmicro/dfl002

Jackson, M. M., Turner, M. G., & Pearson, S. M. (2014). Logging Legacies Affect Insect Pollinator Communities in Southern Appalachian Forests. *Southeastern Naturalist*, *13*(2), 317–336. https://doi.org/10.1656/058.013.0213

Johansson, V., Gustafsson, L., Andersson, P., & Hylander, K. (2020). Fewer butterflies and a different composition of bees, wasps and hoverflies on recently burned compared to unburned clear-cuts, regardless of burn severity. *Forest Ecology and Management*, *463*, 118033. https://doi.org/10.1016/j.foreco.2020.118033

Johnson, I. M., Edwards, T. J., & Johnson, S. D. (2021). Geographical Variation in Flower Color in the Grassland Daisy Gerbera aurantiaca: Testing for Associations With Pollinators and Abiotic Factors. *Frontiers in Ecology and Evolution*, *9*, 676520. https://doi.org/10.3389/fevo.2021.676520

Joseph, S. V., Harris-Shultz, K., & Jespersen, D. (2020). Evidence of Pollinators Foraging on Centipedegrass Inflorescences. *Insects*, *11*(11), 795. https://doi.org/10.3390/insects11110795

Joseph, S. V., Harris-Shultz, K., Jespersen, D., Vermeer, B., & Julian, C. (2020). Incidence and Abundance of Bees and Wasps (Hymenoptera) in Centipedegrass Lawns in Georgia. *Journal of Entomological Science*, *55*(4), 547–559. https://doi.org/10.18474/0749-8004-55.4.547

Joshi, N. K., Biddinger, D. J., Fleischer, S., & Passoa, S. (2013). First Report of the Adventive Species Sitochroa palealis (Lepidoptera: Crambidae) in Pennsylvania and its Attraction to the Sex Pheromone of the European Corn Borer, Ostrinia nubilalis (Lepidoptera: Crambidae). *The Great Lakes Entomologist*, *46*(1-2), 99–103.

Joshi, N. K., Leslie, T., Rajotte, E. G., Kammerer, M. A., Otieno, M., & Biddinger, D. J. (2015). Comparative Trapping Efficiency to Characterize Bee Abundance, Diversity, and Community Composition in Apple Orchards. *Annals of the Entomological Society of America*, *108*(5), 785–799. https://doi.org/10.1093/aesa/sav057

Kammerer, M., Tooker, J. F., & Grozinger, C. M. (2020). A long-term dataset on wild bee abundance in Mid-Atlantic United States. *Scientific Data*, *7*(1), 240. https://doi.org/10.1038/s41597-020-00577-0

Kauffman, K. L., Elmore, R. D., Davis, C. A., Fuhlendorf, S. D., Goodman, L. E., Hagen, C. A., & Tanner, E. P. (2021). Role of the thermal environment in scaled quail (Callipepla squamata) nest site selection and survival. *Journal of Thermal Biology*, *95*, 102791. https://doi.org/10.1016/j.jtherbio.2020.102791

Kehinde, T., Wehrden, H. von, Samways, M., Klein, A.‑M. [Alexandra-Maria], & Brittain, C. (2018). Organic farming promotes bee abundance in vineyards in Italy but not in South Africa. *Journal of Insect Conservation*, *22*(1), 61–67. https://doi.org/10.1007/s10841-017-0038-4

Knapp, M., González, E., Štrobl, M., Seidl, M., Jakubíková, L., Čížek, O., . . . Kadlec, T. (2022). Artificial field defects: A low-cost measure to support arthropod diversity in arable fields. *Agriculture, Ecosystems & Environment*, *325*, 107748. https://doi.org/10.1016/j.agee.2021.107748

Kohler, M., Sturm, A., Sheffield, C. S. [Cory S.], Carlyle, C. N., & Manson, J. S. (2020). Native bee communities vary across three prairie ecoregions due to land use, climate, sampling method and bee life history traits. *Insect Conservation and Diversity*, *13*(6), 571–584. https://doi.org/10.1111/icad.12427

Kovac, H., & Stabentheiner, A. (1999). Effect of food quality on the body temperature of wasps (Paravespula vulgaris). *Journal of Insect Physiology*, *45*(2), 183–190. https://doi.org/10.1016/S0022-1910(98)00115-2

Kovacic, M., Puskadija, Z., Ozimec, S., Majic, I., & Sarajlic, A. (2016). Importance of pollinating insects for maintaining sustainable agriculture in eastern Croatia. *Journal of Environmental Protection and Ecology*, *17*(4), 1408–1415.

Kovács-Hostyánszki, A., Haenke, S., Batáry, P., Jauker, B., Báldi, A., Tscharntke, T., & Holzschuh, A. (2013). Contrasting effects of mass-flowering crops on bee pollination of hedge plants at different spatial and temporal scales. *Ecological Applications*, *23*(8), 1938–1946. https://doi.org/10.1890/12-2012.1

Kovács-Hostyánszki, A., Soltész, Z., Szigeti, V., Somay, L., & Báldi, A. (2021). Non-rotational set-aside fields improve reproductive success of cavity-nesting bees and wasps at the landscape scale, but have no effect on other wild bees and hoverflies in mid-summer. *Agriculture, Ecosystems & Environment*, *308*, 107255. https://doi.org/10.1016/j.agee.2020.107255

Kozuharova, E. (2018). Flower constancy of bumblebees – the case of Onobrychis pindicola (Fabaceae) pollinators. *Journal of Apicultural Science*, *62*(1), 135–140. https://doi.org/10.2478/jas-2018-0005

Kozuharova, E. K. (2018). Functional flower morphology and entomophilous pollination syndromes in Cape Kaliakra Nature Reserve (North Black Sea coast, Bulgaria). *Acta Zoologica Bulgarica*, *Suppl. 11*, 87–90.

Krahner, A., Schmidt, J., Maixner, M., Porten, M., & Schmitt, T. (2021). Evaluation of four different methods for assessing bee diversity as ecological indicators of agro-ecosystems. *Ecological Indicators*, *125*, 107573. https://doi.org/10.1016/j.ecolind.2021.107573

Krewenka, K. M., Holzschuh, A., Tscharntke, T., & Dormann, C. F. (2011). Landscape elements as potential barriers and corridors for bees, wasps and parasitoids. *Biological Conservation*, *144*(6), 1816–1825. https://doi.org/10.1016/j.biocon.2011.03.014

Ksiazek, K., Fant, J., & Skogen, K. (2012). An assessment of pollen limitation on Chicago green roofs. *Landscape and Urban Planning*, *107*(4), 401–408. https://doi.org/10.1016/j.landurbplan.2012.07.008

Kuhlman, M., & Burrows, S. (2017). Checklist of bees (Apoidea) from a private conservation property in west-central Montana. *Biodiversity Data Journal*, *5*, e11506. https://doi.org/10.3897/BDJ.5.e11506

Kumari, R., Singh, K., Singh, R., Bhatia, N., & Nain, M. S. (2019). Development of healthy ready-to-eat (RTE) breakfast cereal from popped pearl millet. *Indian Journal of Agricultural Sciences*, *89*(5), 877–881.

Kwaiser, K. S., & Hendrix, S. D. (2008). Diversity and abundance of bees (Hymenoptera : Apiformes) in native and ruderal grasslands of agriculturally dominated landscapes. *Agriculture, Ecosystems & Environment*, *124*(3-4), 200–204. https://doi.org/10.1016/j.agee.2007.09.012

Ladd, P. G., Yates, C. J., Dillon, R., & Palmer, R. (2019). Pollination ecology of Tetratheca species from isolated, arid habitats (Banded Iron Formations) in Western Australia. *Australian Journal of Botany*, *67*(3), 248–255. https://doi.org/10.1071/BT18249

Lagucki, E., Burdine, J. D., & McCluney, K. E. (2017). Urbanization alters communities of flying arthropods in parks and gardens of a medium-sized city. *PeerJ*, *5*, e3620. https://doi.org/10.7717/peerj.3620

Lamprecht, I., Maierhofer, C., & Röllig, M. (2006). A thermographic promenade through the Berlin Botanic Garden. *Thermochimica Acta*, *446*(1-2), 4–10. https://doi.org/10.1016/j.tca.2006.02.039

Lapuente, J., Hicks, T. C., & Linsenmair, K. E. (2017). Fluid dipping technology of chimpanzees in Comoé National Park, Ivory Coast. *American Journal of Primatology*, *79*(5), e22628. https://doi.org/10.1002/ajp.22628

Larkin, M., & Stanley, D. A. (2021). Impacts of management at a local and landscape scale on pollinators in semi‐natural grasslands. *Journal of Applied Ecology*, *58*(11), 2505–2514. https://doi.org/10.1111/1365-2664.13990

Larsen, N. J., Minor, M. A., Cruickshank, R. H., & Robertson, A. W. (2014). Optimising methods for collecting Hymenoptera, including parasitoids and Halictidae bees, in New Zealand apple orchards. *Journal of Asia-Pacific Entomology*, *17*(3), 375–381. https://doi.org/10.1016/j.aspen.2014.03.004

Lasway, J. V., Kinabo, N. R., Mremi, R. F., Martin, E. H., Nyakunga, O. C., Sanya, J. J., . . . Njovu, H. K. (2021). A synopsis of the Bee occurrence data of northern Tanzania. *Biodiversity Data Journal*, *9*, e68190. https://doi.org/10.3897/BDJ.9.e68190

Lazarina, M., Sgardelis, S. P., Tscheulin, T., Kallimanis, A. S., Devalez, J., & Petanidou, T. (2016). Bee response to fire regimes in Mediterranean pine forests: The role of nesting preference, trophic specialization, and body size. *Basic and Applied Ecology*, *17*(4), 308–320. https://doi.org/10.1016/j.baae.2016.02.001

Le Féon, V., Burel, F., Chifflet, R., Henry, M., Ricroch, A., Vaissière, B. E., & Baudry, J. (2013). Solitary bee abundance and species richness in dynamic agricultural landscapes. *Agriculture, Ecosystems & Environment*, *166*, 94–101. https://doi.org/10.1016/j.agee.2011.06.020

Le Féon, V., Poggio, S. L., Torretta, J. P., Bertrand, C., Molina, G. A. R., Burel, F., . . . Ghersa, C. M. (2016). Diversity and life-history traits of wild bees (Insecta: Hymenoptera) in intensive agricultural landscapes in the Rolling Pampa, Argentina. *Journal of Natural History*, *50*(19-20), 1175–1196. https://doi.org/10.1080/00222933.2015.1113315

LeCroy, K. A., Savoy-Burke, G., Carr, D. E., Delaney, D. A., & Roulston, T. H. (2020). Decline of six native mason bee species following the arrival of an exotic congener. *Scientific Reports*, *10*(1), 18745. https://doi.org/10.1038/s41598-020-75566-9

Leguizamón, Y., Debandi, G., & Vázquez, D. P. (2021). Managed honeybee hives and the diversity of wild bees in a dryland nature reserve. *Apidologie*, *52*(6), 991–1001. https://doi.org/10.1007/s13592-021-00882-6

Leong, J. M., & Thorp, R. W. (1999). Colour-coded sampling: The pan trap colour preferences of oligolectic and nonoligolectic bees associated with a vernal pool plant. *Ecological Entomology*, *24*(3), 329–335. https://doi.org/10.1046/j.1365-2311.1999.00196.x

Li, H., Orr, M. C., Luo, A., Dou, F., Kou, R., Hu, F., . . . Huang, D. (2021). Relationships between wild bee abundance and fruit set of Camellia oleifera Abel. *Journal of Applied Entomology*, *145*(4), 277–285. https://doi.org/10.1111/jen.12849

Li, J.‑K., & Huang, S.‑Q. (2009). Effective pollinators of Asian sacred lotus (Nelumbo nucifera): Contemporary pollinators may not reflect the historical pollination syndrome. *Annals of Botany*, *104*(5), 845–851. https://doi.org/10.1093/aob/mcp173

Longcore, T., Aldern, H. L., Eggers, J. F., Flores, S., Franco, L., Hirshfield-Yamanishi, E., . . . Barroso, A. M. (2015). Tuning the white light spectrum of light emitting diode lamps to reduce attraction of nocturnal arthropods. *Philosophical Transactions of the Royal Society B-Biological Sciences*, *370*(1667). https://doi.org/10.1098/rstb.2014.0125

Lopes, A. V., & Machado, I. C. (1998). Floral biology and reproductive ecology of Clusia nemorosa (Clusiaceae) in northeastern Brazil. *Plant Systematics and Evolution*, *213*(1-2), 71–90. https://doi.org/10.1007/BF00988909

Lorandi, S., Mustin, K., Halinski, R. [Rosana], & Iserhard, C. A. (2023). Are there differences in the diversity of bees between organic and conventional agroecosystems in the Pampa biome? *Journal of Apicultural Research*, *62*(2), 250–262. https://doi.org/10.1080/00218839.2021.1888524

Love, B. G., & Cane, J. H. (2016). Limited direct effects of a massive wildfire on its sagebrush steppe bee community. *Ecological Entomology*, *41*(3), 317–326. https://doi.org/10.1111/een.12304

Lowenstein, D. M., Huseth, A. S., & Groves, R. L. (2012). Response of Wild Bees (Hymenoptera: Apoidea: Anthophila) to Surrounding Land Cover in Wisconsin Pickling Cucumber. *Environmental Entomology*, *41*(3), 532–540. https://doi.org/10.1603/EN11241

Lozada-Gobilard, S., Landivar Albis, C. M., Rupik, K. B., Pätzig, M., Hausmann, S., Tiedemann, R., & Joshi, J. (2021). Habitat quality and connectivity in kettle holes enhance bee diversity in agricultural landscapes. *Agriculture, Ecosystems & Environment*, *319*, 107525. https://doi.org/10.1016/j.agee.2021.107525

Lucas, A., Bull, J. C., Vere, N. de, Neyland, P. J., & Forman, D. W. (2017). Flower resource and land management drives hoverfly communities and bee abundance in seminatural and agricultural grasslands. *Ecology and Evolution*, *7*(19), 8073–8086. https://doi.org/10.1002/ece3.3303

Lundin, O., Rundlöf, M., Smith, H. G., & Bommarco, R. (2012). Towards integrated pest management in red clover seed production. *Journal of Economic Entomology*, *105*(5), 1620–1628. https://doi.org/10.1603/EC12179

Maia, U. M., Pinto, C. E., Miranda, L. S., Coelho, B. W. T. [Beatriz W. T.], Santos Junior, J. E., Raiol, R. L., . . . Giannini, T. C. (2020). Forest Matrix Fosters High Similarity in Bee Composition Occurring on Isolated Outcrops Within Amazon Biome. *Environmental Entomology*, *49*(6), 1374–1382. https://doi.org/10.1093/ee/nvaa115

Marshall, L., Carvalheiro, L. G., Aguirre-Gutiérrez, J., Bos, M., Groot, G. A. de, Kleijn, D., . . . Biesmeijer, J. C. (2015). Testing projected wild bee distributions in agricultural habitats: Predictive power depends on species traits and habitat type. *Ecology and Evolution*, *5*(19), 4426–4436. https://doi.org/10.1002/ece3.1579

Martínez-Harms, J., Vorobyev, M., Schorn, J., Shmida, A., Keasar, T., Homberg, U., . . . Menzel, R. (2012). Evidence of red sensitive photoreceptors in Pygopleurus israelitus (Glaphyridae: Coleoptera) and its implications for beetle pollination in the southeast Mediterranean. *Journal of Comparative Physiology a*, *198*(6), 451–463. https://doi.org/10.1007/s00359-012-0722-5

McCravy, K. W. (2018). A Review of Sampling and Monitoring Methods for Beneficial Arthropods in Agroecosystems. *Insects*, *9*(4), 170. https://doi.org/10.3390/insects9040170

McCravy, K. W., Geroff, R. K., & Gibbs, J. [Jason] (2016). Malaise trap sampling efficiency for bees (Hymenoptera: Apoidea) in a restored tallgrass prairie. *Florida Entomologist*, *99*(2), 321–323. https://doi.org/10.1653/024.099.0230

McCravy, K. W., Geroff, R. K., & Gibbs, J. [Jason] (2019). Bee (Hymenoptera: Apoidea: Anthophila) functional traits in relation to sampling methodology in a restored tallgrass prairie. *Florida Entomologist*, *102*(1), 134–140. https://doi.org/10.1653/024.102.0122

McCravy, K. W., & Ruholl, J. D. (2017). Bee (Hymenoptera: Apoidea) Diversity and Sampling Methodology in a Midwestern USA Deciduous Forest. *Insects*, *8*(3), 81. https://doi.org/10.3390/insects8030081

McCune, F., Normandin, É., Mazerolle, M. J., & Fournier, V. [Valérie] (2020). Response of wild bee communities to beekeeping, urbanization, and flower availability. *Urban Ecosystems*, *23*(1), 39–54. https://doi.org/10.1007/s11252-019-00909-y

McKechnie, I. M., Thomsen, C. J. M., & Sargent, R. D. (2017). Forested field edges support a greater diversity of wild pollinators in lowbush blueberry (Vaccinium angustifolium). *Agriculture, Ecosystems & Environment*, *237*, 154–161. https://doi.org/10.1016/j.agee.2016.12.005

McNeil, D. J., Otto, C. R. V., Moser, E. L., Urban-Mead, K. R., King, D. E., Rodewald, A. D., & Larkin, J. L. (2019). Distance models as a tool for modelling detection probability and density of native bumblebees. *Journal of Applied Entomology*, *143*(3), 225–235. https://doi.org/10.1111/jen.12583

Meagher, Robert L., Jr., Watrous, K. M., Fleischer, S. J., Nagoshi, R. N., Brown, J. T., & Westbrook, J. K. (2020). Comparison of bee composition in sunn hemp and other cover crops. *Florida Entomologist*, *103*(4), 419–424. https://doi.org/10.1653/024.103.00401

Meindl, G. A., & Ashman, T.‑L. (2015). Effects of floral metal accumulation on floral visitor communities: Introducing the elemental filter hypothesis. *American Journal of Botany*, *102*(3), 379–389. https://doi.org/10.3732/ajb.1400468

Mendes, M. F., Gottschalk, M. S., Halinski, R. [Rosana], Moreira, H. R., Dalmorra, C., & Valente-Gaiesky, V. L. S. (2021). First report of colored pan traps to capture Drosophilidae (Diptera). *Revista Brasileira De Entomologia*, *65*(3), e20210057. https://doi.org/10.1590/1806-9665-RBENT-2021-0057

Mentone, T. O., Morini, M. S. C., Souza, L. [L.], & Braga, S. M. P. (2009). Hymenoptera Communities in an Agroecosystem Using Direct Seeding in Southeastern Brazil. *Sociobiology*, *53*(2B), 473–486.

Methven, L., Langreney, E., & Prescott, J. (2012). Changes in liking for a no added salt soup as a function of exposure. *Food Quality and Preference*, *26*(2), 135–140. https://doi.org/10.1016/j.foodqual.2012.04.012

Meyer, S., Unternährer, D., Arlettaz, R., Humbert, J.‑Y., & Menz, M. H. (2017). Promoting diverse communities of wild bees and hoverflies requires a landscape approach to managing meadows. *Agriculture, Ecosystems & Environment*, *239*, 376–384. https://doi.org/10.1016/j.agee.2017.01.037

Middelkoop, A., van Marwijk, M. A., Kemp, B., & Bolhuis, J. E. (2019). Pigs Like It Varied; Feeding Behavior and Pre- and Post-weaning Performance of Piglets Exposed to Dietary Diversity and Feed Hidden in Substrate During Lactation. *Frontiers in Veterinary Science*, *6*, 408. https://doi.org/10.3389/fvets.2019.00408

Miguez, M. B., & Amela García, M. T. (2019). Breeding system, floral biology and pollinators of Sagittaria montevidensis subsp. Montevidensis Cham. Et Schlech. (Alismataceae). *Aquatic Botany*, *158*, 103123. https://doi.org/10.1016/j.aquabot.2019.06.002

Milam, J. C., Litvaitis, J. A., Warren, A., Keirstead, D., & King, D. I. (2018). Bee Assemblages in Managed Early-successional Habitats in Southeastern New Hampshire. *Northeastern Naturalist*, *25*(3), 437–459. https://doi.org/10.1656/045.025.0309

Milberg, P., Eriksson, V., & Bergman, K.‑O. (2021). Assemblages of flower-visiting insects in clear-cuts are rich and dynamic. *European Journal of Entomology*, *118*, 182–191. https://doi.org/10.14411/eje.2021.019

Miljanic, A. S., Loy, X., Gruenewald, D. L., Dobbs, E. K., Gottlieb, I. G. W., Fletcher, Robert J., Jr., & Brosi, B. J. (2019). Bee communities in forestry production landscapes: Interactive effects of local-level management and landscape context. *Landscape Ecology*, *34*(5), 1015–1032. https://doi.org/10.1007/s10980-018-0651-y

Miller, A. E., Brosi, B. J., Magnacca, K., Daily, G. C., & Pejchar, L. (2015). Pollen Carried By Native and Nonnative Bees in the Large-scale Reforestation of Pastureland in Hawai'i: Implications for Pollination. *Pacific Science*, *69*(1), 67–79. https://doi.org/10.2984/69.1.5

Minachilis, K., Kantsa, A., Devalez, J., Trigas, P., Tscheulin, T., & Petanidou, T. (2020). Bumblebee diversity and pollination networks along the elevation gradient of Mount Olympus, Greece. *Diversity and Distributions*, *26*(11), 1566–1581. https://doi.org/10.1111/ddi.13138

Moisan-DeSerres, J., Chagnon, M., & Fournier, V. [Valérie] (2015). Influence of windbreaks and forest borders on abundance and species richness of native pollinators in lowbush blueberry fields in Québec, Canada. *The Canadian Entomologist*, *147*(4), 432–442. https://doi.org/10.4039/tce.2014.55

Montgomery, G. A., Belitz, M. W., Guralnick, R. P., & Tingley, M. W. (2021). Standards and Best Practices for Monitoring and Benchmarking Insects. *Frontiers in Ecology and Evolution*, *8.* https://doi.org/10.3389/fevo.2020.579193

Montoya, A. L., Parra, J. L., & Wolff, M. (2021). Structure and diversity of hoverflies (Diptera: Syrphidae) in northwestern Colombian Paramos: Towards the identification of bioindicator species in the Tropical Andes. *Journal of Insect Conservation*, *25*(5-6), 809–828. https://doi.org/10.1007/s10841-021-00346-3

Morandin, L. A., & Kremen, C. (2013). Hedgerow restoration promotes pollinator populations and exports native bees to adjacent fields. *Ecological Applications*, *23*(4), 829–839. https://doi.org/10.1890/12-1051.1

Morandin, L. A., & Winston, M. L. (2005). Wild bee abundance and seed production in conventional, organic and genetically modified canola. *Ecological Applications*, *15*(3), 871–881. https://doi.org/10.1890/03-5271

Moreira, E. F., Da Santos, R. L. S., Penna, U. L., Angel-Coca, C., Oliveira, F. F. de, & Viana, B. F. [Blandina Felipe] (2016). Are pan traps colors complementary to sample community of potential pollinator insects? *Journal of Insect Conservation*, *20*(4), 583–596. https://doi.org/10.1007/s10841-016-9890-x

Moroń, D., Szentgyörgyi, H., Wantuch, M., Celary, W., Westphal, C., Settele, J., & Woyciechowski, M. (2008). Diversity of wild bees in wet meadows: Implications for conservation. *Wetlands*, *28*(4), 975–983. https://doi.org/10.1672/08-83.1

Morrison, J., Izquierdo, J., Plaza, E. H., & González-Andújar, J. L. (2017). The role of field margins in supporting wild bees in Mediterranean cereal agroecosystems: Which biotic and abiotic factors are important? *Agriculture, Ecosystems & Environment*, *247*, 216–224. https://doi.org/10.1016/j.agee.2017.06.047

Motyka, E., Wiśniowski, B., & Szczepko, K. (2016). The Wild Bees Andrena gallica Schmiedeknecht, 1883 and Andrena assimilis Radoszkowski, 1876 (Apoidea: Andrenidae) in Poland. *Journal of Apicultural Science*, *60*(2), 111–118. https://doi.org/10.1515/JAS-2016-0022

Moylett, H., Youngsteadt, E., & Sorenson, C. (2020). The Impact of Prescribed Burning on Native Bee Communities (Hymenoptera: Apoidea: Anthophila) in Longleaf Pine Savannas in the North Carolina Sandhills. *Environmental Entomology*, *49*(1), 211–219. https://doi.org/10.1093/ee/nvz156

Mpondo, F. T., Ndakidemi, P. A., Pauly, A., & Treydte, A. C. (2021). Traditional rangeland management can conserve insect pollinators in a semi-arid rangeland, northern Tanzania. *Acta Oecologica*, *113*, 103790. https://doi.org/10.1016/j.actao.2021.103790

Munyuli, T. M. B. (2012). Is pan-trapping the most reliable sampling method for measuring and monitoring bee biodiversity in agroforestry systems in sub-Saharan Africa? *International Journal of Tropical Insect Science*, *33*(01), 14–37. https://doi.org/10.1017/S1742758412000379

Nachev, V., Stich, K. P., & Winter, Y. (2013). Weber's Law, the Magnitude Effect and Discrimination of Sugar Concentrations in Nectar-Feeding Animals. *PLOS ONE*, *8*(9), e74144. https://doi.org/10.1371/journal.pone.0074144

Neame, L. A., Griswold, T., & Elle, E. (2013). Pollinator nesting guilds respond differently to urban habitat fragmentation in an oak-savannah ecosystem. *Insect Conservation and Diversity*, *6*(1), 57–66. https://doi.org/10.1111/j.1752-4598.2012.00187.x

Nelson, C. J., Frost, C. M., & Nielsen, S. E. (2021). Narrow anthropogenic linear corridors increase the abundance, diversity, and movement of bees in boreal forests. *Forest Ecology and Management*, *489*, 119044. https://doi.org/10.1016/j.foreco.2021.119044

Neves Jr., C. L., Correa Barros, H., Rodrigues Diniz, M., Freire Correia, B. E., Chaves Ferreira, L. A., Guimarães Silva, A., . . . Correa Rêgo, M. M. (2021). Bees from an Island in the Delta of the Americas (Maranhão state, Brazil) and their Floristic Interactions. *Sociobiology*, *68*(3), e5783. https://doi.org/10.13102/sociobiology.v68i3.5783

Ngo, H. T. [H. T.], Gibbs, J. [J.], Griswold, T. [T.], & Packer, L. (2013). Evaluating bee (Hymenoptera: Apoidea) diversity using Malaise traps in coffee landscapes of Costa Rica. *The Canadian Entomologist*, *145*(4), 435–453. https://doi.org/10.4039/tce.2013.16

Nielsen, A., Steffan-Dewenter, I., Westphal, C., Messinger, O., Potts, S. G. [Simon G.], Roberts, S. P. M., . . . Petanidou, T. (2011). Assessing bee species richness in two Mediterranean communities: Importance of habitat type and sampling techniques. *Ecological Research*, *26*(5), 969–983. https://doi.org/10.1007/s11284-011-0852-1

Noel, G., Bonnet, J., Everaerts, S., Danel, A., Calderan, A., Liedekerke, A. de, . . . Serteyn, L. (2021). Distribution of wild bee (Hymenoptera: Anthophila) and hoverfly (Diptera: Syrphidae) communities within farms undergoing ecological transition. *Biodiversity Data Journal*, *9*, e60665. https://doi.org/10.3897/BDJ.9.e60665

Nol, E., Douglas, H., & Crins, W. J. (2006). Responses of Syrphids, Elaterids and Bees to Single-tree Selection Harvesting in Algonquin Provincial Park, Ontario. *Canadian Field-Naturalist*, *120*(1), 15–21. https://doi.org/10.22621/cfn.v120i1.239

Nooten, S. S., Odanaka, K. A., & Rehan, S. M. [Sandra M.] (2020). Effects of Farmland and Seasonal Phenology on Wild Bees in Blueberry Orchards. *Northeastern Naturalist*, *27*(4), 841–860. https://doi.org/10.1656/045.027.0420

Normandin, É., Vereecken, N. J., Buddle, C. M., & Fournier, V. [Valérie] (2017). Taxonomic and functional trait diversity of wild bees in different urban settings. *PeerJ*, *5*, e3051. https://doi.org/10.7717/peerj.3051

Nuttman, C. V., Otieno, M., Kwapong, P. K., Combey, R., Willmer, P., & Potts, S. G. [Simon G.] (2011). The Utility of Aerial Pan-Trapping for Assessing Insect Pollinators Across Vertical Strata. *Journal of the Kansas Entomological Society*, *84*(4), 260–270. https://doi.org/10.2317/JKES110319.1

O'Connor, R. S. [Rory S.], Kunin, W. E., Garratt, M. P. D. [Michael P. D.], Potts, S. G. [Simon G.], Roy, H. E., Andrews, C., . . . Carvell, C. [Claire] (2019). Monitoring insect pollinators and flower visitation: The effectiveness and feasibility of different survey methods. *Methods in Ecology and Evolution*, *10*(12), 2129–2140. https://doi.org/10.1111/2041-210X.13292

Odanaka, K., Gibbs, J. [Jason], Turley, N. E., Isaacs, R., & Brudvig, L. A. (2020). Canopy thinning, not agricultural history, determines early responses of wild bees to longleaf pine savanna restoration. *Restoration Ecology*, *28*(1), 138–146. https://doi.org/10.1111/rec.13043

Olson, D. M., Gibbs, J. [Jason], & Schmidt, J. M. (2021). Wild bee pollinators foraging in peanut and cotton adjacent to native wildflower strips. *Florida Entomologist*, *104*(3), 165–172. https://doi.org/10.1653/024.104.0304

Olynyk, M., Westwood, A. R., & Koper, N. (2021). Effects of Natural Habitat Loss and Edge Effects on Wild Bees and Pollination Services in Remnant Prairies. *Environmental Entomology*, *50*(3), 732–743. https://doi.org/10.1093/ee/nvaa186

Osterman, J., Theodorou, P., Radzevičiūtė, R., Schnitker, P., & Paxton, R. J. (2021). Apple pollination is ensured by wild bees when honey bees are drawn away from orchards by a mass co-flowering crop, oilseed rape. *Agriculture, Ecosystems & Environment*, *315*, 107383. https://doi.org/10.1016/j.agee.2021.107383

Padrón, P. S., Vásquez, C. B., Durán, S. C., Pezo, K. V., Loyola, N. A., & Junghanns, A. (2021). Use of colored pan traps method for monitoring insect (Diptera and Hymenoptera) diversity in the Southern Tropical Andes of Ecuador. *International Journal of Tropical Insect Science*, *41*(1), 643–652. https://doi.org/10.1007/s42690-020-00252-2

Pardee, G. L., & Philpott, S. M. [Stacy M.] (2014). Native plants are the bee's knees: Local and landscape predictors of bee richness and abundance in backyard gardens. *Urban Ecosystems*, *17*(3), 641–659. https://doi.org/10.1007/s11252-014-0349-0

Parys, K. A., Esquivel, I. L., Wright, K. W., Griswold, T., & Brewer, M. J. (2020). Native Pollinators (Hymenoptera: Anthophila) in Cotton Grown in the Gulf South, United States. *Agronomy*, *10*(5), 698. https://doi.org/10.3390/agronomy10050698

Pascarella, J. (2017). Range extension of the introduced bee species Euglossa dilemma (Hymenoptera: Apidae) in Monroe County, Florida, with notes of additional range extensions in southern Florida. *Florida Entomologist*, *100*(1), 209–210. https://doi.org/10.1653/024.100.0137

Pei, C. K., Hovick, T. J., Duquette, C. A., Limb, R. F., Harmon, J. P., & Geaumont, B. A. (2022). Two common bee-sampling methods reflect different assemblages of the bee (Hymenoptera: Apoidea) community in mixed-grass prairie systems and are dependent on surrounding floral resource availability. *Journal of Insect Conservation*, *26*(1), 69–83. https://doi.org/10.1007/s10841-021-00362-3

Peng, S., & Deng, W. (2014). A facile approach for preparing biomimetic polymer macroporous structures with petal or lotus effects. *New Journal of Chemistry*, *38*(3), 1011–1018. https://doi.org/10.1039/c3nj01156a

Perillo, L. N., Neves, F. d. S., Antonini, Y., & Martins, R. P. (2017). Compositional changes in bee and wasp communities along Neotropical mountain altitudinal gradient. *PLOS ONE*, *12*(7), e0182054. https://doi.org/10.1371/journal.pone.0182054

Perrot, T., Gaba, S., Roncoroni, M., Gautier, J.‑L., & Bretagnolle, V. (2018). Bees increase oilseed rape yield under real field conditions. *Agriculture, Ecosystems & Environment*, *266*, 39–48. https://doi.org/10.1016/j.agee.2018.07.020

Pfiffner, L., Ostermaier, M., Stoeckli, S., & Müller, A. (2018). Wild bees respond complementarily to ‘high-quality’ perennial and annual habitats of organic farms in a complex landscape. *Journal of Insect Conservation*, *22*(3-4), 551–562. https://doi.org/10.1007/s10841-018-0084-6

Phillips, B. B., Gaston, K. J., Bullock, J. M., & Osborne, J. L. (2019). Road verges support pollinators in agricultural landscapes, but are diminished by heavy traffic and summer cutting. *Journal of Applied Ecology*, *56*(10), 2316–2327. https://doi.org/10.1111/1365-2664.13470

Pisanty, G., Scheuchl, E., & Dorchin, N. (2016). Eight new species of Andrena Fabricius (Hymenoptera: Apoidea: Andrenidae) from Israel-a Mediterranean hotspot for wild bees. *Zootaxa*, *4189*(3), 485–515. https://doi.org/10.11646/zootaxa.4189.3.3

Plascencia, M., & Philpott, S. M. [S. M.] (2017). Floral abundance, richness, and spatial distribution drive urban garden bee communities. *Bulletin of Entomological Research*, *107*(5), 658–667. https://doi.org/10.1017/S0007485317000153

Popic, T. J., Davila, Y. C., & Wardle, G. M. (2013). Evaluation of Common Methods for Sampling Invertebrate Pollinator Assemblages: Net Sampling Out-Perform Pan Traps. *PLOS ONE*, *8*(6), e66665. https://doi.org/10.1371/journal.pone.0066665

Portman, Z. M., Bruninga-Socolar, B., & Cariveau, D. P. (2020). The State of Bee Monitoring in the United States: A Call to Refocus Away From Bowl Traps and Towards More Effective Methods. *Annals of the Entomological Society of America*, *113*(5), 337–342. https://doi.org/10.1093/aesa/saaa010

Prado, S. G., Ngo, H. T. [Hien T.], Florez, J. A., & Collazo, J. A. (2017). Sampling bees in tropical forests and agroecosystems: A review. *Journal of Insect Conservation*, *21*(5-6), 753–770. https://doi.org/10.1007/s10841-017-0018-8

Prendergast, K. S., & Hogendoorn, K. (2021). Forum: Methodological shortcomings and lack of taxonomic effort beleaguer Australian bee studies. *Austral Ecology*, *46*(5), 880–884. https://doi.org/10.1111/aec.12998

Prendergast, K. S., Menz, M. H. M., Dixon, K. W., & Bateman, P. W. (2020). The relative performance of sampling methods for native bees: An empirical test and review of the literature. *Ecosphere*, *11*(5), e03076. https://doi.org/10.1002/ecs2.3076

Proesmans, W., Bonte, D., Smagghe, G., Meeus, I., Decocq, G., Spicher, F., . . . Verheyen, K. (2019). Small forest patches as pollinator habitat: Oases in an agricultural desert? *Landscape Ecology*, *34*(3), 487–501. https://doi.org/10.1007/s10980-019-00782-2

Quistberg, R. D., Bichier, P., & Philpott, S. M. [Stacy M.] (2016). Landscape and Local Correlates of Bee Abundance and Species Richness in Urban Gardens. *Environmental Entomology*, *45*(3), 592–601. https://doi.org/10.1093/ee/nvw025

Ramello, P. J., Álvarez, L. J., Almada, V., & Lucia, M. (2021). The melittofauna and its floral associations in a natural riparian forest in Buenos Aires province, Argentina. *Journal of Apicultural Research*, *60*(2), 241–254. https://doi.org/10.1080/00218839.2020.1765489

Ramírez-Freire, L., José Alanís-Flores, G., Ayala-Barajas, R., Quiroz-Martínez, H., & Gerardo Velazco-Macías, C. (2012). Bees of the genus Agapostemon (Hymenoptera: Halictidae) of the state of Nuevo León, Mexico. *Revista Mexicana de Biodiversidad*, *83*(1), 63–72.

Rehan, S. M. [Sandra M.], & Richards, M. H. [Miriam H.] (2010). Nesting biology and subsociality in Ceratina calcarata (Hymenoptera: Apidae). *The Canadian Entomologist*, *142*(1), 65–74. https://doi.org/10.4039/n09-056

Rhoades, P., Griswold, T., Waits, L., Bosque-Pérez, N. A. [Nilsa A.], Kennedy, C. M., & Eigenbrode, S. D. (2017). Sampling technique affects detection of habitat factors influencing wild bee communities. *Journal of Insect Conservation*, *21*(4), 703–714. https://doi.org/10.1007/s10841-017-0013-0

Richards, M. H. [M. H.], Rutgers-Kelly, A., Gibbs, J. [J.], Vickruck, J. L., Rehan, S. M. [S. M.], & Sheffield, C. S. [C. S.] (2011). Bee diversity in naturalizing patches of Carolinian grasslands in southern Ontario, Canada. *The Canadian Entomologist*, *143*(3), 279–299. https://doi.org/10.4039/n11-010

Richards, M. H. [M. H.], Vickruck, J. L., & Rehan, S. M. [S. M.] (2010). Colony Social Organisation of Halictus confusus in Southern Ontario, with Comments on Sociality in the Subgenus H. (Seladonia). *Journal of Hymenoptera Research*, *19*(1), 144–158.

Richards, M. H. [Miriam H.], Onuferko, T. M., & Rehan, S. M. [Sandra M.] (2015). Phenological, but not social, variation associated with climate differences in a eusocial sweat bee, Halictus ligatus, nesting in southern Ontario. *Journal of Hymenoptera Research*, *43*, 19–44. https://doi.org/10.3897/JHR.43.8756

Ritchie, A. D., Ruppel, R., & Jha, S. (2016). Generalist Behavior Describes Pollen Foraging for Perceived Oligolectic and Polylectic Bees. *Environmental Entomology*, *45*(4), 909–919. https://doi.org/10.1093/ee/nvw032

Roberts, H. P., King, D. I., & Milam, J. (2017). Factors affecting bee communities in forest openings and adjacent mature forest. *Forest Ecology and Management*, *394*, 111–122. https://doi.org/10.1016/j.foreco.2017.03.027

Robertson, A. R., Finch, J. T. D., Young, A. D., Spooner-Hart, R. N., Outim, S. K. M., & Cook, J. M. (2020). Species diversity in bee flies and hover flies (Diptera: Bombyliidae and Syrphidae) in the horticultural environments of the Blue Mountains, Australia. *Austral Entomology*, *59*(3), 561–571. https://doi.org/10.1111/aen.12483

Rochlin, I., White, G., Reissen, N., Martheswaran, T., & Faraji, A. (2022). Effects of aerial adulticiding for mosquito management on nontarget insects: A Bayesian and community ecology approach. *Ecosphere*, *13*(1), e3896. https://doi.org/10.1002/ecs2.3896

Rodrigo Gómez, S., Ornosa, C., García Gila, J., Blasco-Aróstegui, J., Selfa, J., Guara, M., & Polidori, C. (2021). Bees and crops in Spain: An update for melon, watermelon and almond. *Annales De La Société Entomologique De France*, *57*(1), 12–28. https://doi.org/10.1080/00379271.2020.1847191

Rodrigo Gómez, S., Ornosa, C., Selfa, J., Guara, M., & Polidori, C. (2016). Small sweat bees (Hymenoptera: Halictidae) as potential major pollinators of melon (Cucumis melo ) in the Mediterranean. *Entomological Science*, *19*(1), 55–66. https://doi.org/10.1111/ens.12168

Rodríguez, A., & Kouki, J. (2015). Emulating natural disturbance in forest management enhances pollination services for dominant Vaccinium shrubs in boreal pine-dominated forests. *Forest Ecology and Management*, *350*, 1–12. https://doi.org/10.1016/j.foreco.2015.04.029

Rogers, S. R., Tarpy, D. R., & Burrack, H. J. (2014). Bee Species Diversity Enhances Productivity and Stability in a Perennial Crop. *PLOS ONE*, *9*(5), e97307. https://doi.org/10.1371/journal.pone.0097307

Ropars, L., Affre, L., Aubert, M., Fernandez, C., Flacher, F., Genoud, D., . . . Geslin, B. (2020). Pollinator Specific Richness and Their Interactions With Local Plant Species: 10 Years of Sampling in Mediterranean Habitats. *Environmental Entomology*, *49*(4), 947–955. https://doi.org/10.1093/ee/nvaa061

Rösch, M. (2005). Pollen analysis of the contents of excavated vessels—Direct archaeobotanical evidence of beverages. *Vegetation History and Archaeobotany*, *14*(3), 179–188. https://doi.org/10.1007/s00334-005-0015-9

Rothwell, A., & Ginsberg, H. S. (2019). The Bee Fauna of Coastal Napatree Point and Two Inland Sites in Southern Rhode Island. *Northeastern Naturalist*, *26*(3), 446–464. https://doi.org/10.1656/045.026.0301

Roulston, T. H., Smith, S. A., & Brewster, A. L. (2007). A Comparison of Pan Trap and Intensive Net Sampling Techniques for Documenting a Bee (Hymenoptera: Apiformes) Fauna. *Journal of the Kansas Entomological Society*, *80*(2), 179–181. https://doi.org/10.2317/0022-8567(2007)80[179:ACOPTA]2.0.CO;2

Rubene, D., Schroeder, M., & Ranius, T. (2015). Estimating bee and wasp (Hymenoptera: Aculeata) diversity on clear-cuts in forest landscapes - an evaluation of sampling methods. *Insect Conservation and Diversity*, *8*(3), 261–271. https://doi.org/10.1111/icad.12105

Rutgers-Kelly, A. C., & Richards, M. H. [Miriam H.] (2013). Effect of meadow regeneration on bee (Hymenoptera: Apoidea) abundance and diversity in southern Ontario, Canada. *The Canadian Entomologist*, *145*(6), 655–667. https://doi.org/10.4039/tce.2013.42

Ruttan, A., Filazzola, A., & Lortie, C. J. (2016). Shrub-annual facilitation complexes mediate insect community structure in arid environments. *Journal of Arid Environments*, *134*, 1–9. https://doi.org/10.1016/j.jaridenv.2016.06.009

Sahli, H. F., Krushelnycky, P. D., Drake, D. R., & Taylor, A. D. (2016). Patterns of Floral Visitation to Native Hawaiian Plants in Presence and Absence of Invasive Argentine Ants. *Pacific Science*, *70*(3), 309–322. https://doi.org/10.2984/70.3.3

Samnegård, U., Hambäck, P. A., Eardley, C., Nemomissa, S., & Hylander, K. (2015). Turnover in bee species composition and functional trait distributions between seasons in a tropical agricultural landscape. *Agriculture, Ecosystems & Environment*, *211*, 185–194. https://doi.org/10.1016/j.agee.2015.06.010

Sanchez, J. A., Carrasco, A., La Spina, M., Pérez-Marcos, M., & Ortiz-Sánchez, F. J. (2019). How Bees Respond Differently to Field Margins of Shrubby and Herbaceous Plants in Intensive Agricultural Crops of the Mediterranean Area. *Insects*, *11*(1), 26. https://doi.org/10.3390/insects11010026

Sardiñas, H. S., & Kremen, C. (2014). Evaluating nesting microhabitat for ground-nesting bees using emergence traps. *Basic and Applied Ecology*, *15*(2), 161–168. https://doi.org/10.1016/j.baae.2014.02.004

Saunders, M. E., & Luck, G. W. (2013). Pan trap catches of pollinator insects vary with habitat. *Australian Journal of Entomology*, *52*(2), 106–113. https://doi.org/10.1111/aen.12008

Saunders, M. E., Luck, G. W., & Mayfield, M. M. (2013). Almond orchards with living ground cover host more wild insect pollinators. *Journal of Insect Conservation*, *17*(5), 1011–1025. https://doi.org/10.1007/s10841-013-9584-6

Scherber, C., Beduschi, T., & Tscharntke, T. (2019). Novel approaches to sampling pollinators in whole landscapes: A lesson for landscape-wide biodiversity monitoring. *Landscape Ecology*, *34*(5), 1057–1067. https://doi.org/10.1007/s10980-018-0757-2

Schlueter, M. A., & Stewart, N. G. (2015). Native Bee (Hymenoptera: Apoidea) Abundance and Diversity in North Georgia Apple Orchards throughout the 2010 Growing Season (March to October). *Southeastern Naturalist*, *14*(4), 721–739. https://doi.org/10.1656/058.014.0416

Schubert, J., Bewersdorff, J., Luch, A., & Schulz, T. G. (2012). Waterpipe smoke: A considerable source of human exposure against furanic compounds. *Analytica Chimica Acta*, *709*, 105–112. https://doi.org/10.1016/j.aca.2011.10.012

Schurr, L., Geslin, B., Affre, L., Gachet, S., Delobeau, M., Brugger, M., . . . Masotti, V. (2021). Landscape and Local Drivers Affecting Flying Insects along Fennel Crops (Foeniculum vulgare, Apiaceae) and Implications for Its Yield. *Insects*, *12*(5), 404. https://doi.org/10.3390/insects12050404

Shannak, B., Corsmeier, U., Kottmeier, C., & Al-azab, T. (2014). Wind tunnel study of twelve dust samples by large particle size. *Atmospheric Environment*, *98*, 442–453. https://doi.org/10.1016/j.atmosenv.2014.08.062

Shapiro, L. H., Tepedino, V. J. [V. J.], & Minckley, R. L. (2014). Bowling for bees: Optimal sample number for "bee bowl" sampling transects. *Journal of Insect Conservation*, *18*(6), 1105–1113. https://doi.org/10.1007/s10841-014-9720-y

Shi, X., Xiao, H., Luo, S., Hodgson, J. A., Bianchi, F. J., He, H., . . . Zou, Y. (2021). Can landscape level semi-natural habitat compensate for pollinator biodiversity loss due to farmland consolidation? *Agriculture, Ecosystems & Environment*, *319*, 107519. https://doi.org/10.1016/j.agee.2021.107519

Shrestha, M., Garcia, J. E., Chua, J. H. J., Howard, S. R., Tscheulin, T., Dorin, A., . . . Dyer, A. G. (2019). Fluorescent Pan Traps Affect the Capture Rate of Insect Orders in Different Ways. *Insects*, *10*(2), 40. https://doi.org/10.3390/insects10020040

Shrestha, M., Garcia, J. E., Thomas, F., Howard, S. R., Chua, J. H. J., Tscheulin, T., . . . Dyer, A. G. (2021). Insects in the City: Does Remnant Native Habitat Influence Insect Order Distributions? *Diversity*, *13*(4), 148. https://doi.org/10.3390/d13040148

Silva, F. O., Kevan, S. D., Roque, N., Viana, B. F. [B. F.], & Kevan, P. G. [P. G.] (2010). Records on floral biology and visitors of Jacquemontia montana (Moric.) Meisn. (Convolvulaceae) in Mucugê, Bahia. *Brazilian Journal of Biology*, *70*(3), 671–676. https://doi.org/10.1590/S1519-69842010000300027

Silva, W. R., & Sazima, M. (1995). Hawkmoth pollination in Cereus peruvianus, a columnar cactus from southeastern Brazil. *Flora*, *190*(4), 339–343. https://doi.org/10.1016/S0367-2530(17)30674-6

Simon, S. J., Keefover-Ring, K., Park, Y.‑L., Wimp, G., Grady, J., & DiFazio, S. P. (2021). Characterization of Salix nigra floral insect community and activity of three native Andrena bees. *Ecology and Evolution*, *11*(9), 4688–4700. https://doi.org/10.1002/ece3.7369

Sing, K.‑W., Wang, W.‑Z., Wan, T., Lee, P.‑S., Li, Z.‑X., Chen, X., . . . Wilson, J.‑J. (2016). Diversity and human perceptions of bees (Hymenoptera: Apoidea) in Southeast Asian megacities. *Genome*, *59*(10), 827–839. https://doi.org/10.1139/gen-2015-0159

Sircom, J., Jothi, G. A., & Pinksen, J. (2018). Monitoring bee populations: Are eusocial bees attracted to different colours of pan trap than other bees? *Journal of Insect Conservation*, *22*(3-4), 433–441. https://doi.org/10.1007/s10841-018-0071-y

Sivakoff, F. S., Prajzner, S. P., & Gardiner, M. M. (2018). Unique Bee Communities within Vacant Lots and Urban Farms Result from Variation in Surrounding Urbanization Intensity. *Sustainability*, *10*(6), 1926. https://doi.org/10.3390/su10061926

Song, C. Y., Moon, J. Y., & Park, B. M. (2020). Breeding of Lilium Asiatic 'Bright Yellow LiA' as a Seed Propagation Hybrid for Garden. *Horticultural Science & Technology*, *38*(5), 754–761. https://doi.org/10.7235/HORT.20200068

Souza, L. de [Luceli], & Campos, M. J. d. O. (2008). Composition and diversity of bees (Hymenoptera) attracted by Moericke traps in an agricultural area in Rio Claro, state of São Paulo, Brasil. *Iheringia Série Zoologia*, *98*(2), 236–243. https://doi.org/10.1590/S0073-47212008000200012

Spafford, R. D., & Lortie, C. J. (2013). Sweeping beauty: Is grassland arthropod community composition effectively estimated by sweep netting? *Ecology and Evolution*, *3*(10), 3347–3358. https://doi.org/10.1002/ece3.688

St. Clair, A. L., Dolezal, A. G., O'Neal, M. E. [Matthew E.], & Toth, A. L. (2020). Pan Traps for Tracking Honey Bee Activity-Density: A Case Study in Soybeans. *Insects*, *11*(6), 366. https://doi.org/10.3390/insects11060366

Stein, K., Stenchly, K., Coulibaly, D., Pauly, A., Dimobe, K., Steffan-Dewenter, I., . . . Linsenmair, K. E. (2018). Impact of human disturbance on bee pollinator communities in savanna and agricultural sites in Burkina Faso, West Africa. *Ecology and Evolution*, *8*(13), 6827–6838. https://doi.org/10.1002/ece3.4197

Stephenson, P. L., Dowling, A. P. G., & Krementz, D. G. (2020). Bee Communities of Emergent Wetlands Under Restoration in the Lower Mississippi Alluvial Valley of Arkansas. *Southeastern Naturalist*, *19*(3), 472–490. https://doi.org/10.1656/058.019.0303

Stephenson, P. L., Griswold, T. L., Arduser, M. S., Dowling, A. P. G., & Krementz, D. G. (2018). Checklist of bees (Hymenoptera: Apoidea) from managed emergent wetlands in the lower Mississippi Alluvial Valley of Arkansas. *Biodiversity Data Journal*, *6*, e24071. https://doi.org/10.3897/BDJ.6.e24071

Straka, J. [J.], Rezkova, K., Batelka, J., & Kratochvíl, L. (2011). Early nest emergence of females parasitised by Strepsiptera in protandrous bees (Hymenoptera Andrenidae). *Ethology Ecology & Evolution*, *23*(2), 97–109. https://doi.org/10.1080/03949370.2011.554880

Streinzer, M., Roth, N., Paulus, H. F., & Spaethe, J. (2019). Color preference and spatial distribution of glaphyrid beetles suggest a key role in the maintenance of the color polymorphism in the peacock anemone (Anemone pavonina, Ranunculaceae) in Northern Greece. *Journal of Comparative Physiology a*, *205*(5), 735–743. https://doi.org/10.1007/s00359-019-01360-2

Suárez, N. F., Latini, A. O., Rufini, J. C. M., Reis, L. A. C., Silva, D. P. [Daniel Paiva], Abreu, R. A. A., & Stein, K. (2022). Unusual pollinator attractants increase the fructification rate on West Indian Cherry Trees. *JOURNAL of APPLIED ENTOMOLOGY*, *146*(3), 284–290. https://doi.org/10.1111/jen.12964

Szczepko, K., Kruk, A., & Wiśniowski, B. (2020). Local habitat conditions shaping the assemblages of vespid wasps (Hymenoptera: Vespidae) in a post-agricultural landscape of the Kampinos National Park in Poland. *Scientific Reports*, *10*(1), 1424. https://doi.org/10.1038/s41598-020-57426-8

Taki, H., Kevan, P. G. [Peter G.], & Ascher, J. S. [John S.] (2007). Landscape effects of forest loss in a pollination system. *Landscape Ecology*, *22*(10), 1575–1587. https://doi.org/10.1007/s10980-007-9153-z

Talašová, A., Straka, J. [Jakub], Hadrava, J., Benda, D., Kocourek, F., & Kazda, J. (2018). High degree of philopatry is required for mobile insects used as local indicators in biodiversity studies. *Ecological Indicators*, *94*, 99–103. https://doi.org/10.1016/j.ecolind.2018.06.008

Tang, M., Hardman, C. J., Ji, Y., Meng, G., Liu, S., Tan, M., . . . Yu, D. W. (2015). High-throughput monitoring of wild bee diversity and abundance via mitogenomics. *Methods in Ecology and Evolution*, *6*(9), 1034–1043. https://doi.org/10.1111/2041-210X.12416

Templ, B., Mózes, E., Templ, M., Földesi, R., Szirák, Á., Báldi, A., & Kovács-Hostyánszki, A. (2019). Habitat-dependency of transect walk and pan trap methods for bee sampling in farmlands. *Journal of Apicultural Science*, *63*(1), 93–115. https://doi.org/10.2478/JAS-2019-0014

Thompson, A., Frenzel, M., Schweiger, O., Musche, M., Groth, T., Roberts, S. P., . . . Knight, T. M. (2021). Pollinator sampling methods influence community patterns assessments by capturing species with different traits and at different abundances. *Ecological Indicators*, *132*, 108284. https://doi.org/10.1016/j.ecolind.2021.108284

Thompson, H., Schneider, C., Maus, C., Camata, C., & Wolf, C. (2020). Prevalence and abundance of bees visiting major conventionally-managed agricultural crops in Brazil. *Journal of Apicultural Research*, *59*(2), 246–260. https://doi.org/10.1080/00218839.2019.1655132

Todd, K. J., Gardiner, M. M., & Lindquist, E. D. (2016). Mass Flowering Crops as a Conservation Resource for Wild Pollinators (Hymenoptera: Apoidea). *Journal of the Kansas Entomological Society*, *89*(2), 158–167. https://doi.org/10.2317/0022-8567-89.2.158

Toler, T. R., Evans, E. W., & Tepedino, V. J. [Vincent J.] (2005). Pan-trapping for bees (Hymenoptera : Apiformes) in Utah's West Desert: The importance of color diversity. *Pan-Pacific Entomologist*, *81*(3-4), 103–113.

Torné-Noguera, A., Rodrigo, A., Osorio, S., & Bosch, J. (2016). Collateral effects of beekeeping: Impacts on pollen-nectar resources and wild bee communities. *Basic and Applied Ecology*, *17*(3), 199–209. https://doi.org/10.1016/j.baae.2015.11.004

Török, E., Gallé, R., & Batáry, P. (2022). Fragmentation of forest-steppe predicts functional community composition of wild bee and wasp communities. *Global Ecology and Conservation*, *33*, e01988. https://doi.org/10.1016/j.gecco.2021.e01988

Travis, D. J., & Gonzalez, V. H. [V. H.] (2017). Bee Stratification in Western Turkey: Pan Trap Color, Height, and Habitat Preferences. *Integrative and Comparative Biology*, *57*, E169-E169. Retrieved from https://sicb.org/abstracts/bee-stratification-in-western-turkey-pan-trap-color-height-and-habitat-preferences/

Tuell, J. K., Ascher, J. S. [John S.], & Isaacs, R. (2009). Wild Bees (Hymenoptera: Apoidea: Anthophila) of the Michigan Highbush Blueberry Agroecosystem. *Annals of the Entomological Society of America*, *102*(2), 275–287. https://doi.org/10.1603/008.102.0209

Tuell, J. K., & Isaacs, R. (2009). Elevated pan traps to monitor bees in flowering crop canopies. *Entomologia Experimentalis Et Applicata*, *131*(1), 93–98. https://doi.org/10.1111/j.1570-7458.2009.00826.x

Tuell, J. K., & Isaacs, R. (2010). Community and Species-Specific Responses of Wild Bees to Insect Pest Control Programs Applied to a Pollinator-Dependent Crop. *Journal of Economic Entomology*, *103*(3), 668–675. https://doi.org/10.1603/EC09314

Turo, K. J., Spring, M. R., Sivakoff, F. S., Delgado de la flor, Yvan A., & Gardiner, M. M. (2021). Conservation in post‐industrial cities: How does vacant land management and landscape configuration influence urban bees? *Journal of Applied Ecology*, *58*(1), 58–69. https://doi.org/10.1111/1365-2664.13773

Uemori, K., Mita, T., & Hishi, T. (2021). Changes in species, functional, and phylogenetic diversity of Aculeata communities along elevational gradients in the Kyushu Central Mountains of the Japanese archipelago. *Ecological Research*, *36*(5), 778–787. https://doi.org/10.1111/1440-1703.12242

Ugolini, A., Vignali, B., Castellini, C., & Lindström, M. (1996). Zonal orientation and spectral filtering in Talitrus saltator (Amphipoda, Talitridae). *Journal of the Marine Biological Association of the United Kingdom*, *76*(2), 377–389. https://doi.org/10.1017/S0025315400030617

Ulyshen, M. D., Horn, S., & Hanula, J. L. [James L.] (2022). Decadal Patterns of Forest and Pollinator Recovery Following the Eradication of an Invasive Shrub. *Frontiers in Ecology and Evolution*, *10.* https://doi.org/10.3389/fevo.2022.832268

Ulyshen, M. D., Pokswinski, S., & Hiers, J. K. (2020). A comparison of bee communities between primary and mature secondary forests in the longleaf pine ecosystem. *Scientific Reports*, *10*(1), 2916. https://doi.org/10.1038/s41598-020-59878-4

Ulyshen, M. D., Soon, V., & Hanula, J. L. [James L.] (2010). On the vertical distribution of bees in a temperate deciduous forest. *Insect Conservation and Diversity*, *3*(3), 222–228. https://doi.org/10.1111/j.1752-4598.2010.00092.x

Umair Sial, M., Zeeshan Majeed, M., Atiq, A., Farooq, T., Aatif, H. M., Jaleel, W., . . . Ghrmah, H. A. (2022). Differential efficacy of edaphic traps for monitoring arthropods diversity in subtropical regions. *Journal of King Saud University - Science*, *34*(1), 101686. https://doi.org/10.1016/j.jksus.2021.101686

Urban-Mead, K. R., Muñiz, P., Gillung, J., Espinoza, A., Fordyce, R., van Dyke, M., . . . Danforth, B. N. (2021). Bees in the trees: Diverse spring fauna in temperate forest edge canopies. *Forest Ecology and Management*, *482*, 118903. https://doi.org/10.1016/j.foreco.2020.118903

Venjakob, C., Ruedenauer, F. A., Klein, A.‑M. [A.-M.], & Leonhardt, S. D. (2022). Variation in nectar quality across 34 grassland plant species. *Plant Biology*, *24*(1), 134–144. https://doi.org/10.1111/plb.13343

Vereecken, N. J., Weekers, T., Leclercq, N., Greef, S. de, Hainaut, H., Molenberg, J.‑M., . . . Marshall, L. (2021). Insect biomass is not a consistent proxy for biodiversity metrics in wild bees. *Ecological Indicators*, *121*, 107132. https://doi.org/10.1016/j.ecolind.2020.107132

Vickruck, J. L., Rehan, S. M. [S. M.], Sheffield, C. S. [C. S.], & Richards, M. H. [M. H.] (2011). Nesting biology and DNA barcode analysis of Ceratina dupla and C. Mikmaqi, and comparisons with C. Calcarata (Hymenoptera: Apidae: Xylocopinae). *The Canadian Entomologist*, *143*(3), 254–262. https://doi.org/10.4039/n11-006

Vieira, C. R., Waichert, C., Williams, K. A., & Pitts, J. P. (2017). Evaluation of Malaise and Yellow Pan Trap Performance to Assess Velvet Ant (Hymenoptera: Mutillidae) Diversity in a Neotropical Savanna. *Environmental Entomology*, *46*(2), 353–361. https://doi.org/10.1093/ee/nvx046

Vrdoljak, S. M., & Samways, M. J. (2012). Optimising coloured pan traps to survey flower visiting insects. *Journal of Insect Conservation*, *16*(3), 345–354. https://doi.org/10.1007/s10841-011-9420-9

Wagner, D. L., Ascher, J. S. [John S.], & Bricker, N. K. (2014). A Transmission Right-of-Way as Habitat for Wild Bees (Hymenoptera: Apoidea: Anthophila) in Connecticut. *Annals of the Entomological Society of America*, *107*(6), 1110–1120. https://doi.org/10.1603/AN14001

Wang, X., Hua, F., Wang, L., Wilcove, D. S., & Yu, D. W. (2019). The biodiversity benefit of native forests and mixed-species plantations over monoculture plantations. *Diversity and Distributions*, *25*(11), 1721–1735. https://doi.org/10.1111/ddi.12972

Watson, J. C., Wolf, A. T., & Ascher, J. S. [J. S.] (2011). Forested Landscapes Promote Richness and Abundance of Native Bees (Hymenoptera: Apoidea: Anthophila) in Wisconsin Apple Orchards. *Environmental Entomology*, *40*(3), 621–632. https://doi.org/10.1603/EN10231

Wester, P., Stanway, R., & Pauw, A. (2009). Mice pollinate the Pagoda Lily, Whiteheadia bifolia (Hyacinthaceae) - First field observations with photographic documentation of rodent pollination in South Africa. *South African Journal of Botany*, *75*(4), 713–719. https://doi.org/10.1016/j.sajb.2009.07.005

Westerberg, L., Berglund, H.‑L., Jonason, D., & Milberg, P. (2021). Color pan traps often catch less when there are more flowers around. *Ecology and Evolution*, *11*(9), 3830–3840. https://doi.org/10.1002/ece3.7252

Westphal, C., Bommarco, R., Carré, G., Lamborn, E., Morison, N., Petanidou, T., . . . Steffan-Dewenter, I. (2008). Measuring bee diversity in different European habitats and biogeographical regions. *Ecological Monographs*, *78*(4), 653–671. https://doi.org/10.1890/07-1292.1

Wheelock, M. J., & O'Neal, M. E. [M. E.] (2016). Insect Pollinators in Iowa Cornfields: Community Identification and Trapping Method Analysis. *PLOS ONE*, *11*(7), e0143479. https://doi.org/10.1371/journal.pone.0143479

Wheelock, M. J., Rey, K. P., & O'Neal, M. E. [M. E.] (2016). Defining the Insect Pollinator Community Found in Iowa Corn and Soybean Fields: Implications for Pollinator Conservation. *Environmental Entomology*, *45*(5), 1099–1106. https://doi.org/10.1093/ee/nvw087

Wilson, J. S., Griswold, T., & Messinger, O. J. (2008). Sampling Bee Communities (Hymenoptera: Apiformes) in a Desert Landscape: Are Pan Traps Sufficient? *Journal of the Kansas Entomological Society*, *81*(3), 288–300. https://doi.org/10.2317/JKES-802.06.1

Wilson, J. S., Jahner, J. P., Starley, L., Calvin, C. L., Ikerd, H., & Griswold, T. (2016). Sampling bee communities using pan traps: Alternative methods increase sample size. *Journal of Insect Conservation*, *20*(5), 919–922. https://doi.org/10.1007/s10841-016-9914-6

Wood, T. J., Holland, J. M., & Goulson, D. [D.] (2015). A comparison of techniques for assessing farmland bumblebee populations. *Oecologia*, *177*(4), 1093–1102. https://doi.org/10.1007/s00442-015-3255-0

Zhu, X., Yang, J., & Li, Q. (2013). Alpine Codonopsis convolvulacea (Campanulaceae) provides multiple rewards to its main pollinator. *Plant Ecology & Diversity*, *6*(2), 187–193. https://doi.org/10.1080/17550874.2013.771713

Zou, Y., Bianchi, F. J., Jauker, F., Xiao, H., Chen, J., Cresswell, J., . . . van der Werf, W. (2017). Landscape effects on pollinator communities and pollination services in small-holder agroecosystems. *Agriculture, Ecosystems & Environment*, *246*, 109–116. https://doi.org/10.1016/j.agee.2017.05.035

Zou, Y., Xiao, H., Bianchi, Felix J. J. A., Jauker, F., Luo, S., & van der Werf, W. (2017). Wild pollinators enhance oilseed rape yield in small-holder farming systems in China. *BMC Ecology*, *17*(1), 6. https://doi.org/10.1186/s12898-017-0116-1

Zumpf, C., Quinn, J., Cacho, J., Grasse, N., Negri, M. C., & Lee, D. (2021). Invertebrate and Plant Community Diversity of an Illinois Corn–Soybean Field with Integrated Shrub Willow Bioenergy Buffers. *Sustainability*, *13*(21), 12280. https://doi.org/10.3390/su132112280
